# Repositioning of the global epicentre of non-optimal cholesterol

**DOI:** 10.1038/s41586-020-2338-1

**Published:** 2020-06-03

**Authors:** NCD Risk Factor Collaboration (NCD-RisC), Cristina Taddei, Bin Zhou, Honor Bixby, Rodrigo M. Carrillo-Larco, Goodarz Danaei, Rod T. Jackson, Farshad Farzadfar, Marisa K. Sophiea, Mariachiara Di Cesare, Maria Laura Caminia Iurilli, Andrea Rodriguez Martinez, Golaleh Asghari, Klodian Dhana, Pablo Gulayin, Sujay Kakarmath, Marilina Santero, Trudy Voortman, Leanne M. Riley, Melanie J. Cowan, Stefan Savin, James E. Bennett, Gretchen A. Stevens, Christopher J. Paciorek, Wichai Aekplakorn, Renata Cifkova, Simona Giampaoli, Andre Pascal Kengne, Young-Ho Khang, Kari Kuulasmaa, Avula Laxmaiah, Paula Margozzini, Prashant Mathur, Børge G. Nordestgaard, Dong Zhao, Mette Aadahl, Leandra Abarca-Gómez, Hanan Abdul Rahim, Niveen M. Abu-Rmeileh, Benjamin Acosta-Cazares, Robert J. Adams, Imelda A. Agdeppa, Javad Aghazadeh-Attari, Carlos A. Aguilar-Salinas, Charles Agyemang, Tarunveer S. Ahluwalia, Noor Ani Ahmad, Ali Ahmadi, Naser Ahmadi, Soheir H. Ahmed, Wolfgang Ahrens, Kamel Ajlouni, Monira Alarouj, Fadia AlBuhairan, Shahla AlDhukair, Mohamed M. Ali, Abdullah Alkandari, Ala’a Alkerwi, Eman Aly, Deepak N. Amarapurkar, Philippe Amouyel, Lars Bo Andersen, Sigmund A. Anderssen, Ranjit Mohan Anjana, Alireza Ansari-Moghaddam, Hajer Aounallah-Skhiri, Joana Araújo, Inger Ariansen, Tahir Aris, Raphael E. Arku, Nimmathota Arlappa, Krishna K. Aryal, Thor Aspelund, Maria Cecília F. Assunção, Juha Auvinen, Mária Avdicová, Ana Azevedo, Fereidoun Azizi, Mehrdad Azmin, Nagalla Balakrishna, Mohamed Bamoshmoosh, Maciej Banach, Piotr Bandosz, José R. Banegas, Carlo M. Barbagallo, Alberto Barceló, Amina Barkat, Iqbal Bata, Anwar M. Batieha, Assembekov Batyrbek, Louise A. Baur, Robert Beaglehole, Antonisamy Belavendra, Habiba Ben Romdhane, Mikhail Benet, Marianne Benn, Salim Berkinbayev, Antonio Bernabe-Ortiz, Gailute Bernotiene, Heloisa Bettiol, Santosh K. Bhargava, Yufang Bi, Asako Bienek, Mukharram Bikbov, Bihungum Bista, Peter Bjerregaard, Espen Bjertness, Marius B. Bjertness, Cecilia Björkelund, Katia V. Bloch, Anneke Blokstra, Simona Bo, Bernhard O. Boehm, Jose G. Boggia, Carlos P. Boissonnet, Marialaura Bonaccio, Vanina Bongard, Rossana Borchini, Herman Borghs, Pascal Bovet, Imperia Brajkovich, Juergen Breckenkamp, Hermann Brenner, Lizzy M. Brewster, Graziella Bruno, Anna Bugge, Markus A. Busch, Antonio Cabrera de León, Joseph Cacciottolo, Günay Can, Ana Paula C. Cândido, Mario V. Capanzana, Eduardo Capuano, Vincenzo Capuano, Viviane C. Cardoso, Joana Carvalho, Felipe F. Casanueva, Laura Censi, Charalambos A. Chadjigeorgiou, Snehalatha Chamukuttan, Nish Chaturvedi, Chien-Jen Chen, Fangfang Chen, Shuohua Chen, Ching-Yu Cheng, Bahman Cheraghian, Angela Chetrit, Shu-Ti Chiou, María-Dolores Chirlaque, Belong Cho, Yumi Cho, Jerzy Chudek, Frank Claessens, Janine Clarke, Els Clays, Hans Concin, Susana C. Confortin, Cyrus Cooper, Simona Costanzo, Dominique Cottel, Chris Cowell, Ana B. Crujeiras, Semánová Csilla, Liufu Cui, Felipe V. Cureau, Graziella D’Arrigo, Eleonora d’Orsi, Jean Dallongeville, Albertino Damasceno, Rachel Dankner, Thomas M. Dantoft, Luc Dauchet, Kairat Davletov, Guy De Backer, Dirk De Bacquer, Giovanni de Gaetano, Stefaan De Henauw, Paula Duarte de Oliveira, David De Ridder, Delphine De Smedt, Mohan Deepa, Alexander D. Deev, Abbas Dehghan, Hélène Delisle, Elaine Dennison, Valérie Deschamps, Meghnath Dhimal, Augusto F. Di Castelnuovo, Zivka Dika, Shirin Djalalinia, Annette J. Dobson, Chiara Donfrancesco, Silvana P. Donoso, Angela Döring, Maria Dorobantu, Nico Dragano, Wojciech Drygas, Yong Du, Charmaine A. Duante, Rosemary B. Duda, Vilnis Dzerve, Elzbieta Dziankowska-Zaborszczyk, Ricky Eddie, Ebrahim Eftekhar, Robert Eggertsen, Sareh Eghtesad, Gabriele Eiben, Ulf Ekelund, Jalila El Ati, Denise Eldemire-Shearer, Marie Eliasen, Roberto Elosua, Rajiv T. Erasmus, Raimund Erbel, Cihangir Erem, Louise Eriksen, Johan G. Eriksson, Jorge Escobedo-de la Peña, Saeid Eslami, Ali Esmaeili, Alun Evans, David Faeh, Caroline H. Fall, Elnaz Faramarzi, Mojtaba Farjam, Mohammad Reza Fattahi, Francisco J. Felix-Redondo, Trevor S. Ferguson, Daniel Fernández-Bergés, Daniel Ferrante, Marika Ferrari, Catterina Ferreccio, Jean Ferrieres, Bernhard Föger, Leng Huat Foo, Ann-Sofie Forslund, Maria Forsner, Heba M. Fouad, Damian K. Francis, Maria do Carmo Franco, Oscar H. Franco, Guillermo Frontera, Yuki Fujita, Matsuda Fumihiko, Takuro Furusawa, Zbigniew Gaciong, Fabio Galvano, Jingli Gao, Manoli Garcia-de-la-Hera, Sarah P. Garnett, Jean-Michel Gaspoz, Magda Gasull, Andrea Gazzinelli, Johanna M. Geleijnse, Ali Ghanbari, Erfan Ghasemi, Oana-Florentina Gheorghe-Fronea, Anup Ghimire, Francesco Gianfagna, Tiffany K. Gill, Jonathan Giovannelli, Glen Gironella, Aleksander Giwercman, David Goltzman, Helen Gonçalves, David A. Gonzalez-Chica, Marcela Gonzalez-Gross, Juan P. González-Rivas, Clicerio González-Villalpando, María-Elena González-Villalpando, Angel R. Gonzalez, Frederic Gottrand, Sidsel Graff-Iversen, Dušan Grafnetter, Ronald D. Gregor, Tomasz Grodzicki, Anders Grøntved, Giuseppe Grosso, Gabriella Gruden, Dongfeng Gu, Pilar Guallar-Castillón, Ong Peng Guan, Elias F. Gudmundsson, Vilmundur Gudnason, Ramiro Guerrero, Idris Guessous, Johanna Gunnlaugsdottir, Rajeev Gupta, Laura Gutierrez, Felix Gutzwiller, Seongjun Ha, Farzad Hadaegh, Rosa Haghshenas, Hamid Hakimi, Ian R. Hambleton, Behrooz Hamzeh, Sari Hantunen, Rachakulla Hari Kumar, Seyed Mohammad Hashemi-Shahri, Jun Hata, Teresa Haugsgjerd, Alison J. Hayes, Jiang He, Yuna He, Marleen Elisabeth Hendriks, Ana Henriques, Sauli Herrala, Ramin Heshmat, Allan G. Hill, Sai Yin Ho, Suzanne C. Ho, Michael Hobbs, Albert Hofman, Reza Homayounfar, Wilma M. Hopman, Andrea R. V. R. Horimoto, Claudia M. Hormiga, Bernardo L. Horta, Leila Houti, Christina Howitt, Thein Thein Htay, Aung Soe Htet, Maung Maung Than Htike, José María Huerta, Ilpo Tapani Huhtaniemi, Martijn Huisman, Monica L. Hunsberger, Abdullatif S. Husseini, Inge Huybrechts, Nahla Hwalla, Licia Iacoviello, Anna G. Iannone, Mohsen M. Ibrahim, Norazizah Ibrahim Wong, Iris Iglesia, Nayu Ikeda, M. Arfan Ikram, Violeta Iotova, Vilma E. Irazola, Takafumi Ishida, Muhammad Islam, Aziz al-Safi Ismail, Masanori Iwasaki, Jeremy M. Jacobs, Hashem Y. Jaddou, Tazeen Jafar, Kenneth James, Konrad Jamrozik, Imre Janszky, Edward Janus, Marjo-Riitta Jarvelin, Grazyna Jasienska, Ana Jelakovic, Bojan Jelakovic, Garry Jennings, Gorm B. Jensen, Seung-lyeal Jeong, Anjani Kumar Jha, Chao Qiang Jiang, Ramon O. Jimenez, Karl-Heinz Jöckel, Michel Joffres, Jari J. Jokelainen, Jost B. Jonas, Torben Jørgensen, Pradeep Joshi, Farahnaz Joukar, Jacek Józwiak, Anne Juolevi, Anthony Kafatos, Eero O. Kajantie, Ofra Kalter-Leibovici, Nor Azmi Kamaruddin, Pia R. Kamstrup, Khem B. Karki, Joanne Katz, Jussi Kauhanen, Prabhdeep Kaur, Maryam Kavousi, Gyulli Kazakbaeva, Ulrich Keil, Sirkka Keinänen-Kiukaanniemi, Roya Kelishadi, Maryam Keramati, Alina Kerimkulova, Mathilde Kersting, Yousef Saleh Khader, Davood Khalili, Mohammad Khateeb, Motahareh Kheradmand, Alireza Khosravi, Ursula Kiechl-Kohlendorfer, Stefan Kiechl, Japhet Killewo, Hyeon Chang Kim, Jeongseon Kim, Yeon-Yong Kim, Jurate Klumbiene, Michael Knoflach, Stephanie Ko, Hans-Peter Kohler, Iliana V. Kohler, Elin Kolle, Patrick Kolsteren, Jürgen König, Raija Korpelainen, Paul Korrovits, Jelena Kos, Seppo Koskinen, Katsuyasu Kouda, Sudhir Kowlessur, Wolfgang Kratzer, Susi Kriemler, Peter Lund Kristensen, Steiner Krokstad, Daan Kromhout, Urho M. Kujala, Pawel Kurjata, Catherine Kyobutungi, Fatima Zahra Laamiri, Tiina Laatikainen, Carl Lachat, Youcef Laid, Tai Hing Lam, Christina-Paulina Lambrinou, Vera Lanska, Georg Lappas, Bagher Larijani, Tint Swe Latt, Lars E. Laugsand, Maria Lazo-Porras, Jeannette Lee, Jeonghee Lee, Nils Lehmann, Terho Lehtimäki, Naomi S. Levitt, Yanping Li, Christa L. Lilly, Wei-Yen Lim, M. Fernanda Lima-Costa, Hsien-Ho Lin, Xu Lin, Yi-Ting Lin, Lars Lind, Allan Linneberg, Lauren Lissner, Jing Liu, Helle-Mai Loit, Esther Lopez-Garcia, Tania Lopez, Paulo A. Lotufo, José Eugenio Lozano, Dalia Luksiene, Annamari Lundqvist, Robert Lundqvist, Nuno Lunet, Guansheng Ma, George L. L. Machado-Coelho, Aristides M. Machado-Rodrigues, Suka Machi, Ahmed A. Madar, Stefania Maggi, Dianna J. Magliano, Emmanuella Magriplis, Gowri Mahasampath, Bernard Maire, Marcia Makdisse, Fatemeh Malekzadeh, Reza Malekzadeh, Kodavanti Mallikharjuna Rao, Yannis Manios, Jim I. Mann, Fariborz Mansour-Ghanaei, Enzo Manzato, Pedro Marques-Vidal, Reynaldo Martorell, Luis P. Mascarenhas, Ellisiv B. Mathiesen, Tandi E. Matsha, Christina Mavrogianni, Shelly R. McFarlane, Stephen T. McGarvey, Stela McLachlan, Rachael M. McLean, Scott B. McLean, Breige A. McNulty, Sounnia Mediene-Benchekor, Parinaz Mehdipour, Kirsten Mehlig, Amir Houshang Mehrparvar, Aline Meirhaeghe, Christa Meisinger, Ana Maria B. Menezes, Geetha R. Menon, Shahin Merat, Alibek Mereke, Indrapal I. Meshram, Patricia Metcalf, Haakon E. Meyer, Jie Mi, Nathalie Michels, Jody C. Miller, Cláudia S. Minderico, G. K. Mini, Juan Francisco Miquel, J. Jaime Miranda, Mohammad Reza Mirjalili, Erkin Mirrakhimov, Pietro A. Modesti, Sahar Saeedi Moghaddam, Bahram Mohajer, Mostafa K. Mohamed, Kazem Mohammad, Zahra Mohammadi, Noushin Mohammadifard, Reza Mohammadpourhodki, Viswanathan Mohan, Salim Mohanna, Muhammad Fadhli Mohd Yusoff, Iraj Mohebbi, Farnam Mohebi, Marie Moitry, Line T. Møllehave, Niels C. Møller, Dénes Molnár, Amirabbas Momenan, Charles K. Mondo, Eric Monterrubio-Flores, Mahmood Moosazadeh, Alain Morejon, Luis A. Moreno, Karen Morgan, Suzanne N. Morin, George Moschonis, Malgorzata Mossakowska, Aya Mostafa, Jorge Mota, Mohammad Esmaeel Motlagh, Jorge Motta, Kelias P. Msyamboza, Maria L. Muiesan, Martina Müller-Nurasyid, Jaakko Mursu, Norlaila Mustafa, Iraj Nabipour, Shohreh Naderimagham, Gabriele Nagel, Balkish M. Naidu, Farid Najafi, Harunobu Nakamura, Jana Námešná, Ei Ei K. Nang, Vinay B. Nangia, Matthias Nauck, William A. Neal, Azim Nejatizadeh, Ilona Nenko, Flavio Nervi, Nguyen D. Nguyen, Quang Ngoc Nguyen, Ramfis E. Nieto-Martínez, Thomas Nihal, Teemu J. Niiranen, Guang Ning, Toshiharu Ninomiya, Marianna Noale, Oscar A. Noboa, Davide Noto, Mohannad Al Nsour, Irfan Nuhoğlu, Terence W. O’Neill, Dermot O’Reilly, Angélica M. Ochoa-Avilés, Kyungwon Oh, Ryutaro Ohtsuka, Örn Olafsson, Valérie Olié, Isabel O. Oliveira, Mohd Azahadi Omar, Altan Onat, Sok King Ong, Pedro Ordunez, Rui Ornelas, Pedro J. Ortiz, Clive Osmond, Sergej M. Ostojic, Afshin Ostovar, Johanna A. Otero, Ellis Owusu-Dabo, Fred Michel Paccaud, Elena Pahomova, Andrzej Pajak, Luigi Palmieri, Wen-Harn Pan, Songhomitra Panda-Jonas, Francesco Panza, Winsome R. Parnell, Nikhil D. Patel, Nasheeta Peer, Sergio Viana Peixoto, Markku Peltonen, Alexandre C. Pereira, Annette Peters, Astrid Petersmann, Janina Petkeviciene, Niloofar Peykari, Son Thai Pham, Rafael N. Pichardo, Iris Pigeot, Aida Pilav, Lorenza Pilotto, Aleksandra Piwonska, Andreia N. Pizarro, Pedro Plans-Rubió, Silvia Plata, Hermann Pohlabeln, Miquel Porta, Marileen L. P. Portegies, Anil Poudyal, Farhad Pourfarzi, Hossein Poustchi, Rajendra Pradeepa, Jacqueline F. Price, Rui Providencia, Jardena J. Puder, Soile E. Puhakka, Margus Punab, Mostafa Qorbani, Tran Quoc Bao, Ricardas Radisauskas, Salar Rahimikazerooni, Olli Raitakari, Sudha Ramachandra Rao, Ambady Ramachandran, Elisabete Ramos, Rafel Ramos, Lekhraj Rampal, Sanjay Rampal, Josep Redon, Paul Ferdinand M. Reganit, Luis Revilla, Abbas Rezaianzadeh, Robespierre Ribeiro, Adrian Richter, Fernando Rigo, Tobias F. Rinke de Wit, Fernando Rodríguez-Artalejo, María del Cristo Rodriguez-Perez, Laura A. Rodríguez-Villamizar, Ulla Roggenbuck, Rosalba Rojas-Martinez, Dora Romaguera, Elisabetta L. Romeo, Annika Rosengren, Joel G. R. Roy, Adolfo Rubinstein, Jean-Bernard Ruidavets, Blanca Sandra Ruiz-Betancourt, Paola Russo, Petra Rust, Marcin Rutkowski, Charumathi Sabanayagam, Harshpal S. Sachdev, Alireza Sadjadi, Ali Reza Safarpour, Saeid Safiri, Olfa Saidi, Nader Saki, Benoit Salanave, Diego Salmerón, Veikko Salomaa, Jukka T. Salonen, Massimo Salvetti, Jose Sánchez-Abanto, Susana Sans, Alba M. Santaliestra-Pasías, Diana A. Santos, Maria Paula Santos, Rute Santos, Jouko L. Saramies, Luis B. Sardinha, Nizal Sarrafzadegan, Kai-Uwe Saum, Savvas C. Savva, Norie Sawada, Mariana Sbaraini, Marcia Scazufca, Beatriz D. Schaan, Herman Schargrodsky, Christa Scheidt-Nave, Anja Schienkiewitz, Sabine Schipf, Carsten O. Schmidt, Ben Schöttker, Sara Schramm, Sylvain Sebert, Aye Aye Sein, Abhijit Sen, Sadaf G. Sepanlou, Jennifer Servais, Ramin Shakeri, Svetlana A. Shalnova, Teresa Shamah-Levy, Maryam Sharafkhah, Sanjib K. Sharma, Jonathan E. Shaw, Amaneh Shayanrad, Zumin Shi, Kenji Shibuya, Hana Shimizu-Furusawa, Dong Wook Shin, Youchan Shin, Majid Shirani, Rahman Shiri, Namuna Shrestha, Khairil Si-Ramlee, Alfonso Siani, Rosalynn Siantar, Abla M. Sibai, Diego Augusto Santos Silva, Mary Simon, Judith Simons, Leon A. Simons, Michael Sjöström, Tea Skaaby, Jolanta Slowikowska-Hilczer, Przemyslaw Slusarczyk, Liam Smeeth, Marieke B. Snijder, Stefan Söderberg, Agustinus Soemantri, Reecha Sofat, Vincenzo Solfrizzi, Mohammad Hossein Somi, Emily Sonestedt, Thorkild I. A. Sørensen, Charles Sossa Jérome, Aïcha Soumaré, Kaan Sozmen, Karen Sparrenberger, Jan A. Staessen, Maria G. Stathopoulou, Bill Stavreski, Jostein Steene-Johannessen, Peter Stehle, Aryeh D. Stein, Jochanan Stessman, Ranko Stevanović, Jutta Stieber, Doris Stöckl, Jakub Stokwiszewski, Karien Stronks, Maria Wany Strufaldi, Ramón Suárez-Medina, Chien-An Sun, Johan Sundström, Paibul Suriyawongpaisal, Rody G. Sy, René Charles Sylva, Moyses Szklo, E. Shyong Tai, Abdonas Tamosiunas, Eng Joo Tan, Mohammed Rasoul Tarawneh, Carolina B. Tarqui-Mamani, Anne Taylor, Julie Taylor, Grethe S. Tell, Tania Tello, K. R. Thankappan, Lutgarde Thijs, Betina H. Thuesen, Ulla Toft, Hanna K. Tolonen, Janne S. Tolstrup, Murat Topbas, Roman Topór-Madry, María José Tormo, Michael J. Tornaritis, Maties Torrent, Laura Torres-Collado, Pierre Traissac, Oanh T. H. Trinh, Julia Truthmann, Shoichiro Tsugane, Marshall K. Tulloch-Reid, Tomi-Pekka Tuomainen, Jaakko Tuomilehto, Anne Tybjaerg-Hansen, Christophe Tzourio, Peter Ueda, Eunice Ugel, Hanno Ulmer, Belgin Unal, Hannu M. T. Uusitalo, Gonzalo Valdivia, Damaskini Valvi, Rob M. van Dam, Yvonne T. van der Schouw, Koen Van Herck, Hoang Van Minh, Lenie van Rossem, Natasja M. Van Schoor, Irene G. M. van Valkengoed, Dirk Vanderschueren, Diego Vanuzzo, Anette Varbo, Patricia Varona-Pérez, Senthil K. Vasan, Lars Vatten, Tomas Vega, Toomas Veidebaum, Gustavo Velasquez-Melendez, Silvia J. Venero-Fernández, Giovanni Veronesi, W. M. Monique Verschuren, Cesar G. Victora, Dhanasari Vidiawati, Lucie Viet, Salvador Villalpando, Jesus Vioque, Jyrki K. Virtanen, Sophie Visvikis-Siest, Bharathi Viswanathan, Tiina Vlasoff, Peter Vollenweider, Ari Voutilainen, Alisha N. Wade, Aline Wagner, Janette Walton, Wan Mohamad Wan Bebakar, Wan Nazaimoon Wan Mohamud, Ming-Dong Wang, Ningli Wang, Qian Wang, Ya Xing Wang, Ying-Wei Wang, S. Goya Wannamethee, Niels Wedderkopp, Wenbin Wei, Peter H. Whincup, Kurt Widhalm, Indah S. Widyahening, Andrzej Wiecek, Alet H. Wijga, Rainford J. Wilks, Johann Willeit, Peter Willeit, Tom Wilsgaard, Bogdan Wojtyniak, Roy A. Wong-McClure, Andrew Wong, Tien Yin Wong, Jean Woo, Mark Woodward, Frederick C. Wu, Shouling Wu, Haiquan Xu, Liang Xu, Weili Yan, Xiaoguang Yang, Tabara Yasuharu, Xingwang Ye, Toh Peng Yeow, Panayiotis K. Yiallouros, Moein Yoosefi, Akihiro Yoshihara, San-Lin You, Novie O. Younger-Coleman, Ahmad Faudzi Yusoff, Ahmad A. Zainuddin, Seyed Rasoul Zakavi, Mohammad Reza Zali, Farhad Zamani, Sabina Zambon, Antonis Zampelas, Ko Ko Zaw, Tomasz Zdrojewski, Tajana Zeljkovic Vrkic, Zhen-Yu Zhang, Wenhua Zhao, Shiqi Zhen, Yingfeng Zheng, Bekbolat Zholdin, Baurzhan Zhussupov, Nada Zoghlami, Julio Zuñiga Cisneros, Edward W. Gregg, Majid Ezzati

**Affiliations:** 1 Imperial College London, London, UK; 2 Harvard T. H. Chan School of Public Health, Boston, MA USA; 3 University of Auckland, Auckland, New Zealand; 4 Tehran University of Medical Sciences, Tehran, Iran; 5 Middlesex University, London, UK; 6 Shahid Beheshti University of Medical Sciences, Tehran, Iran; 7 Rush University Medical Center, Chicago, IL USA; 8 Institute for Clinical Effectiveness and Health Policy, Buenos Aires, Argentina; 9 Harvard Medical School, Boston, MA USA; 10 Erasmus Medical Center Rotterdam, Rotterdam, The Netherlands; 11 World Health Organization, Geneva, Switzerland; 12 Independent researcher, Los Angeles, CA USA; 13 University of California Berkeley, Berkeley, CA USA; 14 Mahidol University, Nakhon Pathom, Thailand; 15 Charles University in Prague, Prague, Czech Republic; 16 Thomayer Hospital, Prague, Czech Republic; 17 Istituto Superiore di Sanità, Rome, Italy; 18 South African Medical Research Council, Cape Town, South Africa; 19 Seoul National University, Seoul, Republic of Korea; 20 Finnish Institute for Health and Welfare, Helsinki, Finland; 21 ICMR–National Institute of Nutrition, Hyderabad, India; 22 Pontificia Universidad Católica de Chile, Santiago, Chile; 23 ICMR–National Centre for Disease Informatics and Research, Bengaluru, India; 24 Copenhagen University Hospital, Copenhagen, Denmark; 25 Capital Medical University Beijing An Zhen Hospital, Beijing, China; 26 Bispebjerg and Frederiksberg Hospital, Copenhagen, Denmark; 27 Caja Costarricense de Seguro Social, San José, Costa Rica; 28 Qatar University, Doha, Qatar; 29 Birzeit University, Birzeit, Palestine; 30 Instituto Mexicano del Seguro Social, Mexico City, Mexico; 31 Flinders University, Adelaide, South Australia Australia; 32 Food and Nutrition Research Institute, Taguig, The Philippines; 33 Urmia University of Medical Sciences, Urmia, Iran; 34 Instituto Nacional de Ciencias Médicas y Nutricion, Mexico City, Mexico; 35 University of Amsterdam, Amsterdam, The Netherlands; 36 Steno Diabetes Center Copenhagen, Gentofte, Denmark; 37 Ministry of Health Malaysia, Kuala Lumpur, Malaysia; 38 Shahrekord University of Medical Sciences, Shahrekord, Iran; 39 University of Oslo, Oslo, Norway; 40 University of Bremen, Bremen, Germany; 41 National Center for Diabetes, Endocrinology and Genetics, Amman, Jordan; 42 Department of Internal Medicine, School of Medicine, University of Jordan, Amman, Jordan; 43 Dasman Diabetes Institute, Kuwait City, Kuwait; 44 Aldara Hospital and Medical Center, Riyadh, Saudi Arabia; 45 King Abdullah International Medical Research Center, Riyadh, Saudi Arabia; 46 Luxembourg Institute of Health, Strassen, Luxembourg; 47 World Health Organization Regional Office for the Eastern Mediterranean, Cairo, Egypt; 48 Bombay Hospital and Medical Research Centre, Mumbai, India; 49 University of Lille, Lille, France; 50 Lille University Hospital, Lille, France; 51 Western Norway University of Applied Sciences, Sogndal, Norway; 52 Norwegian School of Sport Sciences, Oslo, Norway; 53 Madras Diabetes Research Foundation, Chennai, India; 54 Zahedan University of Medical Sciences, Zahedan, Iran; 55 National Institute of Public Health, Tunis, Tunisia; 56 Institute of Public Health of the University of Porto, Porto, Portugal; 57 Norwegian Institute of Public Health, Oslo, Norway; 58 University of Massachusetts, Amherst, MA USA; 59 Abt Associates, Kathmandu, Nepal; 60 University of Iceland, Reykjavik, Iceland; 61 Federal University of Pelotas, Pelotas, Brazil; 62 University of Oulu, Oulu, Finland; 63 Oulu University Hospital, Oulu, Finland; 64 Regional Authority of Public Health, Banska Bystrica, Slovakia; 65 University of Porto Medical School, Porto, Portugal; 66 Research Institute for Endocrine Sciences, Tehran, Iran; 67 University of Science and Technology, Sana’a, Yemen; 68 Medical University of Lodz, Lodz, Poland; 69 Medical University of Gdansk, Gdansk, Poland; 70 Universidad Autónoma de Madrid/CIBERESP, Madrid, Spain; 71 University of Palermo, Palermo, Italy; 72 Pan American Health Organization, Washington, DC USA; 73 Mohammed V University de Rabat, Rabat, Morocco; 74 Dalhousie University, Halifax, Nova Scotia Canada; 75 Jordan University of Science and Technology, Irbid, Jordan; 76 Al-Farabi Kazakh National University, Almaty, Kazakhstan; 77 University of Sydney, Sydney, New South Wales Australia; 78 Christian Medical College, Vellore, India; 79 University Tunis El Manar, Tunis, Tunisia; 80 Cafam University Foundation, Bogota, Colombia; 81 Kazakh National Medical University, Almaty, Kazakhstan; 82 Universidad Peruana Cayetano Heredia, Lima, Peru; 83 Lithuanian University of Health Sciences, Kaunas, Lithuania; 84 University of São Paulo, São Paulo, Brazil; 85 Sunder Lal Jain Hospital, Delhi, India; 86 Shanghai Jiao-Tong University School of Medicine, Shanghai, China; 87 Public Health Agency of Canada, Ottawa, Ontario Canada; 88 Ufa Eye Research Institute, Ufa, Russia; 89 Nepal Health Research Council, Kathmandu, Nepal; 90 University of Southern Denmark, Copenhagen, Denmark; 91 University of Gothenburg, Gothenburg, Sweden; 92 Universidade Federal do Rio de Janeiro, Rio de Janeiro, Brazil; 93 National Institute for Public Health and the Environment, Bilthoven, The Netherlands; 94 University of Turin, Turin, Italy; 95 Nanyang Technological University, Singapore, Singapore; 96 Universidad de la República, Montevideo, Uruguay; 97 Centro de Educación Médica e Investigaciones Clínicas, Buenos Aires, Argentina; 98 IRCCS Neuromed, Pozzilli, Italy; 99 Toulouse University School of Medicine, Toulouse, France; 100 University Hospital of Varese, Varese, Italy; 101 University Hospital KU Leuven, Leuven, Belgium; 102 Ministry of Health, Victoria, Seychelles; 103 University of Lausanne, Lausanne, Switzerland; 104 Universidad Central de Venezuela, Caracas, Venezuela; 105 Bielefeld University, Bielefeld, Germany; 106 German Cancer Research Center, Heidelberg, Germany; 107 University College Copenhagen, Copenhagen, Denmark; 108 Robert Koch Institute, Berlin, Germany; 109 Universidad de La Laguna, Tenerife, Spain; 110 University of Malta, Msida, Malta; 111 Istanbul University – Cerrahpasa, Istanbul, Turkey; 112 Universidade Federal de Juiz de Fora, Juiz de Fora, Brazil; 113 Gaetano Fucito Hospital, Mercato San Severino, Italy; 114 University of Porto, Porto, Portugal; 115 Santiago de Compostela University, Santiago, Spain; 116 Council for Agricultural Research and Economics, Rome, Italy; 117 Research and Education Institute of Child Health, Nicosia, Cyprus; 118 Dr. A. Ramachandran’s Diabetes Hospital, Chennai, India; 119 University College London, London, UK; 120 Academia Sinica, Taipei, Taiwan; 121 Capital Institute of Pediatrics, Beijing, China; 122 Kailuan General Hospital, Tangshan, China; 123 Duke-NUS Medical School, Singapore, Singapore; 124 Ahvaz Jundishapur University of Medical Sciences, Ahvaz, Iran; 125 The Gertner Institute for Epidemiology and Health Policy Research, Ramat Gan, Israel; 126 Ministry of Health and Welfare, Taipei, Taiwan; 127 Murcia Health Council, Murcia, Spain; 128 Seoul National University College of Medicine, Seoul, Republic of Korea; 129 Korea Centers for Disease Control and Prevention, Cheongju-si, Republic of Korea; 130 Medical University of Silesia, Katowice, Poland; 131 Katholieke Universiteit Leuven, Leuven, Belgium; 132 Statistics Canada, Ottawa, Ontario Canada; 133 Ghent University, Ghent, Belgium; 134 Agency for Preventive and Social Medicine, Bregenz, Austria; 135 Federal University of Maranhão, São Luís, Brazil; 136 University of Southampton, Southampton, UK; 137 Institut Pasteur de Lille, Lille, France; 138 CIBEROBN, Madrid, Spain; 139 University of Debrecen, Debrecen, Hungary; 140 Universidade Federal do Rio Grande do Sul, Porto Alegre, Brazil; 141 National Council of Research, Reggio Calabria, Italy; 142 Federal University of Santa Catarina, Florianópolis, Brazil; 143 Eduardo Mondlane University, Maputo, Mozambique; 144 Geneva University Hospitals, Geneva, Switzerland; 145 National Research Centre for Preventive Medicine, Moscow, Russia; 146 University of Montreal, Montreal, Québec Canada; 147 French Public Health Agency, St Maurice, France; 148 Mediterranea Cardiocentro, Naples, Italy; 149 University of Zagreb, Zagreb, Croatia; 150 Ministry of Health and Medical Education, Tehran, Iran; 151 University of Queensland, Brisbane, Queensland Australia; 152 Universidad de Cuenca, Cuenca, Ecuador; 153 Helmholtz Zentrum München, Munich, Germany; 154 Carol Davila University of Medicine and Pharmacy, Bucharest, Romania; 155 University Hospital Düsseldorf, Düsseldorf, Germany; 156 National Institute of Cardiology, Warsaw, Poland; 157 Beth Israel Deaconess Medical Center, Boston, MA USA; 158 University of Latvia, Riga, Latvia; 159 Ministry of Health and Medical Services, Gizo, Solomon Islands; 160 Hormozgan University of Medical Sciences, Bandar Abbas, Iran; 161 University of Skövde, Skövde, Sweden; 162 National Institute of Nutrition and Food Technology, Tunis, Tunisia; 163 The University of the West Indies, Kingston, Jamaica; 164 Institut Hospital del Mar d’Investigacions Mèdiques, Barcelona, Spain; 165 University of Stellenbosch, Cape Town, South Africa; 166 University of Duisburg-Essen, Duisburg, Germany; 167 Karadeniz Technical University, Trabzon, Turkey; 168 University of Helsinki, Helsinki, Finland; 169 Mashhad University of Medical Sciences, Mashhad, Iran; 170 Rafsanjan University of Medical Sciences, Rafsanjan, Iran; 171 Queen’s University of Belfast, Belfast, UK; 172 University of Zurich, Zurich, Switzerland; 173 Tabriz University of Medical Sciences, Tabriz, Iran; 174 Fasa University of Medical Sciences, Fasa, Iran; 175 Shiraz University of Medical Sciences, Shiraz, Iran; 176 Centro de Salud Villanueva Norte, Badajoz, Spain; 177 Servicio Extremeño de Salud, Badajoz, Spain; 178 Ministry of Health, Buenos Aires, Argentina; 179 Universiti Sains Malaysia, Kelantan, Malaysia; 180 Umeå University, Umeå, Sweden; 181 Federal University of São Paulo, São Paulo, Brazil; 182 Hospital Universitario Son Espases, Palma, Spain; 183 Kindai University, Osaka-Sayama, Japan; 184 Kyoto University, Kyoto, Japan; 185 Medical University of Warsaw, Warsaw, Poland; 186 University of Catania, Catania, Italy; 187 CIBER en Epidemiología y Salud Pública, Alicante, Spain; 188 CIBER en Epidemiología y Salud Pública, Barcelona, Spain; 189 Universidade Federal de Minas Gerais, Belo Horizonte, Brazil; 190 Wageningen University, Wageningen, The Netherlands; 191 B. P. Koirala Institute of Health Sciences, Dharan, Nepal; 192 University of Insubria, Varese, Italy; 193 University of Adelaide, Adelaide, South Australia Australia; 194 Lund University, Lund, Sweden; 195 McGill University, Montreal, Québec Canada; 196 Universidad Politécnica de Madrid, Madrid, Spain; 197 St Anne’s University Hospital, Brno, Czech Republic; 198 National Institute of Public Health, Cuernavaca, Mexico; 199 Centro de Estudios en Diabetes A.C., Mexico City, Mexico; 200 Universidad Autónoma de Santo Domingo, Santo Domingo, Dominican Republic; 201 Institute for Clinical and Experimental Medicine, Prague, Czech Republic; 202 Jagiellonian University Medical College, Kraków, Poland; 203 University of Southern Denmark, Odense, Denmark; 204 National Center of Cardiovascular Diseases, Beijing, China; 205 Singapore Eye Research Institute, Singapore, Singapore; 206 Icelandic Heart Association, Kopavogur, Iceland; 207 Universidad Icesi, Cali, Colombia; 208 Eternal Heart Care Centre and Research Institute, Jaipur, India; 209 National Health Insurance Service, Wonju, Republic of Korea; 210 Prevention of Metabolic Disorders Research Center, Tehran, Iran; 211 The University of the West Indies, Cave Hill, Barbados; 212 Kermanshah University of Medical Sciences, Kermanshah, Iran; 213 University of Eastern Finland, Kuopio, Finland; 214 Kyushu University, Fukuoka, Japan; 215 University of Bergen, Bergen, Norway; 216 Tulane University, New Orleans, LA USA; 217 Chinese Center for Disease Control and Prevention, Beijing, China; 218 Joep Lange Institute, Amsterdam, The Netherlands; 219 Chronic Diseases Research Center, Tehran, Iran; 220 University of Hong Kong, Hong Kong, China; 221 The Chinese University of Hong Kong, Hong Kong, China; 222 University of Western Australia, Perth, Western Australia Australia; 223 Kingston Health Sciences Centre, Kingston, Ontario Canada; 224 Heart Institute, São Paulo, Brazil; 225 Fundación Oftalmológica de Santander, Bucaramanga, Colombia; 226 University Oran 1, Oran, Algeria; 227 Independent Public Health Specialist, Nay Pyi Taw, Myanmar; 228 Ministry of Health and Sports, Nay Pyi Taw, Myanmar; 229 CIBER en Epidemiología y Salud Pública, Murcia, Spain; 230 VU University Medical Center, Amsterdam, The Netherlands; 231 International Agency for Research on Cancer, Lyon, France; 232 American University of Beirut, Beirut, Lebanon; 233 Cairo University, Cairo, Egypt; 234 University of Zaragoza, Zaragoza, Spain; 235 National Institutes of Biomedical Innovation, Health and Nutrition, Tokyo, Japan; 236 Medical University Varna, Varna, Bulgaria; 237 The University of Tokyo, Tokyo, Japan; 238 The Hospital for Sick Children, Toronto, Ontario Canada; 239 Niigata University, Niigata, Japan; 240 Hadassah University Medical Center, Jerusalem, Israel; 241 Norwegian University of Science and Technology, Trondheim, Norway; 242 University of Melbourne, Melbourne, Victoria Australia; 243 University Hospital Centre Zagreb, Zagreb, Croatia; 244 University of Zagreb School of Medicine, Zagreb, Croatia; 245 Heart Foundation, Melbourne, Victoria Australia; 246 Guangzhou 12th Hospital, Guangzhou, China; 247 Universidad Eugenio Maria de Hostos, Santo Domingo, Dominican Republic; 248 Simon Fraser University, Burnaby, British Columbia Canada; 249 Ruprecht-Karls-University of Heidelberg, Heidelberg, Germany; 250 World Health Organization Country Office, Delhi, India; 251 Guilan University of Medical Sciences, Rasht, Iran; 252 University of Opole, Opole, Poland; 253 University of Crete, Heraklion, Greece; 254 Universiti Kebangsaan Malaysia, Kuala Lumpur, Malaysia; 255 Maharajgunj Medical Campus, Kathmandu, Nepal; 256 Johns Hopkins Bloomberg School of Public Health, Baltimore, MD USA; 257 National Institute of Epidemiology, Chennai, India; 258 University of Münster, Münster, Germany; 259 Research Institute for Primordial Prevention of Non-communicable Disease, Isfahan, Iran; 260 Kyrgyz State Medical Academy, Bishkek, Kyrgyzstan; 261 Research Institute of Child Nutrition, Dortmund, Germany; 262 Mazandaran University of Medical Sciences, Sari, Iran; 263 Hypertension Research Center, Isfahan, Iran; 264 Medical University of Innsbruck, Innsbruck, Austria; 265 Muhimbili University of Health and Allied Sciences, Dar es Salaam, Tanzania; 266 Yonsei University College of Medicine, Seoul, Republic of Korea; 267 National Cancer Center, Goyang-si, Republic of Korea; 268 University of Pennsylvania, Philadelphia, PA USA; 269 University of Vienna, Vienna, Austria; 270 Oulu Deaconess Institute Foundation, Oulu, Finland; 271 Tartu University Clinics, Tartu, Estonia; 272 Kansai Medical University, Hirakata, Japan; 273 Ministry of Health and Quality of Life, Port Louis, Mauritius; 274 University Hospital Ulm, Ulm, Germany; 275 University of Groningen, Groningen, The Netherlands; 276 University of Jyväskylä, Jyväskylä, Finland; 277 African Population and Health Research Center, Nairobi, Kenya; 278 Higher Institute of Health Sciences of Settat, Settat, Morocco; 279 Ministry of Health, Algiers, Algeria; 280 Harokopio University, Athens, Greece; 281 Sahlgrenska Academy, Gothenburg, Sweden; 282 Endocrinology and Metabolism Research Center, Tehran, Iran; 283 University of Public Health, Yangon, Myanmar; 284 National University of Singapore, Singapore, Singapore; 285 Tampere University Hospital, Tampere, Finland; 286 Tampere University, Tampere, Finland; 287 University of Cape Town, Cape Town, South Africa; 288 West Virginia University, Morgantown, WV USA; 289 Oswaldo Cruz Foundation Rene Rachou Research Institute, Belo Horizonte, Brazil; 290 National Taiwan University, Taipei, Taiwan; 291 University of Chinese Academy of Sciences, Shanghai, China; 292 Uppsala University, Uppsala, Sweden; 293 National Institute for Health Development, Tallinn, Estonia; 294 Universidad San Martín de Porres, Lima, Peru; 295 Consejería de Sanidad Junta de Castilla y León, Valladolid, Spain; 296 Norrbotten County Council, Luleå, Sweden; 297 Peking University, Beijing, China; 298 Universidade Federal de Ouro Preto, Ouro Preto, Brazil; 299 University of Coimbra, Coimbra, Portugal; 300 The Jikei University School of Medicine, Tokyo, Japan; 301 Institute of Neuroscience of the National Research Council, Padua, Italy; 302 Baker Heart and Diabetes Institute, Melbourne, Victoria Australia; 303 Agricultural University of Athens, Athens, Greece; 304 French National Research Institute for Sustainable Development, Montpellier, France; 305 Hospital Israelita Albert Einstein, São Paulo, Brazil; 306 University of Otago, Dunedin, New Zealand; 307 University of Padua, Padua, Italy; 308 Lausanne University Hospital, Lausanne, Switzerland; 309 Emory University, Atlanta, GA USA; 310 Universidade Estadual do Centro-Oeste, Guarapuava, Brazil; 311 UiT The Arctic University of Norway, Tromsø, Norway; 312 Cape Peninsula University of Technology, Cape Town, South Africa; 313 Brown University, Providence, RI USA; 314 University of Edinburgh, Edinburgh, UK; 315 University College Dublin, Dublin, Ireland; 316 Shahid Sadoughi University of Medical Sciences, Yazd, Iran; 317 Institut National de la Santé et de la Recherche Médicale, Lille, France; 318 ICMR–National Institute of Medical Statistics, New Delhi, India; 319 Lusófona University, Lisbon, Portugal; 320 Women’s Social and Health Studies Foundation, Trivandrum, India; 321 Università degli Studi di Firenze, Florence, Italy; 322 Ain Shams University, Cairo, Egypt; 323 Isfahan Cardiovascular Research Center, Isfahan, Iran; 324 University of Strasbourg, Strasbourg, France; 325 Strasbourg University Hospital, Strasbourg, France; 326 University of Pécs, Pécs, Hungary; 327 Mulago Hospital, Kampala, Uganda; 328 University of Medical Sciences of Cienfuegos, Cienfuegos, Cuba; 329 Royal College of Surgeons in Ireland Dublin, Dublin, Ireland; 330 La Trobe University, Melbourne, Victoria Australia; 331 International Institute of Molecular and Cell Biology, Warsaw, Poland; 332 Instituto Conmemorativo Gorgas de Estudios de la Salud, Panama City, Panama; 333 World Health Organization Country Office, Lilongwe, Malawi; 334 University of Brescia, Brescia, Italy; 335 Bushehr University of Medical Sciences, Bushehr, Iran; 336 Ulm University, Ulm, Germany; 337 Kobe University, Kobe, Japan; 338 Suraj Eye Institute, Nagpur, India; 339 University Medicine of Greifswald, Greifswald, Germany; 340 University of Medicine and Pharmacy at Ho Chi Minh City, Ho Chi Minh City, Vietnam; 341 Hanoi Medical University, Hanoi, Vietnam; 342 Miami Veterans Affairs Healthcare System, Miami, FL USA; 343 University of Turku, Turku, Finland; 344 Eastern Mediterranean Public Health Network, Amman, Jordan; 345 University of Manchester, Manchester, UK; 346 Japan Wildlife Research Center, Tokyo, Japan; 347 Istanbul University, Istanbul, Turkey; 348 Ministry of Health, Bandar Seri Begawan, Brunei; 349 University of Madeira, Funchal, Portugal; 350 MRC Lifecourse Epidemiology Unit, Southampton, UK; 351 University of Novi Sad, Novi Sad, Serbia; 352 Kwame Nkrumah University of Science and Technology, Kumasi, Ghana; 353 Institute for Social and Preventive Medicine, Ottawa, Ontario Canada; 354 IRCCS Ente Ospedaliero Specializzato in Gastroenterologia S. de Bellis, Bari, Italy; 355 Jivandeep Hospital, Anand, India; 356 South African Medical Research Council, Durban, South Africa; 357 Vietnam National Heart Institute, Hanoi, Vietnam; 358 Clínica de Medicina Avanzada Dr. Abel González, Santo Domingo, Dominican Republic; 359 Leibniz Institute for Prevention Research and Epidemiology – BIPS, Bremen, Germany; 360 University of Sarajevo, Sarajevo, Bosnia and Herzegovina; 361 Cardiovascular Prevention Centre, Udine, Italy; 362 Public Health Agency of Catalonia, Barcelona, Spain; 363 Observatorio de Salud Pública de Santander, Bucaramanga, Colombia; 364 Ardabil University of Medical Sciences, Ardabil, Iran; 365 Alborz University of Medical Sciences, Karaj, Iran; 366 Ministry of Health, Hanoi, Vietnam; 367 India Diabetes Research Foundation, Chennai, India; 368 Institut Universitari d’Investigació en Atenció Primària Jordi Gol, Girona, Spain; 369 Universiti Putra Malaysia, Serdang, Malaysia; 370 University of Malaya, Kuala Lumpur, Malaysia; 371 University of Valencia, Valencia, Spain; 372 University of the Philippines, Manila, The Philippines; 373 Minas Gerais State Secretariat for Health, Belo Horizonte, Brazil; 374 CS S. Agustín Ibsalut, Palma, Spain; 375 Amsterdam Institute for Global Health and Development, Amsterdam, The Netherlands; 376 Canarian Health Service, Tenerife, Spain; 377 Universidad Industrial de Santander, Bucaramanga, Colombia; 378 Associazione Calabrese di Epatologia, Reggio Calabria, Italy; 379 Sahlgrenska University Hospital, Gothenburg, Sweden; 380 Toulouse University Hospital, Toulouse, France; 381 Institute of Food Sciences of the National Research Council, Avellino, Italy; 382 Sitaram Bhartia Institute of Science and Research, New Delhi, India; 383 Faculty of Medicine of Tunis, Tunis, Tunisia; 384 National Institute of Health, Lima, Peru; 385 Catalan Department of Health, Barcelona, Spain; 386 Universidade de Lisboa, Lisbon, Portugal; 387 South Karelia Social and Health Care District, Lappeenranta, Finland; 388 Cardiovascular Research Institute, Isfahan, Iran; 389 National Cancer Center, Tokyo, Japan; 390 University of São Paulo Clinics Hospital, São Paulo, Brazil; 391 Hospital Italiano de Buenos Aires, Buenos Aires, Argentina; 392 Center for Oral Health Services and Research Mid-Norway, Trondheim, Norway; 393 King’s College London, London, UK; 394 National Center for Global Health and Medicine, Tokyo, Japan; 395 Sungkyunkwan University, Seoul, Republic of Korea; 396 Finnish Institute of Occupational Health, Helsinki, Finland; 397 St Vincent’s Hospital, Sydney, New South Wales Australia; 398 University of New South Wales, Sydney, New South Wales Australia; 399 Karolinska Institutet, Stockholm, Sweden; 400 Research Centre for Prevention and Health, Glostrup, Denmark; 401 London School of Hygiene & Tropical Medicine, London, UK; 402 Diponegoro University, Semarang, Indonesia; 403 University of Bari, Bari, Italy; 404 University of Copenhagen, Copenhagen, Denmark; 405 Institut Régional de Santé Publique, Ouidah, Benin; 406 University of Bordeaux, Bordeaux, France; 407 Izmir Katip Çelebi University, Izmir, Turkey; 408 University of Leuven, Leuven, Belgium; 409 Institut National de la Santé et de la Recherche Médicale, Nancy, France; 410 Bonn University, Bonn, Germany; 411 Croatian Institute of Public Health, Zagreb, Croatia; 412 National Institute of Public Health–National Institute of Hygiene, Warsaw, Poland; 413 National Institute of Hygiene, Epidemiology and Microbiology, Havana, Cuba; 414 Fu Jen Catholic University, Taipei, Taiwan; 415 National Statistic Office of Cabo Verde, Praia, Cabo Verde; 416 Ministry of Health, Amman, Jordan; 417 Central University of Kerala, Kasaragod, India; 418 Health Service of Murcia, Murcia, Spain; 419 Institut d’Investigacio Sanitaria Illes Balears, Menorca, Spain; 420 Universidad Centro-Occidental Lisandro Alvarado, Barquisimeto, Venezuela; 421 Dokuz Eylul University, Izmir, Turkey; 422 University of Tampere Tays Eye Center, Tampere, Finland; 423 Icahn School of Medicine at Mount Sinai, New York City, NY USA; 424 Utrecht University, Utrecht, The Netherlands; 425 Hanoi University of Public Health, Hanoi, Vietnam; 426 University Medical Center Utrecht, Utrecht, The Netherlands; 427 Universitas Indonesia, Jakarta, Indonesia; 428 Instituto de Investigación Sanitaria y Biomédica de Alicante, Alicante, Spain; 429 North Karelian Center for Public Health, Joensuu, Finland; 430 University of the Witwatersrand, Johannesburg, South Africa; 431 Cork Institute of Technology, Cork, Ireland; 432 Institute for Medical Research, Kuala Lumpur, Malaysia; 433 Health Canada, Ottawa, Ontario Canada; 434 Beijing Institute of Ophthalmology, Beijing, China; 435 Xinjiang Medical University, Urumqi, China; 436 Capital Medical University, Beijing, China; 437 St George’s, University of London, London, UK; 438 Medical University of Vienna, Vienna, Austria; 439 University of Oxford, Oxford, UK; 440 Institute of Food and Nutrition Development of Ministry of Agriculture and Rural Affairs, Beijing, China; 441 Children’s Hospital of Fudan University, Shanghai, China; 442 Penang Medical College, Penang, Malaysia; 443 University of Cyprus, Nicosia, Cyprus; 444 Iran University of Medical Sciences, Tehran, Iran; 445 Jiangsu Provincial Center for Disease Control and Prevention, Nanjing, China; 446 Sun Yat-sen University, Guangzhou, China; 447 West Kazakhstan State Medical University, Aktobe, Kazakhstan; 448 University of Ghana, Accra, Ghana

**Keywords:** Cardiovascular diseases, Risk factors

## Abstract

High blood cholesterol is typically considered a feature of wealthy western countries^1,2^. However, dietary and behavioural determinants of blood cholesterol are changing rapidly throughout the world^3^ and countries are using lipid-lowering medications at varying rates. These changes can have distinct effects on the levels of high-density lipoprotein (HDL) cholesterol and non-HDL cholesterol, which have different effects on human health^4,5^. However, the trends of HDL and non-HDL cholesterol levels over time have not been previously reported in a global analysis. Here we pooled 1,127 population-based studies that measured blood lipids in 102.6 million individuals aged 18 years and older to estimate trends from 1980 to 2018 in mean total, non-HDL and HDL cholesterol levels for 200 countries. Globally, there was little change in total or non-HDL cholesterol from 1980 to 2018. This was a net effect of increases in low- and middle-income countries, especially in east and southeast Asia, and decreases in high-income western countries, especially those in northwestern Europe, and in central and eastern Europe. As a result, countries with the highest level of non-HDL cholesterol—which is a marker of cardiovascular risk—changed from those in western Europe such as Belgium, Finland, Greenland, Iceland, Norway, Sweden, Switzerland and Malta in 1980 to those in Asia and the Pacific, such as Tokelau, Malaysia, The Philippines and Thailand. In 2017, high non-HDL cholesterol was responsible for an estimated 3.9 million (95% credible interval 3.7 million–4.2 million) worldwide deaths, half of which occurred in east, southeast and south Asia. The global repositioning of lipid-related risk, with non-optimal cholesterol shifting from a distinct feature of high-income countries in northwestern Europe, north America and Australasia to one that affects countries in east and southeast Asia and Oceania should motivate the use of population-based policies and personal interventions to improve nutrition and enhance access to treatment throughout the world.

## Abstract (web summary)

From 1980 to 2018, the levels of total and non-high-density lipoprotein cholesterol increased in low- and middle-income countries, especially in east and southeast Asia, and decreased in high-income western countries, especially those in northwestern Europe, and in central and eastern Europe.

## Main

Blood cholesterol is one of the most important risk factors for ischaemic heart disease (IHD) and ischaemic stroke^4–6^. Consistent and comparable information on cholesterol levels and trends in different countries can help to benchmark national performance in addressing non-optimal cholesterol, investigate the reasons behind differential trends and identify countries in which interventions are needed the most.

A previous global analysis^7^ reported trends in total cholesterol from 1980 to 2008, but did not analyse important lipid fractions—including HDL and non-HDL cholesterol—that are key to understanding the cardiovascular disease risk associated with non-optimal cholesterol. Dietary and behavioural determinants of cholesterol have changed throughout the world in the past decades, including a worldwide rise in adiposity^8,9^, divergent global trends in alcohol use^10^, a rise in the intake of animal-source foods in middle-income countries (especially in east Asia)^3,11^, and a replacement of saturated fats and trans fats with unsaturated fats in some high-income countries^3,11,12^. There is also considerable variation in how much different countries have adopted lipid-lowering medications^13^. These changes are likely to have influenced cholesterol levels substantially in the decade since the last estimates were made. Furthermore, HDL and non-HDL cholesterol, which have opposite associations with cardiovascular diseases^4,5^, respond differently to diet and treatment, and may therefore have different geographical patterns and trends over time^14^. Information on these major lipid fractions, which were not included in the previous global estimates, is essential for priority setting and intervention choice.

**Extended Data Fig. 1:**
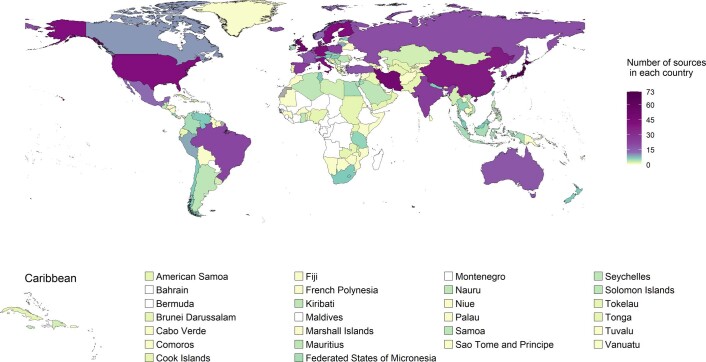
Number of data sources by country. The colour indicates the number of data sources for each country used in the analysis. Countries and territories that were not included in the analysis are coloured in grey.

**Extended Data Fig. 2:**
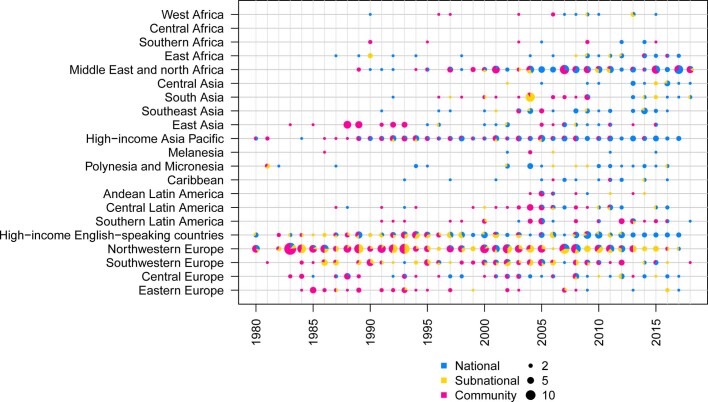
Number of data sources by region and year. The size of each circle shows the number of data sources for each region and year, and the colours indicate the relative size of national, subnational and community data sources.

Here we pooled 1,127 population-based studies that measured blood lipids in 102.6 million individuals aged 18 years and older (Extended Data Figs. 1, 2 and Supplementary Table 1) and used a Bayesian hierarchical model to estimate trends from 1980 to 2018 in mean total, non-HDL and HDL cholesterol levels for 200 countries. We also estimated the number of deaths caused by IHD and ischaemic stroke that were attributable to high levels of non-HDL cholesterol using information on its hazards from epidemiological studies.

## Trends in total cholesterol

**Fig. 1:**
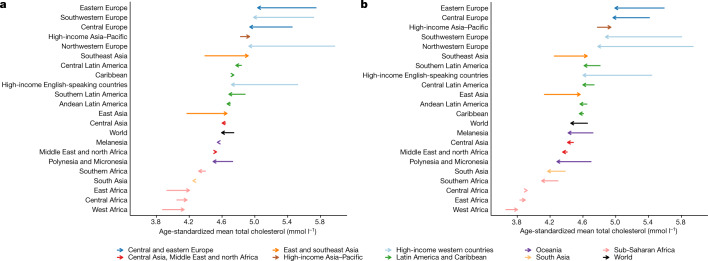
Change in age-standardized mean total cholesterol between 1980 and 2018 by region for women and men. **a**, Age-standardized mean total cholesterol in women. **b**, Age-standardized mean total cholesterol in men. The start of the arrow shows the level in 1980 and the head indicates the level in 2018. See Extended Data Fig. 3 for age-standardized mean HDL cholesterol. One mmol l^−1^ is equivalent to 38.61 mg dl^−1^.

In 2018, global age-standardized mean total cholesterol was 4.6 mmol l^−1^ (95% credible interval, 4.5–4.7) for women and 4.5 mmol l^−1^ (4.3–4.6) for men. Global age-standardized mean total cholesterol changed little over these nearly four decades, decreasing by 0.03 mmol l^−1^ per decade (−0.02–0.08) in women and 0.05 mmol l^−1^ per decade (0.00–0.11) in men (posterior probability of the observed declines being true declines = 0.90 for women and 0.98 for men) (Fig. 1). Regionally, total cholesterol decreased the most in high-income western regions and in central and eastern Europe. The decrease was the largest (around 0.3 mmol l^−1^ per decade; posterior probability >0.9999) in northwestern Europe, where mean total cholesterol levels had been the highest in 1980. The decrease in total cholesterol in high-income western regions and central and eastern Europe was largely due to a decline in non-HDL cholesterol (Extended Data Fig. 4), which among women was offset partly by an increase in mean HDL cholesterol levels. Mean total cholesterol changed little in most of the other regions, with the notable exception of east and southeast Asia, where it increased by more than 0.1 mmol l^−1^ per decade in both women and men (posterior probability ≥0.95). The increase in east and southeast Asia was largely due to an increase in non-HDL cholesterol.

## Trends in non-HDL and HDL cholesterol

In 2018, global age-standardized mean non-HDL cholesterol was 3.3 mmol l^−1^ (3.2–3.4) for women and 3.3 mmol l^−1^ (3.3–3.4) for men; global age-standardized mean HDL cholesterol was 1.3 mmol l^−1^ (1.2–1.3) for women and 1.1 mmol l^−1^ (1.1–1.2) for men. Global age-standardized mean non-HDL cholesterol remained almost unchanged from 1980 to 2018, decreasing by only 0.02 mmol l^−1^ per decade (−0.02–0.06; posterior probability = 0.80) in women and 0.01 mmol l^−1^ per decade (−0.03–0.06; posterior probability = 0.72) in men. Global age-standardized mean HDL cholesterol remained unchanged for women and decreased slightly for men (by 0.02 mmol l^−1^ per decade, posterior probability = 0.91).

**Fig. 2:**
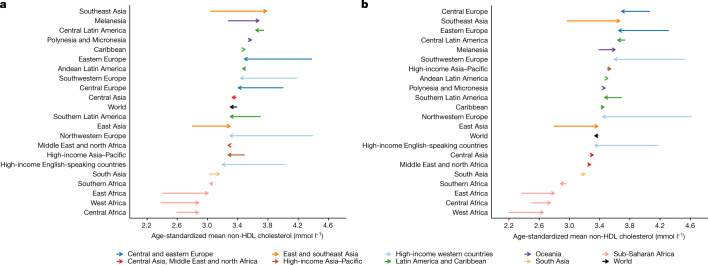
Change in age-standardized mean non-HDL cholesterol between 1980 and 2018 by region for women and men. **a**, Age-standardized mean non-HDL cholesterol in women. **b**, Age-standardized mean non-HDL cholesterol in men. The start of the arrow shows the level in 1980 and the head indicates the level in 2018. See Extended Data Fig. 3 for age-standardized mean HDL cholesterol. One mmol l^−1^ is equivalent to 38.61 mg dl^−1^.

**Extended Data Fig. 3:**
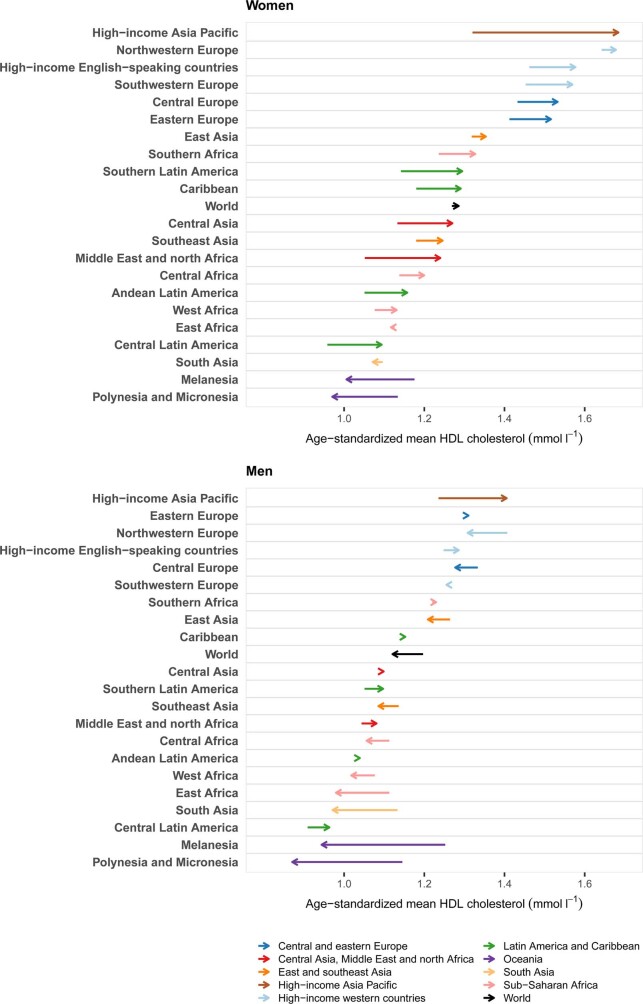
Change in age-standardized mean HDL cholesterol between 1980 and 2018 by region for women and men. The start of the arrow shows the level in 1980 and the head shows the level in 2018. One mmol l^−1^ is equivalent to 38.61 mg dl^−1^.

**Extended Data Fig. 4:**
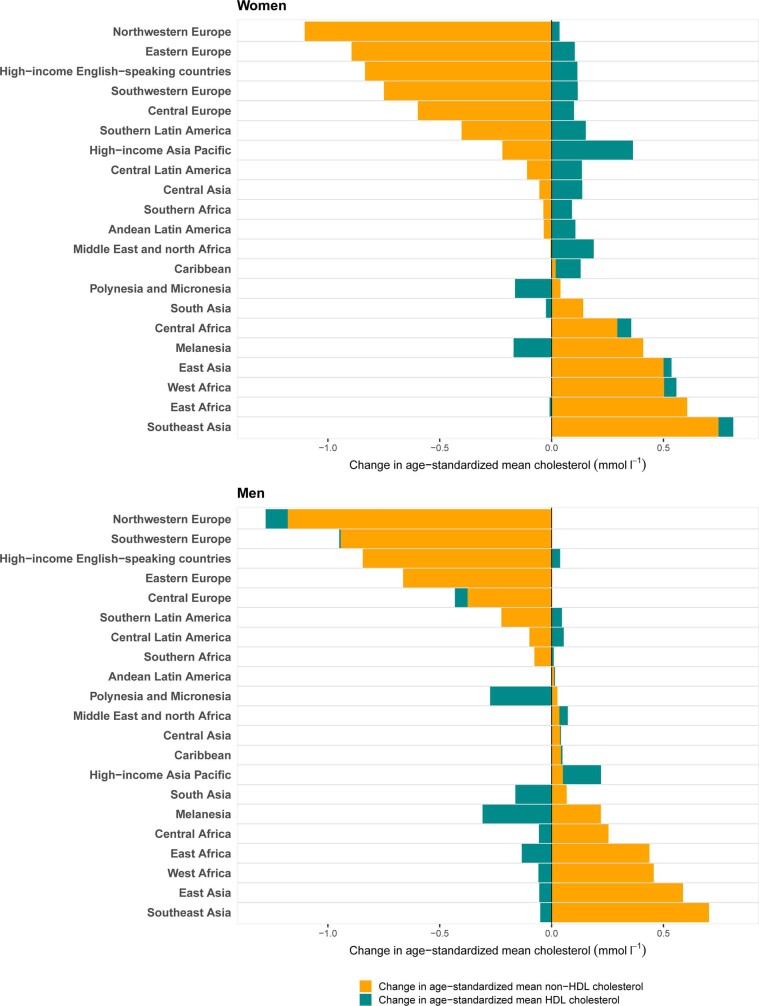
Change in age-standardized mean HDL and non-HDL cholesterol between 1980 and 2018 by region for women and men. One mmol l^−1^ is equivalent to 38.61 mg dl^−1^.

Regionally, non-HDL cholesterol decreased substantially in high-income western regions and central and eastern Europe. The largest decrease occurred in northwestern Europe (>0.3 mmol l^−1^ per decade; posterior probability >0.9999) (Fig. 2). By contrast, it increased in east and southeast Asia, parts of sub-Saharan Africa and Melanesia. The increase was the largest in southeast Asia, increasing by approximately 0.2 mmol l^−1^ per decade (posterior probability >0.9999). Mean HDL cholesterol increased in the high-income Asia–Pacific region, by as much as 0.1 mmol l^−1^ per decade in women (posterior probability >0.9999) but decreased in Melanesia, Polynesia and Micronesia (Extended Data Fig. 3).

**Fig. 3:**
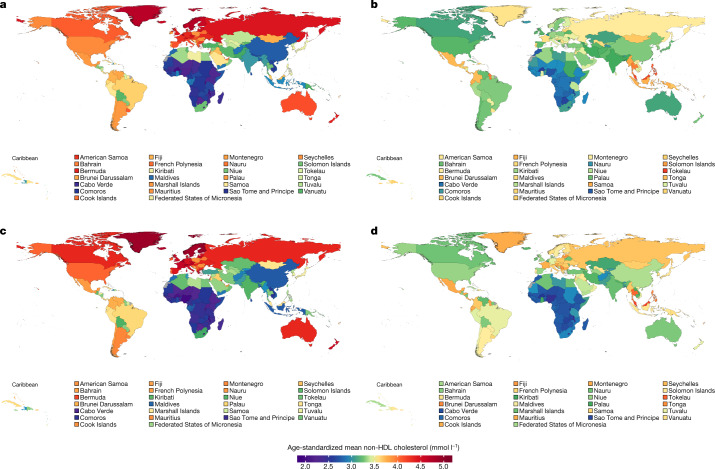
Age-standardized mean non-HDL cholesterol by country in 1980 and 2018 for women and men. **a**, Age-standardized mean non-HDL cholesterol in women in 1980. **b**, Age-standardized mean non-HDL cholesterol in women in 2018. **c**, Age-standardized mean non-HDL cholesterol in men in 1980. **d**, Age-standardized mean non-HDL cholesterol in men in 2018. See Extended Data Fig. 5 for age-standardized mean total cholesterol and Extended Data Fig. 6 for age-standardized mean HDL cholesterol. One mmol l^−1^ is equivalent to 38.61 mg dl^−1^.

**Fig. 4:**
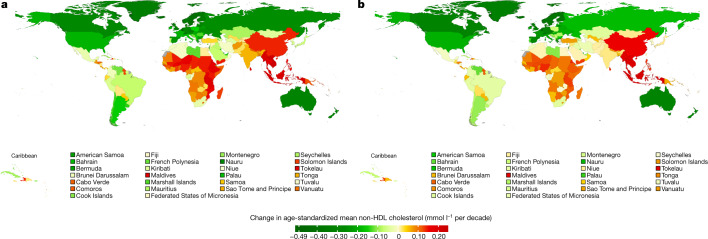
Change in age-standardized mean non-HDL cholesterol per decade by country for women and men. **a**, Change per decade in age-standardized mean non-HDL cholesterol in women. **b**, Change per decade in age-standardized mean non-HDL cholesterol in men. See Extended Data Fig. 7 for change per decade in age-standardized mean total cholesterol and Extended Data Fig. 8 for change per decade in age-standardized mean HDL cholesterol. One mmol l^−1^ is equivalent to 38.61 mg dl^−1^.

**Extended Data Fig. 6:**
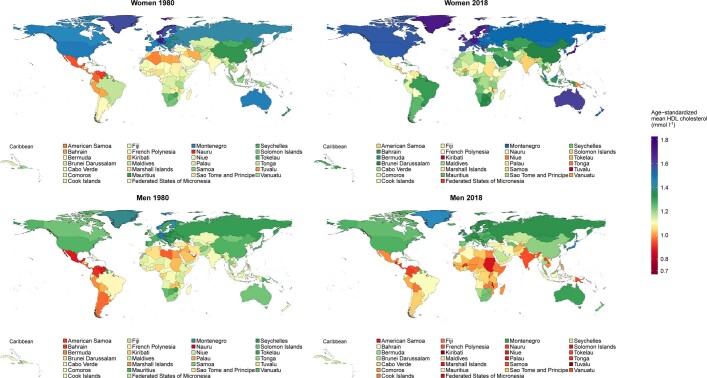
Age-standardized mean HDL cholesterol by country in 1980 and 2018 for women and men. One mmol l^−1^ is equivalent to 38.61 mg dl^−1^.

Belgium, Finland, Greenland, Iceland, Norway, Sweden, Switzerland and Malta had some of the highest non-HDL cholesterol levels in 1980 (>4.5 mmol l^−1^ in women and >4.7 mmol l^−1^ in men) but experienced some of the largest declines (Figs. 3, 4). At the extreme, mean non-HDL cholesterol declined by around 0.45 mmol l^−1^ per decade or more in Belgian and Icelandic women and men, changing their ranks from being in the top 10 countries in terms of non-HDL cholesterol in 1980 to being ranked in the lower half of the countries in 2018—below countries in southwestern Europe such as France and Italy. The largest increases were found in east Asian countries (for example, China) and southeast Asian countries (for example, Indonesia, Thailand, Malaysia, Cambodia and Lao PDR). In these countries, age-standardized mean non-HDL cholesterol increased by as much as 0.23 mmol l^−1^ per decade. As a result of these opposite trends, countries with the highest age-standardized mean non-HDL cholesterol levels in 2018 were all outside northwestern Europe: Tokelau, Malaysia, The Philippines and Thailand, all of which had mean non-HDL cholesterol around or above 4 mmol l^−1^. China, which had one of the lowest mean non-HDL cholesterol levels in 1980, reached or surpassed non-HDL cholesterol levels of many high-income western countries in 2018. Sub-Saharan African countries had the lowest mean non-HDL cholesterol in 2018, as low as 2.6 mmol l^−1^ in some countries, as they had in 1980. Not only did high-income countries benefit from decreasing non-HDL cholesterol levels, they had higher mean HDL cholesterol than low- and middle-income countries (Extended Data Fig. 6).

## Deaths attributable to non-optimal cholesterol

**Fig. 5:**
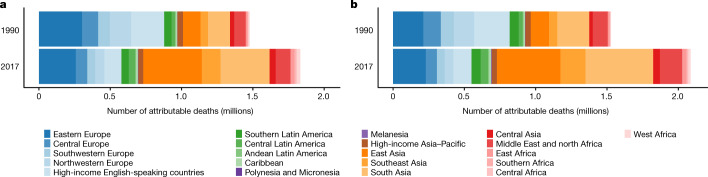
Deaths from IHD and ischaemic stroke attributable to high non-HDL cholesterol by region in 1990 and 2017 for women and men. **a**, Deaths in women attributable to high non-HDL cholesterol. **b**, Deaths in men attributable to high non-HDL cholesterol.

In 2017, high non-HDL cholesterol was responsible for an estimated 3.9 million (3.7–4.2 million) worldwide deaths from IHD and ischaemic stroke (Fig. 5), accounting for a third of deaths from these causes. From 1990 to 2017, the number of deaths caused by IHD and ischaemic stroke that were attributable to high non-HDL cholesterol increased by around 910,000 globally. This increase was a net result of a large decrease in western countries, from 950,000 (890,000–990,000) to 480,000 (430,000–530,000), and a large increase throughout Asia. In particular, the number of deaths attributable to high non-HDL cholesterol more than tripled in east Asia, from 250,000 (230,000–270,000) to 860,000 (770,000–940,000), and more than doubled in southeast Asia, from 110,000 (100,000–120,000) to 310,000 (290,000–330,000). As a result, by 2017 east, southeast and south Asia accounted for half of all deaths attributable to high non-HDL cholesterol, compared with a quarter in 1990.

## Implications

Our results show that over the past nearly four decades, there has been a major global repositioning of lipid-related risk, with non-optimal cholesterol patterns shifting from being a distinct feature of high-income countries in northwestern Europe, north America and Australasia to one that affects middle-income countries in east and southeast Asia, as well as some countries in Oceania and central Latin America. This transition is especially noticeable for non-HDL cholesterol, which had not been quantified previously in a global analysis. This global repositioning has occurred as a consequence of opposing trends in high-income western countries and in Asia, which has led to some Asian countries having the highest worldwide non-HDL cholesterol levels in 2018.

The decrease in non-HDL cholesterol in western countries started in the 1980s, before statins were widely used^15,16^. This indicates that changes in diet, especially the replacement of saturated with unsaturated fats^3,17–21^ and reduction in trans fats^12,17,22^, are major contributors to this decline. Nonetheless, the increased use of statins from the late 1990s onwards^15,16^, may explain up to one half of the decrease in those countries in which statins are widely used^19,23,24^. In contrast to high-income western countries, the consumption of animal-source foods, refined carbohydrates and palm oil has increased substantially in east and southeast Asia^3,25,26^, where statin use remains low^13,27^. For example, the Pearson correlation coefficient between the change in non-HDL cholesterol and the change in a multi-dimensional score of animal-source foods and sugar^3^ was 0.69 for women and 0.67 for men using data from high-income western countries and countries in east and southeast Asia, the two regions that experienced the largest decrease and increase, respectively, in non-HDL cholesterol levels. Finally, changes in diet, especially a decrease in carbohydrate and an increase in fat intake^28–31^, may have contributed to the large increase in HDL cholesterol observed in the high-income Asia–Pacific region, where there was little increase in overweight and obesity relative to other regions^8,9^. By contrast, the large increase in diabetes^32^ and adiposity^8^ in Oceania may have contributed to the decrease in HDL cholesterol in this region. The Pearson correlation coefficient between the change in HDL cholesterol and the change in body-mass index^8^ was −0.87 for women and −0.69 for men using countries in the high-income Asia–Pacific region and Oceania, the two regions that had the largest increase and decrease, respectively, in HDL cholesterol; the Pearson correlation coefficient for the change in HDL cholesterol and change in diabetes prevalence^32^ was −0.84 for women and −0.69 for men. In the same regions, the Pearson correlation coefficient between the change in non-HDL cholesterol and the change in body-mass index^8^ was 0.77 for women and 0.62 for men; for the change in non-HDL cholesterol and the change in diabetes prevalence^32^, the Pearson correlation coefficient was 0.54 for women and 0.40 for men.

Although it has previously been documented that the prevalence of adiposity^8,9^, diabetes^32^ and high blood pressure^33^ is now higher in low- and middle-income countries than in high-income countries, higher cholesterol is commonly considered to be a feature of affluent western nations^1,2^. We show that, when focusing on non-HDL cholesterol, middle-income countries have emerged as the new global epicentre of non-optimal cholesterol as they did for other major cardiovascular disease risk factors, indicating that there is no such a thing as a western risk factor. At the same time, the populations of high-income countries would also benefit from further lowering non-HDL cholesterol. Therefore, population-based policies and personal interventions to improve nutrition and enhance treatment are now needed in all countries, especially as a part of the movement towards universal health coverage.

## Methods

**Extended Data Fig. 9:**
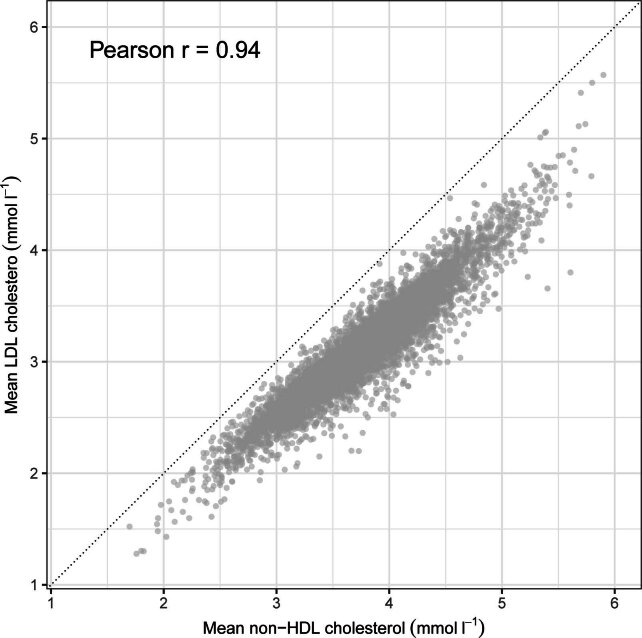
The association between mean LDL and non-HDL cholesterol in studies that measured lipids in a laboratory that had data for both variables. Each data point is one study–age–sex group (*n* = 6,864). One mmol l^−1^ is equivalent to 38.61 mg dl^−1^.

Our aim was to estimate trends in mean total, HDL and non-HDL cholesterol for 200 countries and territories (Supplementary Table 2). We used non-HDL cholesterol rather than low-density lipoprotein (LDL) cholesterol because most studies in our analysis had measured total cholesterol and HDL cholesterol, from which non-HDL cholesterol can be calculated through subtraction. By contrast, LDL cholesterol was directly measured in only around 14% of studies. When LDL cholesterol is not directly measured, its calculation requires data on triglycerides, which were available in approximately 64% of the studies. Furthermore, the most-commonly used estimation method—that is, the Friedewald equation—can be inaccurate, particularly at high levels of triglycerides^34^. Non-HDL and LDL cholesterol were highly correlated (Pearson correlation coefficient = 0.94) in studies with data on both variables (Extended Data Fig. 9), because LDL cholesterol constitutes most of non-HDL cholesterol. Furthermore, non-HDL cholesterol predicts IHD risk at least as well as LDL cholesterol^5,35^, and can be measured at a lower cost than LDL cholesterol, which is relevant for how widely it can be used in low- and middle-income countries. Although non-HDL cholesterol is now commonly used in clinical guidelines^36–38^, LDL cholesterol continues to be a key target for treatment^36,37^, possibly because the interpretation of non-HDL cholesterol is more complex than LDL cholesterol alone. Specifically, an increase in non-HDL cholesterol could be due to the increase in LDL cholesterol or very-low-density lipoprotein cholesterol^39^. Furthermore, there is some evidence that triglyceride levels are high in Asian populations, compared to levels seen in high-income western countries^40^. Therefore, data on non-HDL cholesterol can motivate dietary interventions to both reduce LDL cholesterol (for example, reducing saturated and trans fat intake) and triglyceride levels (for example, reducing refined carbohydrates and increasing omega-3 fatty acids) as well as treatments that lower LDL cholesterol (statins), alongside those that lower triglycerides (for example, fibrates).

### Data sources

We used a database of population-based data on cardiometabolic risk factors collated by the NCD Risk Factor Collaboration (NCD-RisC), a worldwide network of health researchers and practitioners that systematically monitors the worldwide trends and variations in non-communicable disease (NCD) risk factors. The database was collated through multiple routes for identifying and accessing data. We accessed publicly available population-based multi-country and national measurement surveys (for example, Demographic and Health Surveys and surveys identified through the Inter-University Consortium for Political and Social Research and European Health Interview & Health Examination Surveys Database). We requested, via the World Health Organization (WHO) and its regional and country offices, from ministries of health and other national health and statistical agencies to identify and access population-based surveys. Requests were also sent via the World Heart Federation to its national partners. We made a similar request to the co-authors of an earlier pooled analysis of cardiometabolic risk factors^7,41–43^, and invited the co-authors of the analysis to reanalyse data from their studies and join NCD-RisC. Finally, to identify major sources that were not accessed through the above routes, we searched and reviewed published studies as described in the Supplementary Information and invited all eligible studies to join NCD-RisC.

For each data source, we recorded the available information about the study population, start year and duration of measurement, sampling approach and measurement methods. The information about study population was used to establish that each data source was population-based, and to assess whether it covered the whole country, multiple subnational regions or one or a small number of communities, and whether it was rural, urban or combined.

We carefully checked all data sources in terms of how they met our inclusion and exclusion criteria listed below. We identified duplicate data sources by comparing studies from the same country and year. Additionally, all NCD-RisC members are asked periodically to review the list of sources from their country, to suggest additional sources not in the database, and to verify that the included data meet the inclusion criteria listed below and are not duplicates. The NCD-RisC database is continuously updated through the above routes and through regular contact with NCD-RisC members.

Anonymized individual record data from sources included in NCD-RisC were reanalysed according to a common protocol. Within each survey, we included participants aged 18 years and older who were not pregnant. We removed participants with implausible total cholesterol levels (defined as total cholesterol levels of <1.75 mmol l^−1^ or >20 mmol l^−1^, or total cholesterol values that were lower than HDL cholesterol values) (<0.05% of all participants with total cholesterol measurements) or HDL cholesterol levels (defined as HDL cholesterol levels of <0.4 mmol l^−1^ or >5 mmol l^−1^, or total cholesterol values that were lower than HDL cholesterol values) (<0.15% of all participants with HDL cholesterol measurements). When data on LDL cholesterol were also available, we removed individuals for whom the sum of LDL and HDL cholesterol level surpassed total cholesterol level by more than is plausible based on the limits to errors in their measurement (following the CDC Cholesterol Reference Method Laboratory Network (CRMLN) standards, these errors were set at 8.9% for total cholesterol, 13% for HDL cholesterol and 12% for LDL cholesterol) (<0.06% of all participants with total cholesterol and HDL cholesterol measurements)^44–46^.

We calculated mean total cholesterol, mean HDL cholesterol and mean non-HDL cholesterol, and associated standard errors and sample sizes, by sex and age group (18–19 years, 20–29 years, followed by 10-year age groups and 80+ years). All analyses incorporated appropriate sample weights and complex survey design in calculating age–sex-specific means when applicable. To ensure summaries were prepared according to the study protocol, computer code was provided to NCD-RisC members who requested assistance. All submitted data were checked independently by at least two researchers. Questions and clarifications were discussed with NCD-RisC members and resolved before the data were incorporated in the database.

Finally, we obtained data not accessed through the above routes by extracting data from published reports of all additional national health surveys identified through the above-described strategies, as well as eight sites of the WHO Multinational MONItoring of trends and determinants in CArdiovascular disease (MONICA) project that were not deposited in the MONICA Data Centre. Data were extracted from published reports only when reported by sex and in age groups no wider than 20 years. We also used data from a previous pooling study^7^ when such data did not overlap with those accessed through the above routes.

### Data inclusion and exclusion

Data sources were included in NCD-RisC database if: (1) measured data on total, LDL, HDL cholesterol and/or triglycerides were available; (2) study participants were 10 years of age or older; (3) data were collected using a probabilistic sampling method with a defined sampling frame; (4) data were from population samples at the national, subnational (covering one or more subnational regions, more than three urban communities or more than five rural communities) or community (one or a small number of communities) level; (5) data were collected in or after 1950; and (6) data were from the countries and territories listed in Supplementary Table 2.

We excluded all data sources that included only hypercholesterolaemia or dyslipidaemia diagnosis history or medication status without measurement of cholesterol levels. We also excluded data sources on population subgroups for which the lipid profile may differ systematically from the general population, including: (1) studies that had included or excluded people on the basis of their health status or cardiovascular risk; (2) studies for which the participants were only from ethnic minorities; (3) studies that had recruited only specific educational, occupational or socioeconomic subgroups, with the exception noted below; and (4) studies that had recruited participants through health facilities, with the exception noted below.

We used school-based data in countries and for age–sex groups, for which secondary school enrolment was 70% or higher. We used data for which the sampling frame was health insurance schemes in countries in which at least 80% of the population was insured. Finally, we used data collected through general practice and primary-care systems in high-income and central European countries with universal insurance, because contact with the primary-care systems tends to be as good as or better than response rates for population-based surveys. We used data sources regardless of fasting status, because the differences between fasting and non-fasting measurements are negligible for total, non-HDL and HDL cholesterol^39^, and therefore non-fasting lipid profiles are now widely endorsed for the estimation of cardiovascular risk^36,37^.

### Data used in the analysis

For this paper, we used data from the NCD-RisC database for years 1980 to 2018 and individuals aged 18 years and older. A list of the data sources that we used in this analysis and their characteristics is provided in Supplementary Table 1. The data comprised 1,127 population-based measurement surveys and studies that included measurements of blood lipids on 102.6 million participants aged 18 years and older. We had at least one data source for 161 of the 200 countries that we made estimates for, covering 92.4% of the world’s population in 2018 (Extended Data Fig. 1); and at least two data sources for 104 countries (87.5% of the world population). Of these 1,127 sources, 409 (36.3%) sampled from national populations, 250 (22.2%) covered one or more subnational regions, and the remaining 468 (41.5%) were from one or a small number of communities. Regionally, data availability ranged from around 2 data sources per country in sub-Saharan Africa to approximately 35 sources per country in the high-income Asia–Pacific region. In total, 454 data sources (40.3%) were from years before 2000 and the remaining 673 (59.7%) were collected from 2001 onwards.

### Adjusting for the differences in mean cholesterol between portable device and laboratory measurements

In 112 (10%) of the 1,127 data sources used in our analysis (11.5% and 5.8% of age–sex-specific data points for total and HDL cholesterol, respectively) lipids were measured using a portable device. Some portable devices have narrower analytical ranges than laboratory methods, which results in truncations of blood cholesterol data that are outside their range (Supplementary Table 3). This may in turn affect the population mean. Although cholesterol concentrations that fall outside the analytical range are displayed as ‘high’ (above the measurement range) or ‘low’ (below the measurement range) by these devices, different surveys record and code cholesterol concentrations outside the analytical range in different ways, for example using ‘too low’, ‘too high’ and ‘error’ codes; assigning the minimum or maximum value to individuals whose cholesterol was below or above the analytical range, respectively; setting values outside the analytical range to missing; and so on. We used an approach that treated surveys with such data consistently.

Specifically, we first dropped all participants with cholesterol levels below and at the minimum, and at and above the maximum, values of the analytical range of each portable device before calculating the mean cholesterol (Supplementary Table 3). We then developed conversion regressions to adjust the mean cholesterol levels measured using a portable device (calculated over the restricted range, Supplementary Table 3) to the levels expected using laboratory measurements. The dependent variable in each regression was mean total, non-HDL or HDL cholesterol for the full range, and the main independent variable was mean total, non-HDL or HDL cholesterol over the above-mentioned restricted cholesterol range of the portable devices. The regression coefficients were estimated from data sources for which lipids were measured in a laboratory, and thus had the full range of measurement and could be used to calculate both dependent and independent variables. When estimating the regression coefficients, we constructed the dependent variable using the full data, and the independent variable by dropping the values outside the above-mentioned restricted cholesterol range of each device, mimicking those that would be expected if a portable device had been used. Separate models were developed according to the specific range of the different portable devices. All regressions included terms for age and sex, as well as interactions between predictors and age and sex, based on the Bayesian information criterion^47^. The regressions for mean non-HDL cholesterol also included mean total cholesterol and mean HDL cholesterol because non-HDL cholesterol is calculated from total cholesterol and HDL cholesterol. We excluded data points for which there were fewer than 25 individuals for the purpose of estimating the coefficients of these regressions. All sources of uncertainty in the conversion—including the sampling uncertainty of the original data, the uncertainty of the regression coefficients and residuals—were carried forward by using repeated draws from their respective distributions. The regression coefficients and number of data points used to estimate the coefficients are shown in Supplementary Table 4.

### Statistical analysis

We used a statistical model to estimate mean total, non-HDL and HDL cholesterol by country, year, sex and age using all of the available data. The model is described in detail in a statistical paper and related substantive papers^8,32,33,48^; the computer code is available at http://www.ncdrisc.org/. In summary, we organized countries into 21 regions, mainly based on geography and national income; these regions were further aggregated into 9 ‘super-regions’ (Supplementary Table 2). The model had a hierarchical structure in which estimates for each country and year were informed by its own data, if available, and by data from other years in the same country and from other countries, especially countries in the same region or super-region with data for similar time periods. The extent to which estimates for each country-year are influenced by data from other years and other countries depends on whether the country has data, the sample size of data, whether or not they are national, and the within-country and within-region data variability. The model incorporated nonlinear time trends comprising linear terms and a second-order random walk. The age association of blood lipids was modelled using a cubic spline to allow nonlinear age patterns, which might vary across countries. The model accounted for the possibility that blood lipids in subnational and community samples might systematically differ from nationally representative ones; and/or have larger variation. These features were implemented by including data-driven fixed-effect and random-effect terms for subnational and community data. The fixed effects adjust for systematic differences between subnational or community studies and national studies. The random effects allow national data to have larger influence on the estimates than subnational or community data with similar sample sizes. The model also accounted for rural–urban differences in blood lipids, through the use of data-driven fixed effects for rural-only and urban-only studies. These rural and urban effects were weighted by the difference between study-level and country-level urbanization in the year in which the study was done. The proportion of the national population living in urban areas was also included as a predictor (covariate) in the model. The model for mean non-HDL and HDL cholesterol also used age-standardized mean total cholesterol as a covariate.

We fitted the statistical model with the Markov chain Monte Carlo (MCMC) algorithm, and obtained 5,000 post-burn-in samples from the posterior distribution of model parameters, which were in turn used to obtain the posterior distributions of mean total, non-HDL and HDL cholesterol. We calculated average change in mean total, HDL and non-HDL cholesterol across the 39 years of analysis (reported as change per decade). Age-standardized estimates were generated by taking weighted averages of age–sex-specific estimates, using the WHO standard population. Estimates for regions and the world were calculated as population-weighted averages of the constituent country estimates by age group and sex. The reported credible intervals represent the 2.5–97.5th percentiles of the posterior distributions. We also report the posterior probability that an estimated increase or decrease represents a truly increasing or decreasing trend as opposed to a chance observation. We performed all analyses by sex, because blood lipids levels and trends are different in men and women.

### Validation of statistical model

We tested how well our statistical model predicts missing data, known as external predictive validity, in two different tests. In the first test, we held out all data from 10% of countries with data (that is, created the appearance of countries with no data where we actually had data). The countries for which the data were withheld were randomly selected from the following three groups: data rich (5 or more data sources, with at least one data source after the year 2000), data poor (1 data source) and average data availability (2–4 data sources). In the second test, we assessed other patterns of missing data by holding out 10% of our data sources, again from a mix of data-rich, data-poor and average-data countries, as defined above. For a given country, we either held out a random half of the data of a country or all of the 2000–2018 data of the country to determine, respectively, how well we filled in the gaps for countries with intermittent data and how well we estimated in countries without recent data. In both tests, we then fitted the model to the remaining 90% of the countries (test 1) or data sources (test 2) and made estimates of the held-out observations. We repeated each test five times, holding out a different subset of data in each repetition. In both tests, we calculated the differences between the held-out data and the estimates. We also calculated the 95% credible intervals of the estimates; in a model with good external predictive validity, 95% of held-out values would be included in the 95% credible intervals.

Our statistical model performed well in the external validation tests, that is, in estimating mean cholesterol when data were missing. The estimates of mean total, non-HDL and HDL cholesterol were unbiased, as evidenced with median errors that were very close to zero globally for every outcome and test, and less than ±0.30 mmol l^−1^ in every subset of withheld data except for women in the high-income Asia–Pacific region in test 1 for non-HDL cholesterol (median error 0.47 mmol l^−1^) and men in south Asia in test 2 for non-HDL cholesterol (median error −0.33 mmol l^−1^) (Supplementary Table 5). The 95% credible intervals of estimated means covered 83–92% and 75–83% of true data globally in the first and second tests, respectively. In subsets, coverage ranged from 47% to 100%, but was mostly greater than 75%, with coverage generally lower in test 2 than test 1. Median absolute errors ranged from 0.07 to 0.23 mmol l^−1^ globally for different outcomes and sexes, and were no more than 0.45 mmol l^−1^ in all subsets of withheld data, except for women in the high-income Asia–Pacific region for non-HDL cholesterol in test 1 (median absolute error 0.47 mmol l^−1^).

### Calculation of the number of deaths attributable to high cholesterol

We estimated the number of deaths from IHD and ischaemic stroke attributable to high non-HDL cholesterol. For each country, year, sex and age group, we first calculated the population attributable fractions—that is, the proportion of deaths from IHD and ischaemic stroke that would have been prevented if non-HDL cholesterol levels were at an optimal level (defined as a mean of 1.8–2.2 mmol l^−1^) in the population^6,49^. For these calculations, we used age-specific relative risks from meta-analyses of prospective cohort studies^4,5,50^. The number of IHD and ischaemic stroke deaths attributable to high non-HDL cholesterol was calculated for each country–year–age–sex group by multiplying the cause-specific population attributable fractions by the cause-specific deaths from the Global Burden of Disease study in 1990 and 2017 (the earliest and latest years with cause-specific mortality data).

### Strengths and limitations

The strengths of our study include its scope in making consistent and comparable estimates of trends in blood cholesterol and its cardiovascular disease mortality burden, over almost four decades for all of the countries in the world, including global estimates of non-HDL and HDL cholesterol. We used a large amount of population-based data, which came from countries in which 92% of the global adult population lives. We used only data from studies that had measured blood lipids to avoid bias in self-reported data. Data were analysed according to a consistent protocol, and the characteristics and quality of data from each country were rigorously verified through repeated checks by NCD-RisC members. We pooled data using a statistical model that took into account the epidemiological features of cholesterol, including nonlinear time trends and age associations. Our statistical model used all available data while giving more weight to national data than to subnational and community sources.

Similar to all global analyses, our study is affected by some limitations. Despite our extensive efforts to identify and access worldwide population-based data, some countries had no or few data sources, especially those in sub-Saharan Africa, the Caribbean, central Asia and Melanesia. Estimates for these countries relied mostly or entirely on the statistical model, which shares information across countries and regions through its hierarchy. Data scarcity is reflected in wider uncertainty intervals of our estimates for these countries and regions, highlighting the need for national NCD-oriented surveillance. The distribution of lipids measured in a population using a portable device, which was used in 10% of our studies, may be truncated and may therefore affect the population mean. To overcome this issue, we developed conversion regressions to adjust mean cholesterol levels measured using a portable device to the levels expected in laboratory measurements; the conversion regressions used for this purpose had good predictive accuracy. Although most studies had measured cholesterol in serum samples, around 7% had used plasma samples. As cholesterol measured in plasma and serum samples differ^51^ by only about 3%, adjusting for plasma-serum differences would have little effect on our results, as seen in a previous analysis^14^. Although methods to measure total and HDL cholesterol have evolved over time, since the 1950s there have been systematic efforts to standardize lipid measurements that have resulted in increased comparability between different methods. In our analysis, 90% of studies measured lipids in a laboratory; of these studies more than 60% for total cholesterol and more than 70% for HDL cholesterol participated in a lipid standardization programme or quality control scheme. We did not analyse emerging lipid markers such as apolipoprotein B and apolipoprotein A-I, because they are neither commonly measured in population-based health surveys, nor routinely used in clinical practice^36^.

### Comparison with other studies

There are no global analyses on trends in lipid fractions for comparison with our results. Our findings for total cholesterol were largely consistent with the only other previous analysis^7^, but we estimated a larger decrease in mean total cholesterol in high-income western countries and central Europe, and a larger increase in southeast Asia, because we had an additional decade of data compared with the earlier global analysis. Therefore, although the highest mean total cholesterol levels reported previously^7^, for 2008, were still in high-income western countries, we estimated that in 2018 total cholesterol was equally high or higher in southeast Asia. Our findings on mean total cholesterol trends are also largely consistent with previous multi- and single-country reports^14,15,17–21,52–73^. Differences from previous studies—for example, in Italy^61^, Lithuania^63^, the Netherlands^65^, Russian Federation^69^ and in some countries that participated in the MONICA Project^52^—mostly arise because our study covered a longer period and used a larger number of data sources. Studies^15,18,54,63,66,70,74–77^ that have reported trends in lipid fractions for a period longer than 15 years have found changes in non-HDL cholesterol (or in LDL cholesterol for some studies) that were consistent with our results.

### Reporting summary

Further information on research design is available in the Nature Research Reporting Summary linked to this paper.

## Online content

Any methods, additional references, Nature Research reporting summaries, source data, extended data, supplementary information, acknowledgements, peer review information; details of author contributions and competing interests; and statements of data and code availability are available at 10.1038/s41586-020-2338-1.

## Supplementary information

Supplementary Information This file contains a Literature search for additional data sources and Supplementary Tables 1-5 and Supplementary Figure 1.

Reporting Summary

## Figures and Tables

**Extended Data Fig. 5:**
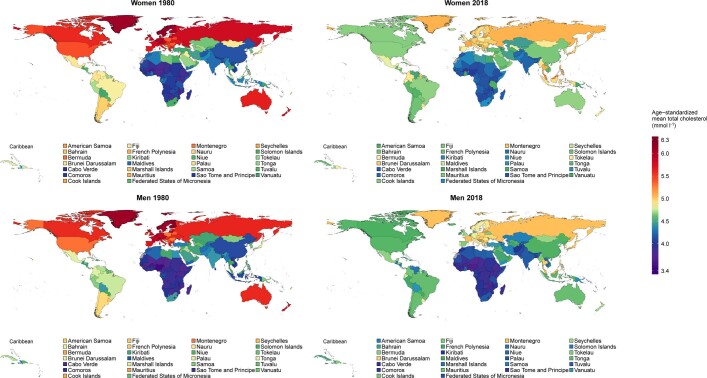
Age-standardized mean total cholesterol by country in 1980 and 2018 for women and men. One mmol l^−1^ is equivalent to 38.61 mg dl^−1^.

**Extended Data Fig. 7:**
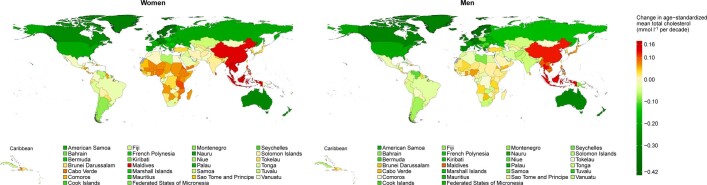
Change per decade in age-standardized mean total cholesterol by country for women and men. One mmol l^−1^ is equivalent to 38.61 mg dl^−1^.

**Extended Data Fig. 8:**
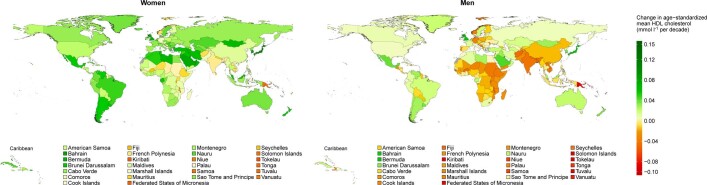
Change per decade in age-standardized mean HDL cholesterol by country for women and men. One mmol l^−1^ is equivalent to 38.61 mg dl^−1^.

## Data Availability

Estimates of mean total, non-HDL and HDL cholesterol by country, year and sex are available at http://www.ncdrisc.org/. Input data from publicly available sources can also be downloaded from http://www.ncdrisc.org/. For other data sources, contact information for data providers can be obtained from http://www.ncdrisc.org/.

## Code availability

The computer code for the Bayesian hierarchical model used in this work is available at http://www.ncdrisc.org/.
