# Supplementary material for: Repositioning of the global epicentre of non-optimal cholesterol
Source: Nature. 2020 Jun 3;582(7810):73–7. doi: 10.1038/s41586-020-2338-1 (PMC7332422; doi:10.1038/s41586-020-2338-1)
Supplement: Supplementary file 1 — This file contains a Literature search for additional data sources and Supplementary Tables 1-5 and Supplementary Figure 1. [file 41586_2020_2338_MOESM1_ESM.pdf]

nature portfolio

https://doi.org/10.1038/s41586-020-2338-1

Supplementary information

Repositioning of the global epicentre of non-optimal cholesterol

In the format provided by the authors and unedited

nature

https://doi.org/10.1038/s41586-020-2338-1

Supplementary information

Repositioning of the global epicentre of non-optimal cholesterol

In the format provided by the authors and unedited

NCD Risk Factor Collaboration (NCD-RisC)

Supplementary Information. Literature search for additional data sources.

To identify any major sources not accessed through the routes described in Methods, we searched Medline (via PubMed) for articles published between 1st January 1950 and 23rd April 2015 using the search terms ("Cholesterol"[Mesh] OR "Lipoproteins, LDL"[Mesh] OR "Lipoproteins, HDL"[Mesh] OR "Hypercholesterolemia"[Mesh] OR "Cholesterol Esters"[Mesh] OR "Dyslipidemias"[Mesh:NoExp] OR "Hyperlipidemias"[Mesh:NoExp]) AND ("Humans"[Mesh]). We excluded the USA from the search because multiple nationally representative health examination surveys with individual records were publicly accessible and accessed before the search had begun. Articles identified through this search were screened according to the inclusion and exclusion criteria described in Methods. The number of articles identified and retained is summarised in Supplementary Figure 1. We contacted the corresponding authors of all eligible studies and invited them to join NCD-RisC. We did similar searches for other cardiometabolic risk factors including body mass index (BMI),[1] height,[2] diabetes,[3] and blood pressure.[4] All eligible studies were invited to analyse data on all cardiometabolic risk factors and join NCD-RisC.

Supplementary Table 1. Data sources used in the analysis.

	Country	Data years	Survey/Study name/Citation	Level of representative-ness	Rural, urban or both	Age range as used for global analysis		Sample size as used for global analysis (Total cholesterol)		Sample size as used for global analysis (HDL cholesterol)		Sample size as used for global analysis (Non-HDL cholesterol)		Device used for measuring total cholesterol*	Device used for measuring HDL cholesterol*	Note
						Male	Female	Male	Female	Male	Female	Male	Female			
1	Afghanistan	2018	STEPS	National	both	18-69	18-69	1,507	1,323					CardioChek		
2	Albania	2001	Shapo et al., J Epidemiol Community Health 2003; 57: 734-9	Community	urban	25+	25+	535	585							
3	Algeria	2003	STEPS	Subnational	both	25-64	25-64	1,575	2,408	148	235	147	234			
4	Algeria	2005	Transition and Health Impact in North Africa	National	both	35-69	35-69	1,250	1,959					Accutrend		
5	Algeria	2007-2009	The ISOR (InSulino-resistance in ORan) study	Community	urban	30-64	30-64	378	409	376	408	376	408			
6	Algeria	2016-2017	STEPS	National	both	18-69	18-69	2,761	3,407							
7	American Samoa	1994	McGarvey, Pac Health Dialog 2001; 8: 157-62	National	both	29+	29-79	141	214	132	210	132	210			
8	American Samoa	2004	STEPS	National	both	25-64	25-64	542	667					Accutrend		
9	Argentina	2004-2005	CArdiovascular Risk factors Multiple Evaluation in Latin America (CARMELA)	Community	urban	25-64	25-64	734	748	734	748	734	748			
10	Argentina	2005	Encuensta Nacional de Nutrición y Salud 2005	National	both		18-49		3,813							
11	Argentina	2011-2012	CESCAS Study	Community	urban	30-79	30-79	1,560	2,351	1,559	2,350	1,559	2,350			
12	Argentina	2018	Encuesta Nacional de Factores de Riesgo 2018	National	both	18+	18+	1,786	2,626					Accutrend		
13	Armenia	2016	STEPS	National	both	18-69	18-69	482	1,163	519	1,237	474	1,156	CardioChek	CardioChek	
14	Australia	1980	Risk Factor Prevalence Study	National	urban	25-64	25-64	2,739	2,756	2,547	2,564	2,546	2,564			
15	Australia	1983	MONICA, Newcastle	Subnational	urban	35-64	35-64	1,197	1,194	1,150	1,169	1,147	1,160			
16	Australia	1983	Risk Factor Prevalence Study	National	urban	25-64	25-64	3,655	3,732	3,650	3,759	3,596	3,711			
17	Australia	1988-1989	Dubbo Study of Australian Elderly	Community	urban	59+	59+	877	1,216	875	1,215	875	1,215			
18	Australia	1988-1989	MONICA, Newcastle	Subnational	urban	35-64	35-64	668	654	666	654	666	654			
19	Australia	1988-1989	MONICA, Newcastle	Community	urban	25-34	25-34	70	83	70	83	70	83			
20	Australia	1989	Risk Factor Prevalence Study	National	urban	20-69	20-69	4,501	4,611	4,501	4,611	4,500	4,611			
21	Australia	1992-1993	Australia Longitudinal Study of Ageing	Community	urban	65+	65+	600	550	599	550	599	550			
22	Australia	1994	MONICA, Newcastle	Subnational	urban	35-64	35-64	627	676	625	675	625	675			
23	Australia	1994	MONICA, Perth inner	Community	urban	25-64	25-64	356	338	356	338	356	338			
24	Australia	1994	MONICA, Perth outer	Community	urban	25-64	25-64	370	374	370	374	370	374			
25	Australia	1999-2000	The Australian Diabetes, Obesity and Lifestyle Study 1999-2000	National	both	25+	25+	5,043	6,136	5,041	6,135	5,041	6,135			
26	Australia	1999-2003	North West Adelaide Health Study	Community	urban	18+	18+	1,902	2,103	1,903	2,103	1,901	2,103			
27	Australia	2004-2005	The Australian Diabetes, Obesity and Lifestyle Study 2004-2005	National	both	30+	30+	2,852	3,444	2,851	3,442	2,851	3,442			
28	Australia	2004-2006	North West Adelaide Health Study	Community	urban	20+	20+	1,513	1,674	1,513	1,674	1,513	1,674			
29	Australia	2008-2010	North West Adelaide Health Study	Community	urban	24+	24+	1,153	1,301	1,153	1,301	1,153	1,301			
30	Australia	2011-2012	Australian Health Survey	National	both	20+	20+	4,147	5,123	4,147	5,123	4,147	5,123			
31	Australia	2012	The Australian Diabetes, Obesity and Lifestyle Study 2012	National	both	37+	37+	2,040	2,530	2,040	2,531	2,040	2,530			
32	Austria	1986	CINDI; Schwarz et al., Eur J Epidemiol 1992; 8: 40-7	Community	urban	25-64	25-64	657	715							
33	Austria	1991	CINDI survey Vorarlberg/Austria	Subnational	both	25-64	25-64	697	736	653	686	653	686			
34	Austria	1992	Vorarlberg Health Monitoring and Promotion Programme	Subnational	both	18+	18+	14,162	18,837							
35	Austria	1998	Vorarlberg Health Monitoring and Promotion Programme	Subnational	both	18+	18+	16,154	20,918							
36	Austria	1998-1999	CINDI survey Vorarlberg/Austria	Subnational	both	25-64	25-64	347	350	346	349	346	349			
37	Austria	2004	Vorarlberg Health Monitoring and Promotion Programme	Subnational	both	18+	18+	20,162	23,893							
38	Austria	2010-2012	Austrian Study on Nutritional Status 2012	National	both	18-80	18-80	246	356	245	356	245	356			
39	Azerbaijan	2017	STEPS	National	both	18-69	18-69	1,012	1,502	1,079	1,561	1,009	1,493	CardioChek	CardioChek	

	Country	Data years	Survey/Study name/Citation	Level of representative-ness	Rural, urban or both	Age range as used for global analysis		Sample size as used for global analysis (Total cholesterol)		Sample size as used for global analysis (HDL cholesterol)		Sample size as used for global analysis (Non-HDL cholesterol)		Device used for measuring total cholesterol*	Device used for measuring HDL cholesterol*	Note
						Male	Female	Male	Female	Male	Female	Male	Female			
40	Barbados	2011-2013	Health of the Nation (HotN)	National	both	25+	25+	412	652	389	606	381	597	Reflotron	Reflotron	
41	Belarus	2016-2017	STEPS	National	both	18-69	18-69	1,963	2,751	1,957	2,700	1,914	2,666	CardioChek	CardioChek	
42	Belgium	1983-1985	MONICA, Luxembourg	Community	urban	35-64	35-64	978	949	973	946	973	946			
43	Belgium	1984-1985	Belgian Interuniversity Research on Nutrition and Health	National	both	25-74	25-74	5,735	5,037	5,668	5,017	5,667	5,015			
44	Belgium	1985-1987	MONICA, Charleroi	Community	urban	25-64	25-64	337	304	334	304	334	304			
45	Belgium	1985-1987	MONICA, Ghent	Community	urban	25-64	25-64	537	434	531	432	531	432			
46	Belgium	1985-1990	Flemish Study on Environment, Genes and Health Outcomes	Community	rural	20-90	20-90	635	653	468	481	468	481			
47	Belgium	1987-1990	MONICA, Charleroi	Community	urban	25-64	25-64	305	266	302	266	302	266			
48	Belgium	1988-1990	MONICA, Ghent	Community	urban	25-64	25-64	443	416	441	415	441	415			
49	Belgium	1990-1992	MONICA, Ghent	Community	urban	25-64	25-64	498	445	498	445	498	445			
50	Belgium	1990-1993	MONICA, Charleroi	Community	urban	25-64	25-64	298	271	296	266	296	266			
51	Belgium	1991-1994	Flemish Study on Environment, Genes and Health Outcomes	Community	rural	26-88	26-88	388	396	386	396	386	396			
52	Belgium	1996-1998	Flemish Study on Environment, Genes and Health Outcomes	Community	rural	18-84	18-84	345	335	343	334	343	334			
53	Belgium	1998	Flemish Study on Environment, Genes and Health Outcomes	Community	rural	32-86	32-86	319	351	319	351	319	351			
54	Belgium	1999-2001	Flemish Study on Environment, Genes and Health Outcomes	Community	rural	18-81	18-79	212	226	212	226	212	226			
55	Belgium	2001	Flemish Study on Environment, Genes and Health Outcomes	Community	rural	18-78	18-78	228	208	228	208	228	208			
56	Belgium	2002-2003	Flemish Study on Environment, Genes and Health Outcomes	Community	rural	18-81	18-81	167	180	167	180	167	180			
57	Belgium	2003	The European Male Ageing Study	Community	both	40+		444		443		443				
58	Belgium	2002-2005	Flemish Study on Environment, Genes and Health Outcomes	Community	rural	18-88	18-88	386	397	385	397	385	397			
59	Belgium	2005-2008	Flemish Study on Environment, Genes and Health Outcomes	Community	rural	18-89	18-89	445	450	445	450	445	450			
60	Belgium	2008	The European Male Ageing Study	Community	both	40+		377		378		376				
61	Belgium	2009-2013	Flemish Study on Environment, Genes and Health Outcomes	Community	rural	20-88	20-88	328	332	327	331	327	331			
62	Belgium	2010-2015	Flemish Study on Environment, Genes and Health Outcomes	Community	rural	18-87	18-87	386	396	386	396	386	396			
63	Belize	2004-2005	CAMDI	National	both	20+	20+	600	1,021	600	1,019	600	1,019			
64	Benin	2008	STEPS	National	both	25-64	25-64	758	937					Accutrend		
65	Benin	2015	STEPS	National	both	18-69	18-69	1,843	2,189					CardioChek		
66	Bhutan	2007	STEPS	Community	urban	25-74	25-74	1,126	1,320	1,049	1,223	1,048	1,221			
67	Bhutan	2014	STEPS	National	both	18-69	18-69	877	1,425					CardioChek		
68	Bolivia	2005-2007	Cardiovascular and metabolic syndrome risk assessment of Bolivian school children and adolescents – Relationships to obesity, diabetes, income, food intake and physical activity	National	both	18-18	18-18	108	109	101	103	98	102			
69	Bosnia and Herzegovina	2012	Non-communicable disease risk factor survey, Federation of B&H	Subnational	rural	18+	18+	1,159	1,253							

	Country	Data years	Survey/Study name/Citation	Level of representative-ness	Rural, urban or both	Age range as used for global analysis		Sample size as used for global analysis (Total cholesterol)		Sample size as used for global analysis (HDL cholesterol)		Sample size as used for global analysis (Non-HDL cholesterol)		Device used for measuring total cholesterol*	Device used for measuring HDL cholesterol*	Note
						Male	Female	Male	Female	Male	Female	Male	Female			
70	Bosnia and Herzegovina	2012	Non-communicable disease risk factor survey, Federation of B&H	Subnational	urban	18+	18+	571	688							
71	Botswana	2014	STEPS	National	both	18-69	18-69	764	1,740	1,045	2,099	729	1,625	CardioChek	CardioChek	
72	Brazil	1991	Fornes et al., Rev Saude Publica 2000; 34: 380-7	Community	urban	25+	25+	387	548							
73	Brazil	1992	EPIDOSO; Ramos, Cad Saude Publica 2003; 19: 793-8	Community	urban	65+	65+	158	280							
74	Brazil	1996-1997	The Bambui Cohort Study of Ageing	Community	urban	18+	18+	947	1,368	947	1,368	947	1,368			
75	Brazil	1998	Belo Horizonte Heart Study	Community	urban	18-18	18-18	31	46	31	46	31	46			
76	Brazil	2000	The 1982 Pelotas (Brazil) Birth Cohort: 18 years follow-up	Community	urban	18-18		2,079		2,059		2,055				
77	Brazil	1999-2000	Prevalence of Risk Factors for Coronary Artery Disease in the State of Rio Grande do Sul	Subnational	urban	20+	20+	463	519							
78	Brazil	2004	Caju & Virgen das Gracas	Community	rural	18+	18+	268	275	268	275	268	275			
79	Brazil	2002-2004	Ribeira Preto Birth Cohort	Community	urban	22-25	22-25	1,007	1,067	1,006	1,067	1,006	1,067			
80	Brazil	2003-2005	São Paulo Health & Ageing Study	Community	urban	65+	65+	762	1,190	762	1,190	762	1,190			
81	Brazil	2004-2006	Hearts of Brazil	National	urban	18-79	18+	307	336					Accutrend		
82	Brazil	2004-2005	The 1982 Pelotas (Brazil) Birth Cohort: 23 years follow-up	Community	urban	23-23	23-23			1,918	1,906					
83	Brazil	2008	The Bambui Cohort Study of Ageing	Community	urban	71+	71+	253	473	253	473	253	473			
84	Brazil	2008	Caju & Virgen das Gracas	Community	rural	18+	18+	249	278	248	278	248	278			
85	Brazil	2010	San Pedro	Community	rural	18+	18+	150	208	150	208	150	208			
86	Brazil	2011-2012	The 1993 Pelotas (Brazil) Birth Cohort: 18 years follow-up	Community	urban	18-19	18-19	1,916	1,925	1,916	1,925	1,916	1,925			
87	Brazil	2010-2015	Baependi Heart Study	Community	rural	18+	18+	1,064	1,425	1,063	1,424	1,061	1,424			
88	Brazil	2012-2013	The 1982 Pelotas (Brazil) Birth Cohort: 30 years follow-up	Community	urban	30-30	30-30	1,741	1,791	1,741	1,791	1,741	1,791			
89	Brazil	2013	Pesquisas Nacional de Saude	National	both	18+	18+	2,761	3,103	2,750	3,096	2,749	3,096			
90	Brazil	2011-2014	Profile of Risk Factors for Coronary Arterial Disease in Rio Grande Do Sul - Revaluation after 10 years	Subnational	urban	20+	20+	365	464	336	411	336	410			
91	Brazil	2014-2015	EpiFloripa Cohort Study of Ageing - Wave 2, Clinical and Laboratory Exams	Community	urban	63+	63+	209	387	209	387	209	387			
92	Brazil	2014-2015	EpiFloripa Adults Cohort Study (EpiFloripa)	Community	urban	25-65	25-65	299	411	296	411	296	411			
93	Brazil	2015-2016	The 1993 Pelotas (Brazil) Birth Cohort: 22 years follow-up	Community	urban	21-23	21-23	1,660	1,818	1,659	1,818	1,659	1,818			
94	Brunei Darussalam	2010-2011	National Health And Nutritional Status Survey (NHANSS)	National	both	20-75	20-75	629	762	628	762	628	761			
95	Brunei Darussalam	2015-2016	National Non-Communicable Diseases Survey (NNCDS)	National	both	18-69	18-69	835	1,103	830	1,096	828	1,093			
96	Burkina Faso	2013	STEPS	National	both	25-64	25-64	952	1,144	1,827	1,912	876	1,068	CardioChek	CardioChek	
97	Cabo Verde	2007	STEPS	National	both	25-64	25-64	118	282					Accutrend		
98	Cambodia	2010	STEPS	National	both	25-64	25-64	1,264	2,502					Accutrend		
99	Canada	1985-1988	MONICA, Halifax	Community	both	25-64	25-64	423	414	421	414	421	414			
100	Canada	1986-1992	Canada Heart Health Survey	National	both	18-74	18-74	9,592	9,804	9,515	9,747	9,511	9,746			
101	Canada	1995	MONICA, Halifax	Community	both	25-64	25-64	269	281	268	281	268	281			
102	Canada	1995-1997	Canadian Multicentre Osteoporosis Study (CaMos)	Community	both	50+	50+	139	369	139	369	139	369			
103	Canada	2005-2008	Canadian Multicentre Osteoporosis Study (CaMos)	Subnational	both	50+	50+	605	1,466	605	1,466	605	1,466			
104	Canada	2007-2009	Canadian Health Measures Survey, Cycle 1	National	both	20-79	20-79	1,607	1,805	1,607	1,805	1,607	1,805			

	Country	Data years	Survey/Study name/Citation	Level of representative-ness	Rural, urban or both	Age range as used for global analysis		Sample size as used for global analysis (Total cholesterol)		Sample size as used for global analysis (HDL cholesterol)		Sample size as used for global analysis (Non-HDL cholesterol)		Device used for measuring total cholesterol*	Device used for measuring HDL cholesterol*	Note
						Male	Female	Male	Female	Male	Female	Male	Female			
105	Canada	2009-2011	Canadian Health Measures Survey, Cycle 2	National	both	20-79	20-79	1,672	1,907	1,674	1,908	1,672	1,907			
106	Canada	2012-2013	Canadian Health Measures Survey, Cycle 3	National	both	20-79	20-79	1,560	1,574	1,560	1,574	1,560	1,574			
107	Canada	2014-2015	Canadian Health Measures Survey, Cycle 4	National	both	20-79	20-79	1,552	1,551	1,550	1,551	1,550	1,551			
108	Canada	2016-2017	Canadian Health Measures Survey, Cycle 5	National	both	20-79	20-79	1,512	1,502	1,511	1,502	1,510	1,502			
109	Chile	1992-1993	Miquel et al., Gastroenterology 1998; 115: 937-46	Community	urban	18+	18+	660	1,032	659	1,032	659	1,032			
110	Chile	2000	Nervi et al., J Hepatol 2006; 45: 299-305	Community	urban	20+	20+	335	625	334	625	334	625			
111	Chile	2003	Encuesta Nacional de Salud	National	both	18+	18+	904	1,055	901	1,055	901	1,055			
112	Chile	2004-2005	CArdiovascular Risk factors Multiple Evaluation in Latin America (CARMELA)	Community	urban	25-64	25-64	783	872	783	872	783	872			
113	Chile	2009-2010	Encuesta Nacional de Salud	National	both	18+	18+	1,066	1,550	1,065	1,549	1,065	1,549			
114	Chile	2011-2012	CESCAS Study	Community	urban	30-79	30-79	895	976	895	976	895	976			
115	Chile	2016-2017	Encuesta Nacional de Salud	National	both	18+	18+	1,299	2,274	1,299	2,274	1,299	2,274			
116	China	1983	Sino-MONICA Shanghai	Community	rural	30-64	30-64	621	627	618	624	618	624			
117	China	1984-1985	Sino-MONICA Beijing	Community	both	25-64	25-64	816	857	814	854	814	854			
118	China	1988	Sino-MONICA Hebei	Community	both	25-64		704		686		686				
119	China	1988	Sino-MONICA Heilongjiang	Community	urban	25-64	25-64	800	800	800	800	800	800			
120	China	1988	Sino-MONICA Henan	Community	urban	25-64	25-64	345	427	345	427	345	427			
121	China	1988	Sino-MONICA Neimenggu	Community	urban	25-64	25-64	397	400	349	400	349	400			
122	China	1988	Sino-MONICA Sichuan	Community	both	25-64	25-64	313	333	312	333	312	333			
123	China	1988	Sino-MONICA Shandong	Community	urban	25-64	25-64	211	221	211	221	211	221			
124	China	1986-1989	Sino-MONICA Shanghai	Community	rural	25-64	25-64	674	752	675	753	674	751			
125	China	1988-1989	Sino-MONICA Beijing	Community	both	25-64	25-64	700	861	697	860	696	859			
126	China	1989	Sino-MONICA Fujian	Community	urban	25-64	25-64	178	189	178	189	178	189			
127	China	1988-1989	Sino-MONICA Jilin	Community	urban	25-64	25-64	391	408	378	388	378	388			
128	China	1989	Sino-MONICA Jiangsu	Community	rural	25-64	25-64	397	399	398	399	397	399			
129	China	1988-1989	Sino-MONICA Jiangxi	Community	urban	25-64	25-64	348	360	349	360	348	360			
130	China	1988-1989	Sino-MONICA Liaoning	Community	both	25-64	25-64	728	733	728	733	728	732			
131	China	1991	Sino-MONICA Shanghai	Community	rural	30-64	30-64	564	624	564	624	564	624			
132	China	1992-1993	Anzhen 02 Cohort Study	Community	urban	34-65	34-65	2,029	2,112	2,023	2,109	2,023	2,107			
133	China	1991-1992	Fangshan Cohort Study	Community	urban	34-79	34-69	272	562	258	544	256	537			
134	China	1992	Huashan Study	Community	urban	35-75	35-75	866	936	863	933	863	932			
135	China	1992	Sino-MONICA Sichuan	Community	both	25-64	25-64	626	537	626	537	626	537			
136	China	1993	Sino-MONICA Anhui	Community	urban	25-64	25-64	192	195	192	195	192	195			
137	China	1993	Sino-MONICA Beijing	Community	both	25-64	25-64	609	814	609	814	609	814			
138	China	1993	Sino-MONICA Jiangsu	Community	urban	25-64	25-64	462	365	462	365	462	365			
139	China	1993	Sino-MONICA Liaoning	Community	both	25-64	25-64	493	500	492	500	492	500			
140	China	2000-2001	The International Collaborative Study of Cardiovascular Disease in ASIA	National	both	35-74	35-74	7,355	7,860	7,353	7,854	7,350	7,851			
141	China	2002	Fan et al., World J Gastroenterol 2008; 14: 2418-24	Subnational	both	25-84	25-74	5,526	7,766							
142	China	2002	China National Nutrition and Health Survey	National	both	20+	20+	22,747	25,643	22,809	25,713	22,741	25,637			
143	China	2004-2005	Xinjiang Children and Adolescent Survey	Community	urban	18-18	18-18	6	4	6	4	6	4			
144	China	2006	Beijing Eye Study	Community	both	45+	45+	1,247	1,647	1,260	1,664	1,244	1,644			
145	China	2006-2007	Handan Eye Study	Community	rural	30+	30+	2,494	3,120	2,494	3,122	2,492	3,120			
146	China	2008	China Health and Retirement Longitudinal Study (CHARLS), pilot survey	Subnational	both	45+	45+	1,664	1,718	1,674	1,714	1,592	1,646	CardioChek	CardioChek	
147	China	2007-2008	China National Diabetes and Metabolic Disorders Study; Yang et al., Circulation 2012; 125: 2212-21	National	both	20+	20+	18,227	27,530	18,227	27,530	18,227	27,530			

	Country	Data years	Survey/Study name/Citation	Level of representative-ness	Rural, urban or both	Age range as used for global analysis		Sample size as used for global analysis (Total cholesterol)		Sample size as used for global analysis (HDL cholesterol)		Sample size as used for global analysis (Non-HDL cholesterol)		Device used for measuring total cholesterol*	Device used for measuring HDL cholesterol*	Note
						Male	Female	Male	Female	Male	Female	Male	Female			
148	China	2009	China Health and Nutrition Study	National	both	18+	18+	4,035	4,524	4,027	4,514	4,026	4,514			3
149	China	2010	China Noncommunicable Disease Surveillance	National	both	18+	18+	44,378	52,553	44,489	52,822	44,282	52,502			
150	China	2011	Beijing Eye Study	Community	both	50+	50+	1,033	1,419	1,033	1,420	1,033	1,419			
151	China	2011-2012	China Health and Retirement Longitudinal Study (CHARLS), baseline survey	National	both	45+	45+	9,086	10,450	9,078	10,452	9,064	10,436			
152	China	2012-2013	The Kailuan Study	Community	urban	18+	18+	81,363	22,443	81,090	22,400	81,066	22,395			
153	China	2014-2015	The Kailuan Study	Community	urban	18+	20+	73,445	19,261	73,361	19,256	73,304	19,244			
154	China (Hong Kong SAR)	1991	The Hong Kong study on Health, health risk and quality of life in the Chinese elderly cohort	Community	both	70+	70+	94	101	94	101	94	101			
155	China (Hong Kong SAR)	1995-1996	Hong Kong Cardiovascular Risk Factor Prevalence Study 1995-1996	National	both	25-74	25-74	1,409	1,479	1,409	1,479	1,408	1,479			
156	Colombia	2001	CINDI/CARMEN-Bucaramaga; Bautista et al., Eur J Cardiovasc Prev Rehabil 2006; 13:769-75	Community	urban	25-64	25-64	620	1,216							
157	Colombia	2004-2005	CArdiovascular Risk factors Multiple Evaluation in Latin America (CARMELA)	Community	urban	25-64	25-64	738	815	738	815	738	815			
158	Colombia	2007	Encuesta Nacional de Salud	National	both	18-69	18-69	4,943	7,182	4,832	7,340	4,553	6,987	Cholestech	Cholestech	
159	Colombia	2010	STEPS	Subnational	urban	18-64	18-64	429	609					Accutrend		
160	Colombia	2015	STEPS	Subnational	both	18-64	18-64	341	579	341	579	341	578			
161	Comoros	2011	STEPS	National	both	25-64	25-64	267	966					Accutrend		
162	Cook Islands	1981	Egusa et al., Atherosclerosis 1984; 53: 241-54	Community	both	20-59	20-59	138	159							
163	Cook Islands	2013-2015	STEPS	National	both	18-64	18-64	334	400					Accutrend		
164	Costa Rica	1987	Campos et al., Circulation 1992; 85: 648-58	Community	both	25-64	25-64	198	215							
165	Costa Rica	2004	CAMDI	Community	urban	20+	20+	390	756	390	756	390	756			
166	Costa Rica	2004-2006	Costa Rican Longevity and Healthy Aging Study Pre-1945 Cohort Wave 1	National	both	60+	60+	1,208	1,448	1,206	1,443	1,205	1,443			
167	Costa Rica	2006-2008	Costa Rican Longevity and Healthy Aging Study Pre-1945 Cohort Wave 2	National	both	62+	62+	1,018	1,215	1,016	1,215	1,016	1,215			
168	Costa Rica	2010	Costa Rican National Cardiovascular Risk Factors Survey, 2010	National	both	20+	20+	725	1,937	703	1,837	689	1,814			
169	Costa Rica	2010-2011	Costa Rican Longevity and Healthy Aging Study 1945-1955 Cohort Wave 1	National	both	54-66	54-66	1,029	1,590	1,025	1,590	1,025	1,590			
170	Croatia	2008	Endemic Nephropathy and Arterial hypertension (ENAH)	Subnational	rural	18+	18+	343	551	343	551	343	550			
171	Croatia	2010	Endemic Nephropathy and Arterial hypertension (ENAH)	Subnational	rural	18+	18+	259	391	259	391	259	391			
172	Croatia	2015	Endemic Nephropathy and Arterial hypertension (ENAH) Follow-up Study	Subnational	rural	20+	20+	223	461	222	461	222	461			
173	Cuba	2010	National Risk Factor Survey	National	both	15+	15+	1,176	1,441	171	211	171	211			1
174	Cuba	2011	Non communicable disease risk factor in Cienfuegos	Community	urban	15-74	15-74	616	880	616	880	616	880			1
175	Czech Republic	1985	MONICA, Czech Republic	National	both	25-64	25-64	1,244	1,307	1,241	1,306	1,241	1,306			
176	Czech Republic	1988	MONICA, Czech Republic	National	both	25-64	25-64	1,353	1,409	1,353	1,408	1,352	1,408			
177	Czech Republic	1992	MONICA, Czech Republic	National	both	25-64	25-64	1,127	1,189	1,120	1,187	1,120	1,187			
178	Czech Republic	1997-1998	Czech post-MONICA	National	both	25-64	25-64	1,527	1,664	1,519	1,664	1,519	1,664			
179	Czech Republic	2000-2001	Czech post-MONICA	National	both	25-64	25-64	1,612	1,661	1,606	1,656	1,606	1,656			
180	Czech Republic	2005	HELEN	National	urban	45-54	45-54	761	1,063							
181	Czech Republic	2006-2009	Czech post-MONICA	National	both	25-64	25-64	1,718	1,860	1,700	1,820	1,700	1,820			

	Country	Data years	Survey/Study name/Citation	Level of representative-ness	Rural, urban or both	Age range as used for global analysis		Sample size as used for global analysis (Total cholesterol)		Sample size as used for global analysis (HDL cholesterol)		Sample size as used for global analysis (Non-HDL cholesterol)		Device used for measuring total cholesterol*	Device used for measuring HDL cholesterol*	Note
						Male	Female	Male	Female	Male	Female	Male	Female			
182	Czech Republic	2015-2018	MONICA	National	both	25-65	25-65	1,239	1,361	1,238	1,359	1,238	1,359			
183	Denmark	1981-1983	Copenhagen City Heart Study	Subnational	urban	20+	20+	5,612	6,884	5,596	6,870	5,596	6,870			
184	Denmark	1982-1984	MONICA, Glostrup	Community	urban	30-61	30-61	1,938	1,842	1,935	1,842	1,935	1,842			
185	Denmark	1986-1987	MONICA, Glostrup	Community	urban	29-61	29-61	747	755	746	755	746	755			
186	Denmark	1991-1992	MONICA, Glostrup	Community	urban	29-61	29-61	805	809	807	810	805	807			
187	Denmark	1991-1994	Copenhagen City Heart Study	Subnational	urban	20+	20+	4,339	5,581	4,328	5,570	4,328	5,570			
188	Denmark	2001-2003	Copenhagen City Heart Study	Subnational	urban	20+	20+	2,529	3,388	2,525	3,384	2,524	3,384			
189	Denmark	2003-2004	Copenhagen General Population Study 1	Subnational	urban	20+	20+	4,829	5,398	4,827	5,398	4,827	5,398			
190	Denmark	2005	Copenhagen General Population Study 1	Subnational	urban	20+	20+	5,173	6,029	5,169	6,025	5,169	6,025			
191	Denmark	2006	Copenhagen General Population Study 1	Subnational	urban	20+	20+	5,053	4,855	5,052	4,855	5,052	4,855			
192	Denmark	2007	Copenhagen General Population Study 1	Subnational	urban	20+	20+	4,030	6,913	4,027	6,913	4,027	6,913			
193	Denmark	2006-2008	The Health2006 Cohort	Subnational	urban	18-71	20-69	1,548	1,850	1,548	1,849	1,548	1,849			
194	Denmark	2008	Copenhagen General Population Study 1	Subnational	urban	20+	20+	4,742	6,473	4,743	6,470	4,742	6,470			
195	Denmark	2007-2008	The Danish Health Examination Survey 2007-2008	National	both	18+	18+	7,204	10,427							
196	Denmark	2009	Copenhagen General Population Study 1	Subnational	urban	20+	20+	4,217	5,239	4,217	5,239	4,217	5,239			
197	Denmark	2010	Copenhagen General Population Study 1	Subnational	urban	20+	20+	3,923	4,842	3,917	4,838	3,916	4,838			
198	Denmark	2009-2010	The European Youth Heart Study	Community	both	20-28	20-28	303	325	303	325	303	325			
199	Denmark	2011	Copenhagen General Population Study 1	Subnational	urban	20+	20+	4,870	5,738	4,869	5,733	4,868	5,733			
200	Denmark	2012	Copenhagen General Population Study 1	Subnational	urban	20+	20+	4,407	5,276	4,403	5,272	4,403	5,272			
201	Denmark	2011-2012	The Health2006 cohort - 5-year follow-up	Subnational	urban	24-76	24-76	1,056	1,242	1,056	1,242	1,056	1,242			
202	Denmark	2013	Copenhagen General Population Study 1	Subnational	urban	20+	20+	3,940	4,610	3,937	4,610	3,937	4,609			
203	Denmark	2014	Copenhagen General Population Study 2	Subnational	urban	20+	20+	1,354	1,745	1,354	1,743	1,353	1,743			
204	Denmark	2012-2015	Danish study of Functional Disorders (DanFunD)	Subnational	urban	18-72	18-72	3,450	4,015	3,452	4,017	3,450	4,015			
205	Denmark	2014-2015	Copenhagen General Population Study 1	Subnational	urban	20+	20+	2,226	2,815	2,225	2,815	2,224	2,815			
206	Denmark	2015	Copenhagen General Population Study 2	Subnational	urban	20+	20+	3,988	4,980	3,994	4,996	3,982	4,976			
207	Denmark	2016	Copenhagen General Population Study 2	Subnational	urban	20+	20+	4,434	5,417	4,430	5,412	4,427	5,409			
208	Denmark	2017	Copenhagen General Population Study 2	Subnational	urban	20+	20+	3,184	4,399	3,188	4,392	3,184	4,392			
209	Dominica	2007	STEPS	National	both	18-64	18-64	37	69	38	75	35	67	CardioChek	CardioChek	
210	Dominican Republic	1993	Aono et al., J Epidemiol 1997; 7: 238-43	National	both	20-69	20-69	737	1,149							
211	Dominican Republic	1996-1998	Estudio factores de riesgo cardiovascular y sindrome metabolico en la Republica Dominicana I (EFRICARD I)	National	both	18-75	18-75	1,854	3,713	2,085	4,089	1,852	3,706	Reflotron	Reflotron	
212	Dominican Republic	2010-2012	Estudio factores de riesgo cardiovascular y sindrome metabolico en la Republica Dominicana II (EFRICARD II)	National	both	18-75	18-75	1,631	3,284	1,631	3,283	1,629	3,282			
213	Ecuador	2004-2005	CArdiovascular Risk factors Multiple Evaluation in Latin America (CARMELA)	Community	urban	25-64	25-64	813	825	813	825	813	825			
214	Ecuador	2009	National Survey of Health, Wellbeing, and Aging	National	both	60+	60+	1,070	1,293	1,069	1,292	1,069	1,292			
215	Egypt	2003-2004	Metabolic and cardiovascular risk profiles and hepatitis C virus infection in rural Egypt	Community	rural	25+	25+	317	450	317	450	317	450			
216	Egypt	2005	STEPS	National	both	18-65	18-65	934	980	788	912	750	878	Reflotron	Reflotron	
217	Egypt	2007-2009	Hepatitis C infection and clearance: impact on atherosclerosis and cardiometabolic risk factors	Community	rural	35+	35+	640	845	590	804	588	802			
218	Egypt	2011	STEPS	National	both	18-65	18-65	767	1,441	767	1,446	766	1,441			
219	Egypt	2017	STEPS	National	both	18-69	18-69	1,023	2,339	1,396	2,748	967	2,225	CardioChek	CardioChek	
220	El Salvador	2004	CAMDI	Community	urban	20+	20+	407	824							

	Country	Data years	Survey/Study name/Citation	Level of representative-ness	Rural, urban or both	Age range as used for global analysis		Sample size as used for global analysis (Total cholesterol)		Sample size as used for global analysis (HDL cholesterol)		Sample size as used for global analysis (Non-HDL cholesterol)		Device used for measuring total cholesterol*	Device used for measuring HDL cholesterol*	Note
						Male	Female	Male	Female	Male	Female	Male	Female			
221	El Salvador	2014-2015	Encuesta Nacional de Enfermedades Crónicas (ENECA-ELS 2014-2015)	National	both	20+	20+	1,706	2,968	1,695	2,958	1,695	2,958			
222	Eritrea	2010	STEPS	National	both	25-74	25-74	1,216	3,320					Accutrend		
223	Estonia	1985	Volozh et al., Eur J Public Health 2002; 12: 16-21	Community	urban	30-54		1,747								
224	Estonia	1991	Olferev et al., Cor Vasa 1991; 33: 472-9; Site 1	Community	urban		35-54		250							
225	Estonia	1991	Olferev et al., Cor Vasa 1991; 33: 472-9; Site 2	Community	urban		35-54		232							
226	Estonia	1993	Volozh et al., Eur J Public Health 2002; 12: 16-21	Community	urban	30-54		292								
227	Estonia	1997	Johansson et al., J Intern Med 2002; 252: 551-60	Community	urban	35-55	35-55	144	133							
228	Estonia	2003	The European Male Ageing Study	Community	both	40+		426		428		426				
229	Estonia	2008	The European Male Ageing Study	Community	both	40+		312		314		312				
230	Fiji	2002	STEPS	National	both	18-64	20-64	923	1,466	914	1,451	904	1,446	Reflotron	Reflotron	
231	Finland	1980	Young Finns Study 1980 rural	National	rural	18-18	18-18	120	148	120	148	120	148			
232	Finland	1980	Young Finns Study 1980 urban	National	urban	18-18	18-18	131	130	131	130	131	130			
233	Finland	1982	MONICA, North Karelia/Kuopio/Turku/Loimaa	Subnational	both	25-64	25-64	4,555	4,655	4,551	4,654	4,551	4,654			
234	Finland	1983	Young Finns Study 1983 rural	National	rural	18-21	18-21	165	197	163	197	163	197			
235	Finland	1983	Young Finns Study 1983 urban	National	urban	18-21	18-21	184	202	183	202	183	202			
236	Finland	1984	Finnish cohort of the FINE study	Community	rural	65-84		692		693		692				
237	Finland	1986	Young Finns Study 1986 rural	National	rural	18-24	18-24	205	235	205	237	205	235			
238	Finland	1986	Young Finns Study 1986 urban	National	urban	18-24	18-24	259	330	261	331	258	329			
239	Finland	1984-1989	Kuopio Ischaemic Heart Disease Risk factor Study	Subnational	both	42-61		2,615		2,615		2,615				
240	Finland	1987	MONICA, North Karelia/Kuopio/Turku/Loimaa	Subnational	both	25-64	25-64	2,897	3,143	2,895	3,143	2,895	3,143			
241	Finland	1989	Finnish cohort of the FINE study	Community	rural	70-89		445		445		445				
242	Finland	1990-1992	Oulu 35 Study	Community	urban	56-56	56-56	231	327	231	327	231	327			
243	Finland	1992	The National FINRISK Study	Subnational	both	25-64	25-64	2,844	3,106	2,843	3,106	2,843	3,106			
244	Finland	1991-1993	Kuopio Ischaemic Heart Disease Risk factor Study	Subnational	both	46-65		1,032		1,032		1,032				
245	Finland	1997	The National FINRISK Study	National	both	25-74	25-74	4,225	4,087	4,222	4,087	4,222	4,087			
246	Finland	1997	North Finland Birth Cohort 1966	Community	both	30-31	30-31	2,791	268	2,790	268	2,790	268			
247	Finland	1996-1998	Oulu 35 Study	Community	urban	60-63	60-63	244	344	244	344	244	344			
248	Finland	1996-1998	Savitaipale Study, Baseline	Community	rural	40-66	40-66	572	575	569	573	569	573			
249	Finland	2000	Viiri et al., Atherosclerosis 2005; 179: 161-7; Site 1	Community	urban	50-59		74								
250	Finland	2000	Viiri et al., Atherosclerosis 2005; 179: 161-7; Site 2	Community	urban	50-59		101								
251	Finland	2000	Viiri et al., Atherosclerosis 2005; 179: 161-7; Site 3	Community	urban	50-59		42								
252	Finland	2000	Finnish cohort of the FINE study	Community	rural	81-96		96		96		96				
253	Finland	1998-2001	Kuopio Ischaemic Heart Disease Risk factor Study	Subnational	both	53-73	53-73	853	916	853	916	853	916			
254	Finland	2000-2001	Health 2000 Survey	National	both	30+	30+	2,988	3,702	2,988	3,702	2,988	3,702			
255	Finland	2001	Young Finns Study 2001 rural	National	rural	24-39	24-39	344	395	343	395	343	395			
256	Finland	2001	Young Finns Study 2001 urban	National	urban	24-39	24-39	660	770	658	770	658	770			
257	Finland	2002	The National FINRISK Study	National	both	25-74	25-74	3,263	3,762	3,262	3,762	3,262	3,762			
258	Finland	2001-2003	Oulu 45 Study	Community	urban	55-58	55-58	427	550	427	549	427	549			
259	Finland	2001-2004	Helsinki Birth Cohort Study	Community	urban	56-69	56-69	927	1,074	927	1,074	927	1,074			
260	Finland	2005	Mantyselka et al., Rheumatology 2008; 47: 1235-8	Community	rural	30-65	30-65	230	250							
261	Finland	2007	The National FINRISK Study	National	both	25-74	25-74	2,923	3,251	2,923	3,251	2,923	3,251			
262	Finland	2005-2008	Kuopio Ischaemic Heart Disease Risk factor Study	Subnational	both	60-81	60-81	1,240	633	1,240	633	1,240	633			
263	Finland	2007	Oulu 35 Study	Community	urban	72-72	72-72	182	270	182	272	182	270			
264	Finland	2007	Young Finns Study 2007 rural	National	rural	30-45	30-45	378	439	376	438	376	438			
265	Finland	2007	Young Finns Study 2007 urban	National	urban	30-45	30-45	605	719	597	719	597	719			
266	Finland	2008	Control group for Finnish male former elite athletes	National	both	61+		207		207		207				

	Country	Data years	Survey/Study name/Citation	Level of representative-ness	Rural, urban or both	Age range as used for global analysis		Sample size as used for global analysis (Total cholesterol)		Sample size as used for global analysis (HDL cholesterol)		Sample size as used for global analysis (Non-HDL cholesterol)		Device used for measuring total cholesterol*	Device used for measuring HDL cholesterol*	Note
						Male	Female	Male	Female	Male	Female	Male	Female			
267	Finland	2007-2008	Savitaipale Study, Follow-up	Community	rural	51-75	51-75	427	477	427	477	427	477			
268	Finland	2011	Young Finns Study 2011 rural	National	rural	34-49	34-49	365	423	364	423	364	423			
269	Finland	2011	Young Finns Study 2011 urban	National	urban	34-49	34-49	502	632	501	632	501	632			
270	Finland	2012	The National FINRISK Study	National	both	25-74	25-74	2,771	2,998	2,771	2,997	2,771	2,997			
271	Finland	2011-2012	Health 2011 Survey	National	both	30+	30+	1,957	2,416	1,956	2,416	1,956	2,416			
272	Finland	2012	North Finland Birth Cohort 1966	Community	both	45-47	45-47	2,540	3,225	2,542	3,225	2,540	3,225			
273	Finland	2017	The FinHealth Survey	National	both	18+	18+	3,185	3,728	3,185	3,728	3,185	3,728			
274	France	1985-1987	MONICA, Strasbourg	Subnational	both	35-64	35-64	639	667	637	665	637	665			
275	France	1985-1987	MONICA, Strasbourg	Subnational	both	25-34	25-34	64	75	64	75	64	75			
276	France	1985-1987	MONICA, Toulouse	Subnational	both	35-64	35-64	659	606	652	602	652	602			
277	France	1986-1989	MONICA, Lille	Community	urban	25-64	25-64	778	595	773	593	773	593			
278	France	1988-1991	MONICA, Toulouse	Subnational	both	35-64		584		584		584				
279	France	1994-1996	MONICA, Toulouse	Subnational	both	35-64	35-64	609	565	609	565	609	565			
280	France	1995-1997	MONICA, Lille	Community	urban	36-67	36-66	590	580	585	579	585	579			
281	France	1995-1997	MONICA, Strasbourg	Subnational	both	35-64	35-64	522	523	514	507	514	507			
282	France	1999-2001	The Three City Study	Community	urban	65+	65+	3,444	5,263	3,440	5,261	3,440	5,261			
283	France	2003-2005	The Three City Study	Community	urban	68+	68+	629	994	629	994	629	994			
284	France	2004-2006	National Monitoring of Arterial Risk in Lille (MONA LISA Lille)	Community	urban	35-75	35-75	795	787	795	787	795	787			
285	France	2005-2007	National Monitoring of Arterial Risk in Bas-Rhin (MONA LISA Bas-Rhin)	Subnational	both	35-74	35-74	777	769	777	769	777	769			
286	France	2005-2007	Monitoring National du Risque Artériel (MONA LISA study Haute-Garonne)	Subnational	both	35-74	35-74	829	797	829	797	829	797			
287	France	2006-2007	Etude Nationale Nutrition Santé	National	both	18-74	18-74	784	1,318	769	1,298	769	1,298			
288	France	2008-2010	The Three City Study	Community	urban	73+	73+	256	458	256	458	256	458			
289	France	2011-2013	Enquête LIttorale Souffle Air Biologie EnvironnemenT (ELISABET) Dunkerque	Community	both	40-64	40-64	753	785	753	785	753	785			
290	France	2011-2013	Enquête LIttorale Souffle Air Biologie EnvironnemenT (ELISABET) Lille	Community	both	40-64	40-64	751	834	751	835	751	834			
291	France	2014-2016	The Health Study on Environment, Biomonitoring, Physical Activity and Nutrition (Esteban)	National	both	18-74	18-74	924	1,127	910	1,113	910	1,113			
292	French Polynesia	2010	STEPS	National	both	18-64	18-64	815	1,075					Accutrend		
293	Gambia	1997	van der Sande et al., J Hum Hypertens 2000; 14: 489-96	Community	urban	25+	25+	351	549							
294	Georgia	2010	STEPS	National	both	18-64	18-64	952	3,190					Accutrend		
295	Georgia	2016	STEPS	National	both	18-69	18-69	788	2,179	873	2,275	767	2,162	CardioChek	CardioChek	
296	Germany	1982	MONICA, Erfurt	Community	urban	25-64	25-64	106	103							
297	Germany	1982-1984	MONICA, Chemnitz	Community	urban	25-64	25-64	264	285	254	273	253	272			
298	Germany	1982-1984	MONICA, Zwickau	Community	urban	25-64	25-64	233	252	229	258	227	250			
299	Germany	1984	German Cardiovascular Prevention Study (GCP) - National Health Survey 1984	Subnational	both	25-69	25-69	2,404	2,261	2,182	2,069	2,178	2,063			
300	Germany	1984-1985	MONICA, Berlin-Lichtenberg	Community	urban	25-64	25-64	583	614	567	603	566	599			
301	Germany	1984	MONICA, Bremen North/West	Community	urban	25-64	25-64	797	822	713	733	703	726			
302	Germany	1983-1984	MONICA, Halle County	Subnational	urban	25-64	25-64	1,073	1,135	964	1,028	950	1,012			
303	Germany	1982-1985	MONICA, Rest of Karl-Marx-Stadt County	Subnational	urban	25-64	25-64	565	629							
304	Germany	1982-1985	MONICA, Rest of DDR-MONICA	Subnational	urban	25-64	25-64	227	229	99	105	99	105			
305	Germany	1984-1985	MONICA, Augsburg	Community	both	25-64	25-64	1,895	1,868	1,918	1,922	1,817	1,825			

	Country	Data years	Survey/Study name/Citation	Level of representative-ness	Rural, urban or both	Age range as used for global analysis		Sample size as used for global analysis (Total cholesterol)		Sample size as used for global analysis (HDL cholesterol)		Sample size as used for global analysis (Non-HDL cholesterol)		Device used for measuring total cholesterol*	Device used for measuring HDL cholesterol*	Note
						Male	Female	Male	Female	Male	Female	Male	Female			
306	Germany	1984-1986	MONICA, Cottbus County	Community	urban	25-64	25-64	645	732	641	728	639	728			
307	Germany	1983-1987	MONICA, Rhein-Neckar Region	Community	urban	25-64	25-64	1,458	1,569	1,458	1,567	1,458	1,567			
308	Germany	1987-1988	MONICA, Erfurt	Community	urban	25-64	25-64	877	912	876	911	876	911			
309	Germany	1988	German Cardiovascular Prevention Study (GCP) - National Health Survey 1988	Subnational	both	25-69	25-69	2,606	2,576	2,573	2,562	2,573	2,556			
310	Germany	1988	MONICA, Berlin-Lichtenberg	Community	urban	25-64	25-64	687	709	686	709	686	709			
311	Germany	1988	MONICA, Bremen North/West	Community	urban	25-69	25-69	611	609	575	565	575	565			
312	Germany	1988	MONICA, Bremen Center/South/East	Community	urban	25-69	25-69	493	562	483	548	483	548			
313	Germany	1988	MONICA, Chemnitz	Community	urban	25-64	25-64	274	369	274	366	272	365			
314	Germany	1988	MONICA, Zwickau	Community	urban	25-64	25-64	182	241	180	236	180	235			
315	Germany	1989-1990	MONICA, Cottbus County	Community	urban	25-64	25-64	536	526	525	516	525	516			
316	Germany	1988-1989	MONICA, Halle County	Subnational	urban	25-64	25-64	911	1,143	906	1,137	902	1,135			
317	Germany	1988-1989	MONICA, Rest of Karl-Marx-Stadt County	Subnational	urban	25-64	25-64	520	587	517	587	517	586			
318	Germany	1989-1990	MONICA, Augsburg	Community	both	25-64	25-64	1,915	1,929	1,895	1,923	1,895	1,923			
319	Germany	1991-1992	MONICA, Bremen North/West	Community	urban	25-69	25-69	573	604	527	558	527	557			
320	Germany	1991-1992	MONICA, Bremen Center/South/East	Community	urban	25-69	25-69	517	523	503	508	503	508			
321	Germany	1991-1992	German Cardiovascular Prevention Study (GCP) - National Health Survey 1991	Subnational	both	25-69	25-69	2,592	2,645	2,478	2,549	2,477	2,548			
322	Germany	1991-1992	First National Examination of life conditions, Environment and Health in East Germany 1991/92	Subnational	both	25-69	25-69	1,050	1,152	950	1,042	950	1,041			
323	Germany	1991-1992	MONICA, Erfurt	Community	urban	25-64	25-64	585	571	583	570	583	570			
324	Germany	1993-1994	MONICA, Chemnitz	Community	urban	25-64	25-64	406	425	405	425	405	425			
325	Germany	1993-1994	MONICA, Zwickau	Community	urban	25-64	25-64	107	131	107	131	107	131			
326	Germany	1994-1995	MONICA, Augsburg	Community	both	25-64	25-64	1,872	1,908	1,863	1,908	1,863	1,908			
327	Germany	1997-1999	German National Health Interview and Examination Survey (GNHIES98)	National	both	18-79	18-79	3,278	3,442	3,274	3,442	3,274	3,442			
328	Germany	1997-2001	Study of Health in Pomerania (SHIP-0) baseline study	Subnational	both	20-80	20-80	2,104	2,182	2,099	2,175	2,098	2,175			
329	Germany	1999-2001	KORA S4 Study: Kooperative Research in the Region of Augsburg Survey 4	Community	both	24-75	24-75	2,069	2,132	2,065	2,130	2,065	2,130			
330	Germany	2000-2002	Epidemiological study of the chances of prevention, early recognition and optimal treatment of chronic diseases in an elderly population (ESTHER)	Subnational	both	50-75	50-75	4,340	5,270	2,793	3,216	2,769	3,192			
331	Germany	2002	Echinoccoccus Multilocularis and Internal Diseases in Leutkirch	Community	urban	20-65	20-65	827	931	827	931	827	931			
332	Germany	2000-2003	Heinz Nixdorf Recall Study	Subnational	urban	45-76	45-76	2,382	2,404	2,380	2,403	2,379	2,403			
333	Germany	2002-2006	Study of Health in Pomerania (SHIP-1) 5-year follow-up	Subnational	both	25-85	25-85	1,586	1,701							9
334	Germany	2005-2008	Heinz Nixdorf Recall Study	Subnational	both	50-80	50-80	2,045	2,082	2,045	2,080	2,045	2,080			
335	Germany	2006-2008	KORA F4 Study: Kooperative Research in the Region of Augsburg Follow-up of Survey 4	Community	both	31-81	31-81	1,479	1,580	1,479	1,579	1,479	1,579			
336	Germany	2008-2011	Epidemiological study of the chances of prevention, early recognition and optimal treatment of chronic diseases in an elderly population (ESTHER)	Subnational	both	58-84	58-84	2,082	2,488	2,082	2,487	2,082	2,487			
337	Germany	2008-2011	German Health Interview and Examination Survey for adults 2008-11 (DEGS1)	National	both	18-79	18-79	3,372	3,641	3,376	3,644	3,372	3,640			

	Country	Data years	Survey/Study name/Citation	Level of representative-ness	Rural, urban or both	Age range as used for global analysis		Sample size as used for global analysis (Total cholesterol)		Sample size as used for global analysis (HDL cholesterol)		Sample size as used for global analysis (Non-HDL cholesterol)		Device used for measuring total cholesterol*	Device used for measuring HDL cholesterol*	Note
						Male	Female	Male	Female	Male	Female	Male	Female			
338	Germany	2008-2012	Study of Health in Pomerania, second cohort (SHIP-TREND)	Subnational	both	20-79	20-79	2,098	2,233	2,096	2,232	2,096	2,232			
339	Germany	2011-2014	Heinz Nixdorf Recall Study	Subnational	both	55-86	55-86	1,494	1,558	1,492	1,559	1,492	1,558			
340	Ghana	2003	Women's Health Study of Accra (WHSA-I)	Community	urban		18+		874		826		800			
341	Ghana	2006	STEPS	Community	urban	25-69	25-69	342	928					Accutrend		
342	Ghana	2012-2014	Research on Obesity and Diabetes among African Migrants (RODAM), control group	Subnational	rural	25+	25+	427	665	420	658	420	658			
343	Ghana	2012-2014	Research on Obesity and Diabetes among African Migrants (RODAM), control group	Subnational	urban	25-79	25-79	416	1,031	412	1,029	412	1,029			
344	Greece	2001	Karalis et al., BMC Public Health 2007; 7: 351	Community	rural	24+	24+	91	103							
345	Greece	2013-2015	Hellenic National Nutrition and Health Survey (HNNHS)	National	both	18+	18+	413	680	412	679	412	679			
346	Greenland	2005-2010	Population Health Survey in Greenland	National	both	18+	18+	1,355	1,726	1,355	1,726	1,355	1,726			
347	Guatemala	2001-2002	CAMDI	Community	urban	20+	20+	338	626	338	626	338	626			
348	Guatemala	2003	Gregory et al., J Nutr 2007; 137: 1314-9; Site 1	Community	both	25-44	25-44	123	342							
349	Guatemala	2003	Gregory et al., J Nutr 2007; 137: 1314-9; Site 2	Community	both	25-44	25-44	295	291							
350	Guatemala	2003-2005	The Institute of Nutrition of Central America and Panama Nutrition Supplementation Trial Cohort	Community	both	25-41	25-41	193	263	197	268	187	262	Cholestech	Cholestech	
351	Guinea	2009	STEPS	Subnational	both	18-64	18-64	534	665					Accutrend		
352	Guyana	2016	STEPS	National	both	18-69	18-69	331	557	331	560	331	557			
353	Honduras	2003-2004	CAMDI	Community	urban	20+	20+	434	786	434	786	434	786			
354	Hungary	1982-1984	MONICA, Budapest	Community	urban	25-64	25-64	766	720	178	170	178	170			
355	Hungary	1982-1983	MONICA, Pecs	Community	urban	25-64	25-64	783	800	439	402	439	402			
356	Hungary	1985-1988	First Hungarian Representative Nutrition Survey	National	both	19+	19+	921	2,932	971	3,111	971	3,111			
357	Hungary	1987-1988	MONICA, Budapest	Community	urban	25-64	25-64	1,328	1,526	1,258	1,472	1,258	1,472			
358	Hungary	1987-1988	MONICA, Pecs	Community	urban	25-64	25-64	1,209	1,199	897	911	897	911			
359	Hungary	2003	The European Male Ageing Study	Community	both	40+		423		417		415				
360	Hungary	2008	The European Male Ageing Study	Community	both	40+		348		347		346				
361	Iceland	1979-1981	The Reykjavik Study (Men)	Subnational	urban	45-74		3,240								
362	Iceland	1983	MONICA, Arnes County	Community	rural	25-64	25-64	387	449	386	448	385	448			
363	Iceland	1983	MONICA, Reykjavik	Subnational	urban	25-64	25-64	435	460	433	459	433	459			
364	Iceland	1981-1984	The Reykjavik Study (Women)	Subnational	urban		46-75		3,579							
365	Iceland	1983-1985	The Reykjavik Study for the young	Subnational	urban	29-45	29-45	829	902							
366	Iceland	1985-1987	The Reykjavik Study (Men)	Subnational	urban	51-79		2,590		1,109		1,109				
367	Iceland	1988-1989	MONICA, Arnes County	Community	rural	25-64	25-64	389	438	388	437	388	437			
368	Iceland	1988-1989	MONICA, Reykjavik	Subnational	urban	25-64	25-64	413	444	413	444	413	444			
369	Iceland	1987-1991	The Reykjavik Study (Women)	Subnational	urban		52-82		3,027		3,003		3,003			
370	Iceland	1993-1994	MONICA, Arnes County	Community	rural	25-64	25-64	422	484	421	484	421	484			
371	Iceland	1993-1994	MONICA, Reykjavik	Subnational	urban	25-64	25-64	443	446	443	446	443	446			
372	Iceland	1991-1994	The Reykjavik Study (Men)	Subnational	urban	70-86		824		822		822				
373	Iceland	1994-1996	The Reykjavik Study (Women)	Subnational	urban		69-88		1,187		1,187		1,187			
374	Iceland	2001-2003	The Reykjavik Study for the young	Subnational	urban	47-62	47-62	631	714	631	714	631	714			
375	Iceland	2002-2006	AGES-Reykjavik Study	Subnational	urban	66+	66+	2,419	3,285	2,418	3,285	2,418	3,285			
376	Iceland	2005-2011	Risk Evaluation For INfarct Estimates (REFINE)	Subnational	urban	20-73	20-73	3,394	3,515	3,393	3,513	3,393	3,513			
377	Iceland	2007-2011	AGES-Reykjavik Study - follow up visit	Subnational	urban	71+	71+	1,395	1,929	1,395	1,929	1,395	1,929			
378	Iceland	2010-2012	Risk Evaluation For INfarct Estimates (REFINE) follow-up visit (REFINELO)	Subnational	urban	26-74	26-74	653	665	654	665	653	665			

	Country	Data years	Survey/Study name/Citation	Level of representative-ness	Rural, urban or both	Age range as used for global analysis		Sample size as used for global analysis (Total cholesterol)		Sample size as used for global analysis (HDL cholesterol)		Sample size as used for global analysis (Non-HDL cholesterol)		Device used for measuring total cholesterol*	Device used for measuring HDL cholesterol*	Note
						Male	Female	Male	Female	Male	Female	Male	Female			
379	Iceland	2012-2013	Risk Evaluation For INfarct Estimates (REFINE) - follow-up visit (REFLOCT)	Subnational	urban	55-73	55-73	516	560	516	560	516	560			
380	India	1995-1997	Aravind Comprehensive Eye Survey	Community	rural	40+	40+	2,267	2,783							
381	India	1997	Reddy et al., Asia Pac J Clin Nutr 2002; 11: 98-103	Subnational	both	20-59	20-59	640	480							
382	India	1996-1999	Chennai Urban Population Study	Community	urban	20+	20+	532	689	532	688	532	688			
383	India	1999-2001	Jaipur Heart Watch 2	Community	urban	20-75	20-75	518	555	518	554	518	553			
384	India	1998-2002	Vellore Birth Cohort	Subnational	both	25-31	25-31	1,154	1,053	1,155	1,054	1,152	1,052			
385	India	1999-2002	New Delhi Birth Cohort	Community	urban	26-33	26-33	869	619	869	618	869	617			
386	India	2001-2004	Chennai Urban Rural Epidemiology Study	Community	urban	20+	20+	1,096	1,252	1,093	1,252	1,093	1,251			
387	India	2003-2005	India STEPS, Ballabgarh	Subnational	rural	20-69	20-69	227	233	226	230	226	230			
388	India	2003-2005	India STEPS, Ballabgarh	Subnational	urban	20-69	20-69	214	228	214	228	214	228			
389	India	2003-2005	India STEPS, Chennai	Subnational	rural	20-69	20-69	234	231	234	231	234	231			
390	India	2003-2005	India STEPS, Chennai	Subnational	urban	20-69	20-69	217	240	217	240	216	240			
391	India	2003-2005	India STEPS, Delhi	Subnational	urban	20-69	20-69	223	211	223	211	225	211			
392	India	2003-2005	India STEPS, Dibrugarh	Subnational	rural	20-69	20-69	224	219	224	219	228	225			
393	India	2003-2005	India STEPS, Dibrugarh	Subnational	urban	20-69	20-69	224	227	224	227	223	227			
394	India	2003-2005	India STEPS, Nagpur	Subnational	rural	20-69	20-69	264	267	264	267	264	267			
395	India	2003-2005	India STEPS, Nagpur	Subnational	urban	20-69	20-69	234	242	234	242	234	242			
396	India	2006	Ramachandran et al., Diabetes Care 2008; 31: 893-8	Community	both	20+	20+	3,214	3,644	3,218	3,645	3,211	3,644			
397	India	2005-2006	Risk factor profile for chronic non-communicable diseases: Results of a community-based study in Karela, India	Community	both	18-64	18-64	494	575	494	575	494	575			
398	India	2006-2008	Central India Eye and Medical Study	Community	rural	30+	30+	2,149	2,487	2,162	2,495	2,148	2,486			
399	India	2006-2009	New Delhi Birth Cohort	Community	urban	33-38	33-38	646	439	646	439	646	439			
400	India	2008-2010	ICMR India Diabetes Study	National	both	20+	20+	1,083	981	1,069	978	1,069	977			
401	India	2012-2013	Processed and non-processed foods	National	rural	18+	18+	213	235	203	242	194	234	Cholestech	Cholestech	
402	India	2013-2014	Vellore Birth Cohort	Subnational	both	39-44	39-44	579	499	579	499	579	499			
403	India	2015-2016	Diet and nutritional status of urban population and prevalence of hypertension	National	urban	18+	18+	17,865	22,001	17,976	22,345	17,410	21,823			
404	India	2016-2019	Vellore Birth Cohort	Subnational	both	43-48	43-48	842	758	841	757	841	757			
405	Indonesia	2001	STEPS/SURKESNAS	Subnational	both	25+	25+	1,895	2,186							
406	Indonesia	2003	A genetic-ecological study of the risk foctors for lifestyle-related diseases in Oceanian populations, Study A	Community	rural	18-79	18-79	99	103	99	103	99	103			
407	Indonesia	2003	A genetic-ecological study of the risk foctors for lifestyle-related diseases in Oceanian populations, Study B	Community	rural	18-79	18-79	96	137	96	137	96	137			
408	Indonesia	2007-2008	Indonesian Family Life Surveys	National	both	40+	40+	4,687	5,555	4,688	5,633	4,343	5,385	CardioChek	CardioChek	
409	Indonesia	2013	Population Health Basic Health Research 2013 (Riskesdas 2013)	National	both	18+	18+	15,140	20,469	15,140	20,469	15,140	20,469			
410	Iran	1990-1991	National Health Survey I	National	both	20+	20+	7,016	9,323							
411	Iran	1994	Rafiei-Sarraf-Zadegan et al., East Mediterr Health J 1999; 5: 766-77	Community	urban	19-70	19-70	1,000	1,200							
412	Iran	1999-2000	National Health Survey II	Subnational	both	20+	20+	10,874	13,913	1,913	2,699	1,913	2,699			
413	Iran	1999-2001	Tehran Lipid and Glucose Study	Community	urban	18+	18+	4,470	6,212	4,461	6,199	4,460	6,199			
414	Iran	2001	Isfahan Healthy Heart Program, Arak rural	Community	rural	19-80	19-80	1,028	1,091	1,028	1,091	1,028	1,091			
415	Iran	2001	Isfahan Healthy Heart Program, Arak urban	Community	urban	19-80	19-80	2,089	2,131	2,089	2,131	2,089	2,131			

	Country	Data years	Survey/Study name/Citation	Level of representative-ness	Rural, urban or both	Age range as used for global analysis		Sample size as used for global analysis (Total cholesterol)		Sample size as used for global analysis (HDL cholesterol)		Sample size as used for global analysis (Non-HDL cholesterol)		Device used for measuring total cholesterol*	Device used for measuring HDL cholesterol*	Note
						Male	Female	Male	Female	Male	Female	Male	Female			
416	Iran	2001	Isfahan Healthy Heart Program, Isfahan rural	Community	rural	19-80	19-80	234	237	234	238	234	237			
417	Iran	2001	Isfahan Healthy Heart Program, Isfahan urban	Community	urban	19-80	19-80	1,782	1,932	1,782	1,932	1,782	1,932			
418	Iran	2001	Isfahan Healthy Heart Program, Najaf Abad rural	Community	rural	19-80	19-80	409	419	409	419	409	419			
419	Iran	2001	Isfahan Healthy Heart Program, Najaf Abad urban	Community	urban	19-80	19-79	581	578	581	578	581	578			
420	Iran	2003-2004	Childhood and Adolescence Surveillance and Prevention of Adult Noncommunicable Disease (CASPIAN)	National	both	18-18	18-18	60	77	60	77	60	77			
421	Iran	2003-2004	The Persian Gulf Healthy Heart Study	Subnational	urban	25-75	25-75	1,734	1,968	1,723	1,955	1,723	1,954			
422	Iran	2002-2005	Tehran Lipid and Glucose Study	Community	urban	18+	18+	2,177	2,904	2,167	2,896	2,167	2,896			
423	Iran	2005	Provincial Non-Communicable Disease Surveillance Survey 2005	National	both	25-64	25-64	23,538	25,072							
424	Iran	2007	Isfahan Healthy Heart Program, Arak rural	Community	rural	19-80	19-80	1,030	1,028	1,030	1,028	1,030	1,028			
425	Iran	2007	Isfahan Healthy Heart Program, Arak urban	Community	urban	19-80	19-80	1,429	1,366	1,429	1,366	1,429	1,366			
426	Iran	2007	Isfahan Healthy Heart Program, Isfahan rural	Community	rural	19-80	19-80	158	153	158	153	158	153			
427	Iran	2007	Isfahan Healthy Heart Program, Isfahan urban	Community	urban	19-80	19-80	1,415	1,436	1,415	1,436	1,415	1,436			
428	Iran	2007	Isfahan Healthy Heart Program, Najaf Abad rural	Community	rural	19-79	19-80	255	254	255	254	255	254			
429	Iran	2007	Isfahan Healthy Heart Program, Najaf Abad urban	Community	urban	19-80	19-80	498	544	498	544	498	544			
430	Iran	2007	National Non-Communicable Disease Surveillance Survey 2007	National	both	25-64	25-64	1,449	1,549	1,433	1,539	1,432	1,539			
431	Iran	2007	Provincial Non-Communicable Disease Surveillance Survey 2007	National	both	25-64	25-64	9,031	9,714	9,027	9,735	8,982	9,677			
432	Iran	2005-2008	Tehran Lipid and Glucose Study	Community	urban	18+	18+	2,540	3,360	2,538	3,357	2,538	3,356			
433	Iran	2008-2010	Amol county study	Community	rural	18+	18+	1,676	1,017	1,676	1,017	1,676	1,017			
434	Iran	2008-2010	Amol county study	Community	urban	18+	18+	1,515	1,468	1,422	1,384	1,421	1,384			
435	Iran	2008-2010	Tehran city study	Community	urban	20+	20+	388	500	385	499	385	499			
436	Iran	2008-2009	Zahedan city study	Community	urban	20+	20+	1,069	1,009	1,069	1,009	1,069	1,009			
437	Iran	2009-2010	Childhood and Adolescence Surveillance and Prevention of Adult Noncommunicable Disease (CASPIAN)	National	both	18-18	18-18	426	428	348	340	346	337			
438	Iran	2009-2010	The Persian Gulf Healthy Heart Study	Subnational	urban	31-79	31-79	832	1,012	833	1,011	831	1,011			
439	Iran	2008-2011	Tehran Lipid and Glucose Study	Community	urban	20+	20+	4,722	6,038	4,718	6,037	4,718	6,037			
440	Iran	2010-2012	Golestan Cohort Study Second Phase	Subnational	rural	43-82	43-82	4,310	4,900	4,297	4,895	4,294	4,886			
441	Iran	2010-2012	Golestan Cohort Study Second Phase	Community	urban	43-82	43-82	1,082	1,056	1,083	1,056	1,081	1,055			
442	Iran	2011	Provincial Non-Communicable Disease Surveillance Survey 2011	National	both	25-69	25-69	1,975	3,263	1,973	3,263	1,972	3,258			
443	Iran	2012-2014	Pars Cohort Study	Community	rural	40-90	40-90	4,268	4,981	4,267	4,980	4,267	4,980			
444	Iran	2013-2014	Bushehr Elderly Health Program (BEH)	Community	urban	60+	60+	1,451	1,543	1,452	1,544	1,450	1,543			
445	Iran	2014-2015	Childhood and Adolescence Surveillance and Prevention of Adult Noncommunicable Disease (CASPIAN)	National	both	18-18	18-18	88	88	88	88	88	88			
446	Iran	2014-2016	The PERSIAN Fasa Cohort Study	Community	both	35-70	35-70	4,414	5,337	4,416	5,339	4,413	5,337			
447	Iran	2014-2016	The PERSIAN Guilan Cohort Study	Community	both	35-70	35-70	4,873	5,607	4,874	5,607	4,873	5,607			
448	Iran	2014-2016	The PERSIAN Kermanshah Cohort Study	Community	both	35-69	35-69	4,725	5,149	4,727	5,150	4,724	5,149			
449	Iran	2014-2016	The PERSIAN Kharameh Cohort Study	Community	both	35-70	35-70	4,688	5,821	4,683	5,815	4,682	5,812			
450	Iran	2014-2016	The PERSIAN Tabriz Cohort Study	Community	both	35-70	35-70	6,639	8,140	6,640	8,141	6,639	8,138			
451	Iran	2016	Iran STEPS 2016	National	both	25+	25+	9,009	10,372	9,008	10,367	8,999	10,361			
452	Iran	2016-2018	The PERSIAN BandarKong Cohort Study	Community	both	35-70	35-70	1,700	2,257	1,701	2,256	1,700	2,256			

	Country	Data years	Survey/Study name/Citation	Level of representative-ness	Rural, urban or both	Age range as used for global analysis		Sample size as used for global analysis (Total cholesterol)		Sample size as used for global analysis (HDL cholesterol)		Sample size as used for global analysis (Non-HDL cholesterol)		Device used for measuring total cholesterol*	Device used for measuring HDL cholesterol*	Note
						Male	Female	Male	Female	Male	Female	Male	Female			
453	Iran	2016-2017	IraPEN Study	Community	rural	30+	30+	2,788	3,097					LipidPro		
454	Iran	2016-2018	The PERSIAN Urmia Cohort Study	Community	both	35-70	35-70	2,188	2,834	2,188	2,831	2,188	2,831			
455	Iran	2016-2019	The PERSIAN Ardabil Cohort Study	Community	both	35-70	35-70	5,354	6,514	5,355	6,514	5,354	6,514			
456	Iran	2018-2019	Prevalence of risk factors for cardiovascular disease among a rural population in eastern Iran	Subnational	rural	18-79	18+	152	145							
457	Iran	2017-2018	The PERSIAN Kavar Cohort Study	Community	urban	35-70	35-70	2,386	2,506	2,373	2,479	2,372	2,477			
458	Iran	2017-2019	The PERSIAN Mashhad Cohort Study	Community	both	35-70	35-69	2,257	2,603	2,255	2,604	2,255	2,603			
459	Iran	2016-2019	The PERSIAN Shahrekord Cohort Study	Community	both	35-70	35-70	4,273	4,532	4,274	4,531	4,272	4,531			
460	Iraq	2005-2006	STEPS	National	both	25-64	25-64	1,819	2,381							
461	Iraq	2015	STEPS	National	both	18+	18+	1,510	2,198	1,517	2,199	1,505	2,189			
462	Ireland	1998	Survey of Lifestyle, Attitudes and Nutritional in Ireland 1998	National	both	18+	18+	120	277	113	264	113	264			
463	Ireland	2002	Survey of Lifestyle, Attitudes and Nutritional in Ireland 2002	National	both	18-79	18+	153	191	153	191	153	191			
464	Ireland	2006-2007	Survey of Lifestyle, Attitudes and Nutritional in Ireland 2006-2007	National	both	45-79	45-79	514	648	511	648	511	647			
465	Ireland	2008-2010	National Adult Nutrition Survey	National	both	18+	18+	569	562	567	556	567	556			
466	Ireland	2009-2011	The Irish Longitudinal Study on Ageing	National	both	50+	50+	2,606	3,017	2,607	3,017	2,606	3,017			
467	Israel	1985-1986	MONICA, Tel Aviv	Community	urban	25-64	25-64	391	361	384	359	384	359			
468	Israel	1990-1991	The Jerusalem Longitudinal Cohort Study	Community	urban	69-70	69-70	245	207							
469	Israel	1997-1998	The Jerusalem Longitudinal Cohort Study	Community	urban	76-77	76-77	248	234	243	232	242	231			
470	Israel	1999-2005	The Israel Glucose Intolerance, Obesity and Hypertention Study	National	urban	58+	58+	497	477	493	474	493	474			
471	Israel	2002-2009	Hadera District Study	Subnational	urban	25-78	25-78	383	374	382	374	382	374			
472	Israel	2005-2006	The Jerusalem Longitudinal Cohort Study	Community	urban	83-85	83-85	312	385	309	380	308	380			
473	Italy	1981	Gualandri et al., Metabolism 1985; 34: 212-21	Community	urban	25-84	25-84	271	302							
474	Italy	1982-1987	MONICA, Latina	Community	both	24-66	24-66	845	861	846	858	845	858			
475	Italy	1985	Finland, Italy, Netherlands, Elderly (Fine-Italy	Community	rural	65-84		680		679		679				
476	Italy	1986	MONICA, Friuli	Subnational	urban	25-64	25-64	924	915	920	905	920	904			
477	Italy	1987	Palli et al., Eur J Nutr 1999; 38: 90-8	Subnational	urban	30-64	30-64	331	245							
478	Italy	1986-1987	MONICA-Brianza survey	Subnational	urban	25-64	25-64	816	831	816	832	815	830			
479	Italy	1989	MONICA, Friuli	Subnational	urban	25-64	25-64	895	896	893	896	890	893			
480	Italy	1989	Ventimiglia Heart Study	Community	rural	20+	20+	482	587	482	586	480	586			
481	Italy	1990	Bruneck Study	Community	rural	40-79	40-79	468	450	468	450	468	450			
482	Italy	1983-1996	Malattie cardiovascolari ATerosclerotiche Istituto Superiore di Sanità	Community	rural	18-77	18-77	3,954	4,475	3,951	4,473	3,950	4,472			
483	Italy	1989-1990	MONICA-Brianza survey	Subnational	urban	25-64	25-64	794	786	795	786	793	786			
484	Italy	1992-1993	Italian Longitudinal Study on Aging	National	both	65-84	65-84	1,713	1,580	1,717	1,566	1,697	1,560			
485	Italy	1994	MONICA, Friuli	Subnational	urban	25-64	25-64	881	887	880	886	880	885			
486	Italy	1993-1994	MONICA-Brianza survey	Subnational	urban	25-64	25-64	808	864	808	863	807	863			
487	Italy	1995	Vobarno Study	Community	both	35-64	35-64	265	309							
488	Italy	1995	Bruneck Study	Community	rural	45-84	45-84	412	412	412	412	412	412			
489	Italy	1995-1996	Italian Longitudinal Study on Aging	National	both	68-90	68-90	1,049	925	1,025	902	1,023	902			
490	Italy	1995-1999	PROgetto Veneto Anziani (PROVA)	Subnational	both	65+	65+	1,225	1,832	1,222	1,824	1,218	1,824			
491	Italy	1999	InCHIANTI; Antonelli-Incalzi et al., Atherosclerosis 2006; 186: 200-6	Community	both	25+	25+	582	725							
492	Italy	1998-1999	progetto VIP	Community	both	25-74	25-74	582	589	564	560	563	559			

	Country	Data years	Survey/Study name/Citation	Level of representative-ness	Rural, urban or both	Age range as used for global analysis		Sample size as used for global analysis (Total cholesterol)		Sample size as used for global analysis (HDL cholesterol)		Sample size as used for global analysis (Non-HDL cholesterol)		Device used for measuring total cholesterol*	Device used for measuring HDL cholesterol*	Note
						Male	Female	Male	Female	Male	Female	Male	Female			
493	Italy	2000	Bruneck Study	Community	rural	50-89	50-89	331	361	331	361	331	361			
494	Italy	1998-2002	Osservatorio Epidemiologico Cardiovascolare	National	both	35-74	35-74	4,831	4,705	4,836	4,711	4,828	4,704			
495	Italy	2000-2001	Italian Longitudinal Study on Aging	National	both	73-93	73-93	695	681	684	672	683	672			
496	Italy	2002	Vobarno Study; Muiesan et al., Blood Press 2006; 15: 14-9	Community	both	35-74	35-74	168	212							
497	Italy	2001-2003	The Study of Asti	Community	both	45-64	45-64	780	878	780	878	780	878			
498	Italy	2000-2003	PROgetto Veneto Anziani (PROVA)	Subnational	both	67+	67+	789	1,334	658	1,113	657	1,111			
499	Italy	2003	The European Male Ageing Study	Community	both	40+		433		433		433				
500	Italy	2002-2005	PROgetto Veneto Anziani (PROVA)	Subnational	both	70+	70+	557	1,028	559	1,023	557	1,022			
501	Italy	2005	Bruneck Study	Community	rural	55-93	55-93	264	307	264	307	264	307			
502	Italy	2004-2005	Italian Project on the Epidemiology of Alzheimer's Disease	National	both	65-84	65-84	1,349	1,196							
503	Italy	2004-2005	Vobarno study	Community	rural	55-74	55-74	99	112	96	110	96	110			
504	Italy	2005-2007	Moli-family Study	Subnational	both	18+	18+	216	270	216	270	216	270			
505	Italy	2008	The European Male Ageing Study	Community	both	40+		288		289		288				
506	Italy	2005-2010	Moli-sani Study	Subnational	both	35+	35+	11,621	12,550	11,620	12,551	11,618	12,549			
507	Italy	2008-2009	progetto VIP	Community	both	25-74	25-74	600	599	600	599	600	599			
508	Italy	2010	Bruneck Study	Community	rural	60-98	60-98	225	259	225	259	225	259			
509	Italy	2009-2010	Grosso et al., J Epidemiol. 2014; 24: 327-33	Community	both	19+	19+	760	1,129	760	1,129	760	1,129			
510	Italy	2008-2012	Osservatorio Epidemiologico Cardiovascolare/Health Examination Survey	National	both	35-80	35-80	4,331	4,302	4,332	4,301	4,330	4,301			
511	Italy	2010-2012	CArdiovascular risk MEtabolic syndrome LIver and Autoimmunity diseases (CA.ME.LI.A)	Community	both	18-75	18-75	477	514	476	515	476	514			
512	Italy	2011-2012	Vobarno study	Community	rural	49-62	49-62	107	142	106	142	106	142			
513	Italy	2015	Bruneck Study	Community	rural	65-98	65-98	168	163	168	163	168	163			
514	Italy	2016	EVA Tyrol Study South Italy	Subnational	both	18-18		2		2		2				
515	Italy	2018-2019	progetto VIP	Community	both	25-74	25-74	600	598	599	598	599	598			
516	Jamaica	2000-2001	Jamaica Health and Lifestyle Survey	National	both	18-74	18-74	362	865					Accutrend		
517	Jamaica	2006-2007	Jamaica Youth Risk and Resiliency Behaviour Survey 2006	National	both	18-19	18-19	5	21					Accutrend		
518	Jamaica	2007-2008	Jamaica Health and Lifestyle Survey	National	both	18-74	18-74	442	1,207					Accutrend		
519	Jamaica	2012	Older Persons in Jamaica 2012	National	both	60+	60+	158	206	158	206	158	206			5
520	Japan	1980	APCSC-Hisayama	Community	urban	20+	20+	5,018	3,760							
521	Japan	1980	National Cardiovascular Survey	National	both	30+	30+	4,689	5,973							
522	Japan	1981	APCSC-Hisayama	Community	urban	40-69	40-69	820	1,074							
523	Japan	1980-1983	Aito Town Study	Community	rural	20-77	20-77	725	946	442	449	442	449			
524	Japan	1985-1986	Akabane Study	Community	urban	40-69	40-69	812	1,022	812	1,022	812	1,022			
525	Japan	1987	Konan Town Study	Community	rural	20-79	20-79	69	88	69	88	69	88			
526	Japan	1988	Konan Town Study	Community	rural	20-79	20-79	76	85	76	85	76	85			
527	Japan	1989	Konan Town Study	Community	rural	20-79	20-79	59	63	59	63	59	63			
528	Japan	1988-1990	Miyama Cohort Study	Community	rural	40-80	40-80	157	256	153	255	153	255			
529	Japan	1989	National Nutrition Survey	National	both	30+	30+	2,706	4,037	2,704	4,036	2,703	4,036			
530	Japan	1990	Serum Lipid Survey; Yamamoto et al., J Atheroscler Thromb 2003; 10: 176-85	National	both	20+	20+	17,424	11,917							
531	Japan	1990	Konan Town Study	Community	rural	20-79	20-79	30	58	30	58	30	58			
532	Japan	1990	National Nutrition Survey and National Cardiovascular Survey	National	both	30+	30+	3,303	4,591	3,301	4,591	3,301	4,591			

	Country	Data years	Survey/Study name/Citation	Level of representative-ness	Rural, urban or both	Age range as used for global analysis		Sample size as used for global analysis (Total cholesterol)		Sample size as used for global analysis (HDL cholesterol)		Sample size as used for global analysis (Non-HDL cholesterol)		Device used for measuring total cholesterol*	Device used for measuring HDL cholesterol*	Note
						Male	Female	Male	Female	Male	Female	Male	Female			
533	Japan	1991	Konan Town Study	Community	rural	20-79	20-79	93	117	93	117	93	117			
534	Japan	1991	Shigaraki Town Study	Community	rural	30-89	30-89	233	330	233	330	233	330			
535	Japan	1991	National Nutrition Survey	National	both	20+	20+	3,386	4,761	3,385	4,760	3,385	4,760			
536	Japan	1992	Konan Town Study	Community	rural	20-79	20-79	55	52	55	52	55	52			
537	Japan	1992	Shigaraki Town Study	Community	rural	30-89	30-89	288	387	288	387	288	387			
538	Japan	1990-1994	Japan Public Health Center-based prospective Study (JPHC Study), Cohort I	Subnational	both	40-59	40-59	8,762	14,504	2,856	3,708	2,851	3,702			
539	Japan	1992	National Nutrition Survey	National	both	30+	30+	2,838	4,086	2,838	4,086	2,838	4,086			
540	Japan	1993	Fukuda et al., Hypertens Res 2002; 25: 179-84	Community	rural	35+	35-84	669	1,228							
541	Japan	1993	Konan Town Study	Community	rural	20-79	20-79	54	65	54	65	54	65			
542	Japan	1993	Shigaraki Town Study	Community	rural	30-89	30-89	300	453	300	453	300	453			
543	Japan	1993	National Nutrition Survey	National	both	30+	30+	2,559	3,791	2,560	3,791	2,559	3,791			
544	Japan	1993-1994	Japan Public Health Center-based prospective Study (JPHC Study), Cohort II	Subnational	both	40-69	40-69	8,557	16,214	8,549	16,207	8,549	16,206			
545	Japan	1994	Konan Town Study	Community	rural	20-79	20-79	42	59	42	59	42	59			
546	Japan	1994	Shigaraki Town Study	Community	rural	30-89	30-89	251	336	251	336	251	336			
547	Japan	1994	National Nutrition Survey	National	both	20-59	20-59	1,785	2,811	1,785	2,811	1,785	2,811			
548	Japan	1995	Konan Town Study	Community	rural	20-79	20-79	45	60	45	60	45	60			
549	Japan	1995	Shigaraki Town Study	Community	rural	30-89	30-89	297	470	297	470	297	470			
550	Japan	1995	National Nutrition Survey	National	both	20-59	20-59	1,710	2,775	1,710	2,775	1,710	2,775			
551	Japan	1996	Shigaraki Town Study	Community	rural	30-79	30-89	85	152	85	152	85	152			
552	Japan	1996	National Nutrition Survey	National	both	30+	30+	2,215	3,423	2,215	3,422	2,215	3,422			
553	Japan	1997	Mannami et al., Stroke 2000; 31: 2958-65	Community	urban	30-89	30-89	2,033	2,354							
554	Japan	1997	Shigaraki Town Study	Community	rural	30-79	30-89	61	100	61	100	61	100			
555	Japan	1997	National Nutrition Survey	National	both	20+	20+	2,517	3,807	2,516	3,807	2,516	3,807			
556	Japan	1998	Niigata Study	Community	urban	70-70	70-70	304	290							
557	Japan	1998	National Nutrition Survey	National	both	20+	20+	2,643	3,928	2,642	3,928	2,642	3,928			
558	Japan	1999	Niigata Study	Community	urban	71-71	71-71	242	214	242	214	242	214			
559	Japan	1999	National Nutrition Survey	National	both	20+	20+	2,061	3,205	2,060	3,205	2,060	3,205			
560	Japan	2000	Niigata Study	Community	urban	72-72	72-72	233	199	233	199	233	199			
561	Japan	2000	National Nutrition Survey and National Cardiovascular Survey	National	both	20+	20+	2,285	3,280	2,285	3,280	2,284	3,280			
562	Japan	2001	The Japan Association of Health Service Database	Subnational	both	20+	20+	1,173,802	1,022,217	1,173,802	1,022,217	1,173,802	1,022,217			
563	Japan	2001	Niigata Study	Community	urban	73-73	73-73	235	199	235	199	235	199			
564	Japan	2001	National Nutrition Survey	National	both	20+	20+	2,132	3,359	2,131	3,359	2,131	3,359			
565	Japan	2002	Niigata Study	Community	urban	74-74	74-74	226	201	226	201	226	201			
566	Japan	2002	National Nutrition Survey	National	both	20+	20+	2,129	3,154	2,128	3,154	2,128	3,154			
567	Japan	2002-2003	The Hisayama Study	Community	rural	40+	40+	1,413	1,883	1,413	1,883	1,413	1,883			
568	Japan	2003	National Health and Nutrition Survey	National	both	20+	20+	2,108	3,170	2,107	3,169	2,107	3,169			
569	Japan	2003	Niigata Study	Community	urban	75-75	75-75	215	191	215	191	215	191			
570	Japan	2004	National Health and Nutrition Survey	National	both	20+	20+	1,548	2,367	1,547	2,367	1,547	2,367			
571	Japan	2004	Niigata Study	Community	urban	76-76	76-76	213	184	213	184	213	184			
572	Japan	2005	Kobayashi et al., Circ J 2007; 71: 1734-7	Subnational	urban	25+	25+	7,339	14,552							
573	Japan	2005	National Health and Nutrition Survey	National	both	20+	20+	1,557	2,298	1,557	2,298	1,557	2,298			
574	Japan	2005	Niigata Study	Community	urban	77-77	77-77	202	189	202	189	202	189			
575	Japan	2006	National Health and Nutrition Survey	National	both	20+	20+	1,753	2,549	1,753	2,549	1,753	2,549			
576	Japan	2006	Niigata Study	Community	urban	78-78	78-78	195	195	195	195	195	195			

	Country	Data years	Survey/Study name/Citation	Level of representative-ness	Rural, urban or both	Age range as used for global analysis		Sample size as used for global analysis (Total cholesterol)		Sample size as used for global analysis (HDL cholesterol)		Sample size as used for global analysis (Non-HDL cholesterol)		Device used for measuring total cholesterol*	Device used for measuring HDL cholesterol*	Note
						Male	Female	Male	Female	Male	Female	Male	Female			
577	Japan	2007	National Health and Nutrition Survey	National	both	20+	20+	1,625	2,376	1,625	2,376	1,625	2,376			
578	Japan	2007	Niigata Study	Community	urban	79-79	79-79	182	193	182	193	182	193			
579	Japan	2008	Kobayashi J, 2008	Community	both	40+	40+	6,562	11,944							
580	Japan	2008	National Health and Nutrition Survey	National	both	20+	20+	1,818	2,620	1,817	2,620	1,817	2,620			
581	Japan	2008	Niigata Study	Community	urban	80-80	80-80	169	176	169	176	169	176			
582	Japan	2009	National Health and Nutrition Survey	National	both	20+	20+	1,736	2,538	1,735	2,538	1,735	2,538			
583	Japan	2010	National Health and Nutrition Survey	National	both	20+	20+	1,597	2,254	1,597	2,254	1,597	2,254			
584	Japan	2011	National Health and Nutrition Survey	National	both	20+	20+	1,463	2,082	1,463	2,082	1,463	2,082			
585	Japan	2011	The Tokyo Health Service Association Database	Community	urban	20+	20+	39,350	18,888	69,584	45,216	39,350	18,888			
586	Japan	2012	National Health and Nutrition Survey	National	both	20+	20+	5,766	8,314	5,766	8,314	5,766	8,314			
587	Japan	2013	National Health and Nutrition Survey	National	both	20+	20+	1,382	1,905	1,382	1,905	1,382	1,905			
588	Japan	2012-2016	The Nagahama Study	Community	rural	35-79	35-79	3,206	6,619	3,206	6,619	3,206	6,619			
589	Japan	2014	National Health and Nutrition Survey	National	both	20+	20+	1,473	2,020	1,473	2,020	1,473	2,020			
590	Japan	2015	National Health and Nutrition Survey	National	both	20+	20+	1,336	1,966	1,336	1,966	1,336	1,966			
591	Japan	2016	National Health and Nutrition Survey	National	both	20+	20+	4,655	6,696	4,655	6,696	4,655	6,696			
592	Japan	2017	National Health and Nutrition Survey	National	both	20+	20+	1,212	1,695	1,211	1,695	1,211	1,695			
593	Jordan	1995	Jaddou et al., J Hum Hypertens 1996; 10: 815-21	Community	urban	25+	25+	839	1,434							
594	Jordan	2004	Behavioural Risk Factor Surveillence Survey	National	rural	18+	18+	235	465	235	466	235	463			
595	Jordan	2007	Behavioural Risk Factor Surveillence Survey	National	both	18+	18+	331	431	330	432	329	431			
596	Jordan	2009	Metablic abnomalities and vitamin D study	National	both	18+	18+	1,141	3,349	1,134	3,343	1,133	3,342			
597	Jordan	2016-2017	National Cardiovascular Diseases and Diabetes Study (NCDDS)	National	both	18+	18+	1,182	2,735	1,179	2,734	1,178	2,734			
598	Kazakhstan	2015	Almaty STEPS	Subnational	both	18-69	18-69	382	1,145	381	1,145	381	1,145			
599	Kazakhstan	2015	Shymkent STEPS	Subnational	both	18-69	18-69	349	803	360	830	343	795			
600	Kazakhstan	2015-2016	Aktobe STEPS	Subnational	both	18-69	18-69	334	1,143	335	1,143	330	1,138			
601	Kenya	2015	STEPS	National	both	18-69	18-69	1,103	1,905	1,585	2,327	1,065	1,859	CardioChek	CardioChek	
602	Kiribati	1981	Epidemiological survey of Kiribati	Subnational	rural	20+	20+	467	522							
603	Kiribati	1981	Epidemiological survey of Kiribati	Subnational	urban	20+	20+	915	875							
604	Kiribati	2004	STEPS	National	both	20-64	18-64	266	437					Accutrend		
605	Kiribati	2015-2016	STEPS	National	both	18-69	18-69	374	534	402	524	311	455	CardioChek	CardioChek	
606	Kuwait	2006	STEPS	National	both	20-64	20-64	914	1,296	912	1,296	912	1,296			
607	Kuwait	2011-2014	Kuwait Diabetes Epidemiology Program	National	urban	18+	18+	2,781	2,148	2,772	2,146	2,772	2,146			
608	Kuwait	2014	STEPS	National	both	18-69	18-69	995	1,619	993	1,617	993	1,617			
609	Kyrgyzstan	2013	STEPS	National	both	25-64	25-64	881	1,543					CardioChek		
610	Lao PDR	2013	STEPS	National	both	18-64	18-64	834	1,368	947	1,426	822	1,353	CardioChek	CardioChek	
611	Latvia	2008-2009	Cardiovascular risk factor study	National	both	25-74	25-74	1,368	2,413	1,368	2,413	1,365	2,411			
612	Lebanon	2017	STEPS	National	both	18-69	18-69	446	747	446	747	446	747			
613	Lesotho	2012	STEPS	National	both	25-64	25-64	463	1,114	659	1,287	431	1,053	CardioChek	CardioChek	
614	Libya	1999	Kadiki et al., Diabetes Metab 2001; 27: 647-54	Community	both	25-84	25-84	182	334							
615	Libya	1999	Buysschaert et al., Diabetes Metab 2001; 27: 655-9	Community	urban	25-74	25-74	150	241							
616	Libya	2009	STEPS	National	both	25-64	25-64	857	668					Accutrend		
617	Lithuania	1983-1985	MONICA, Kaunas	Community	urban	35-64	35-64	727	735	667	625	666	625			
618	Lithuania	1987	Countrywide Integrated Noncommunicable Diseases Intervention Programme survey	Subnational	rural	25-64	25-64	963	1,075	887	1,019	886	1,019			
619	Lithuania	1986-1987	MONICA, Kaunas	Community	urban	35-64	35-64	848	840	814	808	814	808			
620	Lithuania	1992-1993	MONICA, Kaunas	Community	urban	35-64	35-64	596	613	572	583	571	582			

	Country	Data years	Survey/Study name/Citation	Level of representative-ness	Rural, urban or both	Age range as used for global analysis		Sample size as used for global analysis (Total cholesterol)		Sample size as used for global analysis (HDL cholesterol)		Sample size as used for global analysis (Non-HDL cholesterol)		Device used for measuring total cholesterol*	Device used for measuring HDL cholesterol*	Note
						Male	Female	Male	Female	Male	Female	Male	Female			
621	Lithuania	1992-1993	Countrywide Integrated Noncommunicable Diseases Intervention Programme survey	Subnational	rural	25-64	25-64	645	820	552	712	551	712			
622	Lithuania	1998-1999	Countrywide Integrated Noncommunicable Diseases Intervention Programme survey	Subnational	rural	25-64	25-64	784	975	769	949	769	949			
623	Lithuania	2001-2002	MONICA4	Community	urban	35-64	35-64	622	776	603	723	603	723			
624	Lithuania	2006-2007	Countrywide Integrated Noncommunicable Diseases Intervention Programme survey	Subnational	rural	25-64	25-64	718	971	718	972	718	971			
625	Lithuania	2006-2008	MONICA4 Follow-up	Community	urban	45-69	45-69	317	424	314	415	314	415			
626	Luxembourg	2007-2009	Observation des Risques et de la Santé Cardio-Vasculaire au Luxembourg (ORISCAV-LUX)	National	both	18-69	18-69	696	731	696	731	696	731			
627	Malawi	2009	Malawi Longitudinal Study of Families and Health (MLSFH)	Subnational	rural	18+	18+	274	480	274	480	274	480			
628	Malawi	2009	STEPS	National	both	25-64	25-64	538	1,222					Accutrend		
629	Malawi	2017	STEPS	National	both	18-69	18-69	985	2,018					CardioChek		
630	Malaysia	1996	National Health and Morbidity Survey (NHMS)	National	both	30+	20+	6,861	8,379					Accutrend		
631	Malaysia	2004	Rampal et al., Public Health 2008; 122: 11-8	National	both	18+	18+	4,050	6,326	4,050	6,326	4,050	6,326			
632	Malaysia	2006	National Health and Morbidity Survey (NHMS)	National	both	18+	18+	9,134	12,506					Accutrend		
633	Malaysia	2008	Metabolic Syndrome Study in Malaysia	National	rural	18+	18+	736	1,334	737	1,334	734	1,333			
634	Malaysia	2008	Metabolic Syndrome Study in Malaysia	National	urban	18+	18+	747	1,416	740	1,406	740	1,401			
635	Malaysia	2011	National Health and Morbidity Survey (NHMS)	National	both	18+	18+	7,480	8,696					CardioChek		
636	Malaysia	2015	National Health and Morbidity Survey (NHMS)	National	both	18+	18+	8,794	9,534							
637	Malta	1984	MONICA, Malta	Community	urban	25-64	25-64	657	635	654	637	652	631			
638	Marshall Islands	2002	STEPS	National	both	18-64	18-64	381	531	376	529	376	529			
639	Mauritania	2006	STEPS	Community	urban	18-64	18-64	1,036	1,187							
640	Mauritius	1987	Mauritius non communicable disease survey	National	both	25-74	25-74	2,326	2,639	2,335	2,649	2,319	2,636			
641	Mauritius	1992	Mauritius non communicable disease survey	National	both	25-74	25-74	2,988	3,480	2,976	3,474	2,975	3,474			
642	Mauritius	1998	Mauritius non communicable disease survey	National	both	25-74	25-74	2,560	3,245							
643	Mauritius	2009	Mauritius non communicable disease survey	National	both	20-74	20-74	2,889	3,424	2,883	3,425	2,881	3,424			
644	Mexico	1988	Encuesta Nacional de Seroepidemiologia	National	both	18+	18+	7,014	12,541							
645	Mexico	1990-1992	Mexico City Diabetes Study	Community	urban	30-69	30-79	941	1,341	929	1,335	929	1,335			
646	Mexico	1992-1993	Encuesta Nacional de Enfermedades Cronicas	National	urban	20-69	20-69	5,700	7,724	5,672	7,714	5,668	7,709			
647	Mexico	1993-1995	Mexico City Diabetes Study	Community	urban	34-69	34-79	707	1,034	688	1,029	688	1,029			
648	Mexico	1997-1999	Mexico City Diabetes Study	Community	urban	37-79	40-79	701	982	683	963	683	963			
649	Mexico	2004-2005	CArdiovascular Risk factors Multiple Evaluation in Latin America (CARMELA)	Community	urban	25-64	25-64	833	889	833	889	833	889			
650	Mexico	2006	Encuesta Nacional de Salud y Nutricion	National	both	20+	20+	3,168	5,066	3,740	5,935	3,151	5,045			
651	Mexico	2006	PREVENIMSS National Coverage Surveys	Subnational	both	20+	20+	6,030	7,632					Accutrend		
652	Mexico	2007-2009	Mexico City Diabetes Study	Community	urban	51+	51+	460	711							
653	Mexico	2009-2012	Encuesta Nacional Sobre Niveles de vida de los Hogares	National	both	18+	18+	2,811	3,925	3,916	4,816	2,752	3,878	CardioChek	CardioChek	
654	Mexico	2011-2012	Encuesta Nacional de Salud y Nutricion	National	both	20+	20+	4,146	6,249	4,144	6,243	4,142	6,240			
655	Mexico	2012	The Mexican Health and Aging Study	National	both	50+	50+	770	1,082	770	1,082	770	1,082			
656	Mexico	2016	Encuesta Nacional de Salud y Nutricion	National	both	20+	20+	1,373	2,597	1,366	2,595	1,366	2,595			
657	Micronesia (Federated States of)	2002	STEPS	Subnational	both	25-64	25-64	274	419	258	388	258	384			
658	Micronesia (Federated States of)	2006	STEPS	Subnational	both	20-64	20-64	144	389					Accutrend		
659	Micronesia (Federated States of)	2008	STEPS	Subnational	both	25-64	25-64	278	449					Accutrend		
660	Micronesia (Federated States of)	2009	STEPS	Subnational	both	18-64	18-64	210	295					Accutrend		

	Country	Data years	Survey/Study name/Citation	Level of representative-ness	Rural, urban or both	Age range as used for global analysis		Sample size as used for global analysis (Total cholesterol)		Sample size as used for global analysis (HDL cholesterol)		Sample size as used for global analysis (Non-HDL cholesterol)		Device used for measuring total cholesterol*	Device used for measuring HDL cholesterol*	Note
						Male	Female	Male	Female	Male	Female	Male	Female			
661	Micronesia (Federated States of)	2016	STEPS	Subnational	both	18-69	18-69	468	763					CardioChek		
662	Moldova	2013	STEPS	National	both	18-69	18-69	1,313	2,305	1,254	2,250	1,215	2,201	CardioChek	CardioChek	
663	Mongolia	2005	STEPS	National	both	20-64	18-64	322	348					Accutrend		
664	Mongolia	2009	STEPS	National	both	18-64	18-64	520	751	706	995	506	731	Accutrend		
665	Mongolia	2013	STEPS	National	both	18-64	18-64	801	1,057	882	1,085	791	1,033	Multicare		
666	Morocco	2017	STEPS	National	both	18+	18+	1,133	2,413	1,517	2,825	1,120	2,383	CardioChek	CardioChek	
667	Mozambique	2014-2015	STEPS	National	both	18-64	18-64	545	977	649	1,049	501	912	CardioChek	CardioChek	
668	Myanmar	2003-2004	STEPS	Subnational	both	25-74	25-74	1,917	2,342	1,905	2,338	1,904	2,338			
669	Myanmar	2014	STEPS	National	both	25-64	25-64	2,797	5,317	2,611	5,222	2,517	5,148	LipidoCare	LipidoCare	
670	Myanmar	2013-2014	STEPS, Yangon	Subnational	both	25-74	25-74	675	687	673	687	673	687			
671	Namibia	2009	Okambilimbili Survey	Community	urban	18+	18+	435	595					Accutrend		
672	Nauru	1982	Trends in the prevalence and incidence of non-insulin-dependent diabetes mellitus and impaired glucose tolerance	National	both	20+	20+	700	773							
673	Nauru	1987	Trends in the prevalence and incidence of non-insulin-dependent diabetes mellitus and impaired glucose tolerance	National	both	20+	20+	557	667							
674	Nauru	1994	Trends in the prevalence and incidence of non-insulin-dependent diabetes mellitus and impaired glucose tolerance	National	both	25+	25+	658	744							
675	Nauru	2004	STEPS	National	both	18-64	18-64	1,015	1,080							
676	Nepal	2006-2011	Early detection and management of Kidney disease, Hypertension, Diabetes and Cardiovascular disease (KHDC Nepal), Tarahara	Community	rural	18+	18+	1,175	2,347	308	671	308	671			
677	Nepal	2006-2011	Early detection and management of Kidney disease, Hypertension, Diabetes and Cardiovascular disease (KHDC Nepal), Damak	Community	urban	18+	18+	1,074	1,547	474	739	473	737			
678	Nepal	2006-2011	Early detection and management of Kidney disease, Hypertension, Diabetes and Cardiovascular disease (KHDC Nepal), Dharan	Community	urban	18+	18+	2,173	3,071	60	33	60	33			
679	Nepal	2013	STEPS	National	both	18-69	18-69	1,155	2,486	1,153	2,487	1,153	2,483			
680	Nepal	2015	Community based intervention for prevention and control of non-communicable diseases risk factors (CIPCON) baseline survey, Dhankuta	Subnational	rural	18-69	18-69	387	629					CardioChek		
681	Nepal	2015	Community based intervention for prevention and control of non-communicable diseases risk factors (CIPCON) baseline survey, Ilam	Subnational	rural	18-69	18-69	255	366					CardioChek		
682	Nepal	2016-2018	The Population Based Prevalence of Selected Non-Communicable Diseases In Nepal	National	both	20+	20+	4,274	6,780	4,286	6,807	4,263	6,765			
683	Netherlands	1985	Zutphen Elderly Study	Community	urban	65-85		886		886		886				
684	Netherlands	1990	Zutphen Elderly Study	Community	urban	69-90		555		555		555				
685	Netherlands	1989-1993	the Rotterdam Study, first subcohort	Community	urban	55+	55+	2,825	4,214	2,814	4,194	2,813	4,194			
686	Netherlands	1992-1993	The Longitudinal Aging Study Amsterdam (LASA)	Subnational	both	55-85	55-85	756	751	374	396	374	396			6
687	Netherlands	1993-1997	PROSPECT-EPIC	Subnational	both		50-69		15,849		15,849		15,849			
688	Netherlands	1995-1996	The Longitudinal Aging Study Amsterdam (LASA)	Subnational	both	65-88	65-88	635	671	193	208	193	208			6
689	Netherlands	1997-1999	the Rotterdam Study, first subcohort	Community	urban	61+	61+	1,684	2,313	1,656	2,264	1,656	2,264			
690	Netherlands	1998-2001	Regenboog Project	National	both	18-89	18-89	2,406	2,276	2,404	2,276	2,404	2,276			

	Country	Data years	Survey/Study name/Citation	Level of representative-ness	Rural, urban or both	Age range as used for global analysis		Sample size as used for global analysis (Total cholesterol)		Sample size as used for global analysis (HDL cholesterol)		Sample size as used for global analysis (Non-HDL cholesterol)		Device used for measuring total cholesterol*	Device used for measuring HDL cholesterol*	Note
						Male	Female	Male	Female	Male	Female	Male	Female			
691	Netherlands	2000-2001	the Rotterdam Study, second subcohort	Community	urban	55+	55+	1,177	1,417	1,177	1,417	1,177	1,417			
692	Netherlands	2001-2003	Surinamese in the Netherlands: Study on Ethnicity and Health (SUNSET)	Community	urban	35-60	35-60	249	254	248	254	248	254			
693	Netherlands	2002-2003	The Longitudinal Aging Study Amsterdam (LASA)	Subnational	both	54-65	54-65	341	403	121	162	121	162			6
694	Netherlands	2002-2004	the Rotterdam Study, first subcohort	Community	urban	65+	65+	1,226	1,736	1,226	1,736	1,226	1,736			
695	Netherlands	2004-2005	the Rotterdam Study, second subcohort	Community	urban	58+	58+	964	1,242	963	1,242	963	1,242			
696	Netherlands	2006-2008	the Rotterdam Study, third subcohort	Community	urban	45+	45+	1,547	2,006	1,545	2,006	1,545	2,006			
697	Netherlands	2008-2009	The Longitudinal Aging Study Amsterdam (LASA)	Subnational	both	60-100	60-100	432	491							6
698	Netherlands	2009-2010	Measuring the Netherlands (NL de Maat)	Subnational	both	30-69	30-69	1,760	1,950	1,760	1,950	1,760	1,950			
699	Netherlands	2009-2011	the Rotterdam Study, first subcohort	Community	urban	72+	72+	661	955	661	955	661	955			
700	Netherlands	2011-2012	the Rotterdam Study, second subcohort	Community	urban	65+	65+	721	903	721	903	721	903			
701	Netherlands	2011-2015	Healthy Life in an Urban Setting (HELIUS)	Community	urban	18-71	18-71	2,081	2,461	2,080	2,461	2,080	2,460			
702	Netherlands	2012-2014	the Rotterdam Study, third subcohort	Community	urban	51+	51+	1,227	1,593	1,228	1,593	1,227	1,593			
703	New Zealand	1982	MONICA, Auckland	Community	urban	35-64	35-64	1,005	562	867	522	867	521			
704	New Zealand	1989	The Life in New Zealand Survey	National	both	15+	15+	1,418	1,571							1
705	New Zealand	1993-1994	MONICA, Auckland	Community	urban	35-64	35-64	744	720	741	715	741	715			
706	New Zealand	1994	Bullen et al., N Z Med J 1998; 111: 4-7	Community	urban	65-84	65-84	476	510							
707	New Zealand	1996-1997	National Nutrition Survey	National	both	15+	15+	1,428	1,763	1,427	1,760	1,426	1,759			1
708	New Zealand	2002-2003	Diabetes, Heart and Health Survey	Subnational	urban	35-84	35-84	1,920	2,086	1,917	2,086	1,917	2,086			
709	New Zealand	2008-2009	2008/09 New Zealand Adult Nutrition Survey	National	both	15+	15+	1,444	1,865	1,443	1,865	1,443	1,864			1
710	Nicaragua	2003-2004	CAMDI	Community	urban	20+	20+	780	919							
711	Nigeria	1990	Non-communicable Diseases National Survey	National	both	15+	15+	7,857	7,207							1
712	Nigeria	1996	Okesina et al., East Afr Med J 1999; 76: 212-6	Community	rural	21-50	21-40	205	96							
713	Niue	2011	STEPS	National	both	18+	18+	291	345					Accutrend		
714	Norway	1979-1980	The Tromsø Study: Tromsø 2	Community	both	20-54	20-49	8,447	7,888	8,424	7,881	8,424	7,880			
715	Norway	1986-1987	The Tromsø Study: Tromsø 3	Community	both	20-61	20-56	10,369	9,805	10,358	9,803	10,353	9,799			
716	Norway	1992-1993	The Hordaland Health Study (HUSK) 1925-1927 birth cohort	Community	urban	65-67	65-67	2,127	2,636							
717	Norway	1992-1993	The Hordaland Health Study (HUSK) 1928-1949 birth cohort	Community	urban	43-64	43-64	335	348							
718	Norway	1992-1993	The Hordaland Health Study (HUSK) 1950-1952 birth cohort	Subnational	both	40-42	40-42	6,113	6,481							
719	Norway	1994-1995	The Tromsø Study: Tromsø 4	Community	both	25+	25+	12,780	13,843	12,753	13,826	12,747	13,819			
720	Norway	1995-1997	HUNT2 study	Subnational	rural	20+	20+	30,370	34,567	30,349	34,561	30,349	34,560			
721	Norway	1997-1999	The Hordaland Health Study (HUSK) 1925-1927 birth cohort	Community	urban	70-74	70-74	1,468	1,842	1,468	1,842	1,468	1,842			
722	Norway	1997-1999	The Hordaland Health Study (HUSK) 1950-1957 birth cohort	Subnational	both	40-47	40-47	10,165	11,937	10,167	11,936	10,163	11,934			
723	Norway	2000-2003	the Oslo cohort (HUBRO), the Oppland and Hedmark cohort (OPPHED), and the Troms and Finnmark cohort (TROFINN) of COHORT NORWAY	Subnational	both	30-76	30-76	16,792	20,241	16,786	20,241	16,784	20,240			
724	Norway	2001-2002	The Tromsø Study: Tromsø 5, Tromsø Study Panel	Community	both	30-89	30-89	2,533	3,582	2,533	3,581	2,533	3,581			
725	Norway	2006-2008	HUNT3 Study	Subnational	rural	20+	20+	22,351	26,791	22,351	26,791	22,351	26,790			
726	Norway	2007-2008	The Tromsø Study: Tromsø 6	Community	both	30-87	30-87	5,994	6,809	5,993	6,807	5,993	6,807			
727	Occupied Palestinian Territory	1996-1998	Kobar, rural	Community	rural	30-64	18-64	204	439	205	440	203	436			
728	Occupied Palestinian Territory	1996-1998	Old Ramallah, urban	Community	urban	30-64	18-64	182	456	170	449	170	448			

	Country	Data years	Survey/Study name/Citation	Level of representative-ness	Rural, urban or both	Age range as used for global analysis		Sample size as used for global analysis (Total cholesterol)		Sample size as used for global analysis (HDL cholesterol)		Sample size as used for global analysis (Non-HDL cholesterol)		Device used for measuring total cholesterol*	Device used for measuring HDL cholesterol*	Note
						Male	Female	Male	Female	Male	Female	Male	Female			
729	Occupied Palestinian Territory	2010	STEPS	National	both	18-64	18-64	2,198	3,724	2,198	3,719	2,194	3,714			
730	Oman	2008	Gulf Cooperation Council World Health Survey	National	both	18+	18+	1,880	1,808	1,805	1,715	1,805	1,715			
731	Oman	2017	STEPS	National	both	18+	18+	2,744	2,483	3,157	2,713	2,718	2,433	Multicare	Multicare	
732	Pakistan	1990-1994	National Health Survey Of Pakistan 1990-1994	National	both	18+	18+	3,221	3,937					Reflotron		
733	Pakistan	2004	COBRA-1	Community	urban	40+	40-84	1,376	1,496							
734	Palau	2011-2013	STEPS	National	both	25-64	25-64	605	651					Accutrend		
735	Panama	2010-2011	Prevalencia de factores de riesgo asociados a enfermedad cardiovascular 2010-2011	Subnational	both	18+	18+	1,073	2,476	1,073	2,477	1,072	2,476			
736	Papua New Guinea	1986	Scrimgeour et al., Pathology 1989; 21: 46-50	Community	both	17-59	17-59	43	68							1
737	Peru	2004	Factores de Riesgo de Enfermedades No Transmisibles	Community	urban	18+	18+	208	427	208	428	208	427			
738	Peru	2004-2005	CArdiovascular Risk factors Multiple Evaluation in Latin America (CARMELA)	Community	urban	25-64	25-64	769	883	769	883	769	883			
739	Peru	2004-2005	Encuesta Nacional de Indicadores Nutricionales, Bioquímicos, Socioeconómicos y Culturales Relacionados con las Enfermedades Crónicas Degenerativas (ENIN)	National	both	20+	20+	2,039	2,067	2,039	2,067	2,038	2,067			
740	Peru	2005	Factores de Riesgo de Enfermedades No Transmisibles	Community	urban	18+	18+	199	532	199	532	199	532			
741	Peru	2006	Factores de Riesgo de Enfermedades No Transmisibles	Community	urban	18+	18+	619	1,056	619	1,056	619	1,056			
742	Peru	2007-2008	PERU MIGRANT Study	Community	both	30+	30+	465	522	465	522	465	522			
743	Peru	2009-2012	CRONICAS Cohort Study	Subnational	both	35+	35+	1,518	1,597	1,518	1,597	1,518	1,597			
744	Peru	2013	Clinical functional and sociofamilial profiles of the elderly from a community in a district of Lima, Peru	Community	urban	60+	60+	101	187	101	187	101	187			
745	Peru	2013-2014	CRONICAS Cohort Study	Subnational	both	36+	36+	1,240	1,285	1,241	1,285	1,240	1,285			
746	Philippines	2003	6th National Nutrition Survey	National	both	20+	20+	2,143	2,358	2,141	2,358	2,141	2,358			
747	Philippines	2005	Cebu Longitudinal Health and Nutrition Survey 2005 Child Follow-up	Community	both	20-22	20-22	933	768	933	768	933	768			
748	Philippines	2005	Cebu Longitudinal Health and Nutrition Survey 2005 Mother Follow-up	Community	both		35-69		1,882		1,879		1,879			
749	Philippines	2013-2014	8th National Nutrition Survey	National	both	18+	18+	9,556	10,384	9,370	10,274	9,366	10,273			
750	Poland	1983-1984	MONICA, Tarnobrzeg Voivodship	Community	rural	35-64	35-64	1,225	1,431	1,225	1,431	1,225	1,431			
751	Poland	1983-1985	MONICA, Warsaw	Community	urban	35-64	35-64	1,277	1,292	1,271	1,287	1,270	1,286			
752	Poland	1987-1988	MONICA, Tarnobrzeg Voivodship	Community	rural	35-64	35-64	613	671	613	671	613	671			
753	Poland	1988-1989	MONICA, Warsaw	Community	urban	35-64	35-64	705	706	691	701	691	701			
754	Poland	1989-1990	Polish Program CINDI (CINDI Lodz 1989-1990)	Community	urban	25-64	25-64	824	950							
755	Poland	1992-1993	MONICA, Tarnobrzeg Voivodship	Community	rural	35-64	35-64	619	691	619	691	619	691			
756	Poland	1993	MONICA, Warsaw	Community	urban	35-64	35-64	748	761	742	759	742	759			
757	Poland	1995-1996	Polish Program CINDI (CINDI Lodz 1995)	Community	urban	18-64	18-64	854	1,284	835	1,260	835	1,260			
758	Poland	1997	NATPOL	National	both	18+	18+	521	547					Accutrend		
759	Poland	2000	The health status, risk factors of chronic diseases and health behaviors of residents of Torun (CINDI Torun 2000)	Community	urban	18-83	18-79	930	1,019	928	1,019	928	1,019			
760	Poland	2001-2002	The health status, risk factors of chronic diseases and health behaviors of residents of Lodz (CINDI Lodz 2001)	Community	urban	18-64	18-64	1,000	837	1,000	836	999	836			

	Country	Data years	Survey/Study name/Citation	Level of representative-ness	Rural, urban or both	Age range as used for global analysis		Sample size as used for global analysis (Total cholesterol)		Sample size as used for global analysis (HDL cholesterol)		Sample size as used for global analysis (Non-HDL cholesterol)		Device used for measuring total cholesterol*	Device used for measuring HDL cholesterol*	Note
						Male	Female	Male	Female	Male	Female	Male	Female			
761	Poland	2002	The health status, risk factors of chronic diseases and health behaviors of residents of Lodz - seniors (CINDI Lodz 2002)	Community	urban	65+	65+	288	535	287	534	287	534			
762	Poland	2002	NATPOL	National	both	18+	18+	1,023	1,303	1,022	1,303	1,022	1,303			
763	Poland	2003	The European Male Ageing Study	Community	both	40+		408		408		408				
764	Poland	2004	LIPIDOGRAM2004 Study - National epidemiological study of lipid disorders and selected risk factors of cardiovascular disease in primary health care in Poland	National	both	30+	30+	6,672	9,920	6,672	9,920	6,672	9,920			
765	Poland	2003-2005	National Multicenter Health Survey in Poland. Project WOBASZ	National	both	20-74	20-74	6,119	6,809	6,118	6,807	6,116	6,806			
766	Poland	2006	The health, risk factors for chronic diseases, attitudes and behaviors of health residents of Torun (CINDI Torun 2006)	Community	urban	18-65	18-65	750	1,115	750	1,115	750	1,115			
767	Poland	2006	LIPIDOGRAM2006 Study - National epidemiological study of lipid disorders and selected risk factors of cardiovascular disease in primary health care in Poland	National	both	32+	32+	6,440	10,640	6,439	10,638	6,439	10,638			
768	Poland	2008	The European Male Ageing Study	Community	both	40+		310		310		310				
769	Poland	2007-2011	Medical, psychological and socioeconomic aspects of aging in Poland	National	both	55+	55+	2,427	2,306	2,428	2,299	2,426	2,298			
770	Poland	2011	NATPOL	National	both	18-79	18-79	1,147	1,213	1,148	1,214	1,146	1,213			
771	Poland	2011-2014	Mogielica Human Ecology Study Site	Community	rural	45+	45+	96	337	96	337	96	337			
772	Poland	2013-2014	National Multicenter Health Survey in Poland. Project WOBASZ II	National	both	20+	20+	2,633	3,233	2,624	3,231	2,623	3,230			
773	Poland	2015-2016	LIPIDOGRAM2015 & LIPIDOGEN2015 Study - National epidemiological study of lipid disorders and selected risk factors of cardiovascular disease in primary health care in Poland	National	both	18+	18+	5,032	8,688	5,033	8,686	5,031	8,686			
774	Portugal	1999-2003	EPIPorto Study	Community	urban	18+	18+	897	1,435	868	1,411	866	1,410			
775	Portugal	2011-2013	EPITeen - Epidemiological Health Investigation of Teenagers in Porto	Community	urban	20-23	20-23	813	870	813	870	813	870			
776	Puerto Rico	2006	Perez et al., Ethn Dis 2008; 18: 434-41	Community	urban	25-84	25-84	275	532							
777	Qatar	2006	World Health Survey	National	both	18+	18+	1,567	1,707	1,528	1,692	1,527	1,684			
778	Qatar	2012	STEPS	National	both	18-64	18-64	525	879	559	868	512	838	CardioChek	CardioChek	
779	Romania	1986-1987	MONICA, Bucharest	Community	urban	25-64	25-64	636	807	194	159	194	159			
780	Romania	1997	Valorile medii si limitele normalitatii unor constante biologice; Infobase 101221a1	National	both	30-84	30-84	2,293	3,551							
781	Romania	2011-2012	Study for the Evaluation of Prevalence of Hypertension and cArdiovascular Risk among the Adult Population of Romania - SEPHAR II	National	both	18-80	18-80	931	1,037	930	1,037	930	1,037			
782	Romania	2015-2016	Study for the Evaluation of Prevalence of Hypertension and cArdiovascular Risk among the Adult Population of Romania - SEPHAR III	National	both	18-80	18-80	935	1,033	935	1,033	935	1,033			
783	Russian Federation	1984-1986	MONICA, Moscow (control)	Community	urban	35-64	35-64	738	588	715	579	706	564			
784	Russian Federation	1984-1986	MONICA, Moscow, Leninsky district	Community	urban	35-64	35-64	499	568	500	570	488	562			
785	Russian Federation	1984-1986	MONICA, Moscow, Cheremushkinsky district	Community	urban	35-64	35-64	403	433	394	433	394	433			

	Country	Data years	Survey/Study name/Citation	Level of representative-ness	Rural, urban or both	Age range as used for global analysis		Sample size as used for global analysis (Total cholesterol)		Sample size as used for global analysis (HDL cholesterol)		Sample size as used for global analysis (Non-HDL cholesterol)		Device used for measuring total cholesterol*	Device used for measuring HDL cholesterol*	Note
						Male	Female	Male	Female	Male	Female	Male	Female			
786	Russian Federation	1985	MONICA, Novosibirsk (intervention)	Community	urban	25-64	25-64	700	728	601	614	601	613			
787	Russian Federation	1985-1986	MONICA, Novosibirsk, Kirowsky district	Community	urban	25-64	25-64	673	696	652	684	652	684			
788	Russian Federation	1985-1986	MONICA, Novosibirsk, Leninsky district	Community	urban	25-64	25-64	569	571	565	564	565	564			
789	Russian Federation	1988	MONICA, Novosibirsk (intervention)	Community	urban	25-64	25-64	810	799	796	792	796	792			
790	Russian Federation	1988-1989	MONICA, Moscow (control)	Community	urban	35-64	35-64	591	540	588	552	584	536			
791	Russian Federation	1988-1989	MONICA, Moscow, Leninsky district	Community	urban	35-64	35-64	577	604	568	597	568	597			
792	Russian Federation	1988-1989	MONICA, Novosibirsk, Kirowsky district	Community	urban	25-64	25-64	823	726	804	706	803	706			
793	Russian Federation	1992	Russian Karelia Survey in Pitkaranta	Community	both	25-64	25-64	378	454	378	454	378	454			
794	Russian Federation	1992-1995	MONICA, Moscow (control)	Community	urban	35-64	35-64	546	520	545	520	545	520			
795	Russian Federation	1992-1995	MONICA, Moscow, Leninsky district	Community	urban	35-64	35-64	507	819	505	818	505	818			
796	Russian Federation	1994-1995	MONICA, Novosibirsk (intervention)	Community	urban	25-64	25-64	800	832	786	829	786	828			
797	Russian Federation	1995	MONICA, Novosibirsk, Kirowsky district	Community	urban	25-64	25-64	749	752	741	747	741	747			
798	Russian Federation	1997	Russian Karelia Survey in Pitkaranta	Community	both	25-64	25-64	309	439	309	439	309	439			
799	Russian Federation	2002	Russian Karelia Survey in Pitkaranta	Community	both	25-64	25-64	248	331	248	331	248	331			
800	Russian Federation	2007	Russian Karelia Survey in Pitkaranta	Community	both	25-64	25-64	174	271	174	272	174	271			
801	Russian Federation	2015-2017	Ural Eye and Medical Study (UEMS)	Subnational	rural	40+	40+	1,515	1,861							
802	Russian Federation	2015-2017	Ural Eye and Medical Study (UEMS)	Subnational	urban	40+	40+	1,030	1,429							
803	Rwanda	2012	STEPS	National	both	18-64	18-64	1,319	2,589	2,318	3,802	1,278	2,517	CardioChek	CardioChek	
804	Saint Vincent and the Grenadines	2013-2014	STEPS	National	both	18-69	18-69	361	555	399	578	337	526	CardioChek	CardioChek	
805	Samoa	1995	McGarvey, Pac Health Dialog 2001; 8: 157-62	National	both	29-69	29-59	131	137	129	138	129	137			
806	Samoa	2002	STEPS	National	both	25-64	25-64	634	747					Accutrend		
807	Samoa	2010	Samoan Genome-Wide Association Study	National	both	24-65	24-65	1,170	1,775	1,170	1,773	1,170	1,773			
808	Samoa	2013	STEPS	National	both	18-64	18-64	392	576					Accutrend		
809	Sao Tome and Principe	2009	STEPS	National	both	25-64	25-64	321	495					Accutrend		
810	Saudi Arabia	1992	Rahman Al-Nuaim, Int J Cardiol 1997; 62: 227-35	National	both	30-64	30-64	1,043	1,012							
811	Saudi Arabia	2004-2005	STEPS	National	both	15-64	15-64	2,200	2,286	2,199	2,286	2,199	2,286			1
812	Saudi Arabia	2011-2012	Jeeluna Study - National Assessment of the Health Needs of Adolescents in Saudi Arabia	National	both	18-19	18-19	879	573	880	573	879	573			
813	Saudi Arabia	2013	Saudi Health Information Survey	National	both	15+	15+	2,369	2,711	2,537	2,945	2,537	2,945			1
814	Serbia	1984	MONICA, Novi Sad	Community	urban	25-64	25-64	786	773	777	771	774	769			
815	Serbia	1988-1989	MONICA, Novi Sad	Community	urban	25-64	25-64	777	790	776	786	776	786			
816	Serbia	1994-1995	MONICA, Novi Sad	Community	urban	25-64	25-64	596	666	592	659	591	659			
817	Serbia	2013-2014	Stay Fit for Lifelong Health; the Prevalence of Lifestyle Health Conditions in Serbian Population	National	urban	20-69	20-59	1,337	297	1,335	297	1,335	297			
818	Seychelles	1989	Seychelles Heart Survey I	National	both	25-64	25-64	499	549	500	549	499	549			
819	Seychelles	1994	Seychelles Heart Survey Il	National	both	25-64	25-64	498	545	498	545	498	545			
820	Seychelles	2004	Seychelles Heart Survey III	National	both	25-64	25-64	559	684	556	680	556	680			
821	Seychelles	2013-2014	Seychelles Heart Survey IV	National	both	25-64	25-64	523	684	518	680	518	680			
822	Singapore	1982-1985	Thyroid Heart Study	National	both	18+	18+	1,020	984	939	943	939	943			
823	Singapore	1992	National Health Survey 1992	National	both	18-64	18-64	1,744	1,703							
824	Singapore	1993-1995	NUH Heart Study	National	both	26+	26-79	495	481	486	484	486	481			
825	Singapore	1998	National Health Survey 1998	National	both	18-64	18-64	2,278	2,263							
826	Singapore	2004	National Health Survey 2004	National	both	18-73	18-73	2,060	2,094							
827	Singapore	2004-2007	Combined follow up of Singapore Cardiovascular Cohort study and Singapore Prospective study	National	both	24+	24+	2,464	2,673	2,463	2,674	2,462	2,673			
828	Singapore	2009-2011	The Singapore Chinese Eye Study	Community	both	40-80	40-80	1,592	1,600	1,592	1,600	1,592	1,600			
829	Singapore	2012-2013	Singapore Health Study 2012	National	both	18-79	18-79	954	1,021	954	1,021	954	1,021			

	Country	Data years	Survey/Study name/Citation	Level of representative-ness	Rural, urban or both	Age range as used for global analysis		Sample size as used for global analysis (Total cholesterol)		Sample size as used for global analysis (HDL cholesterol)		Sample size as used for global analysis (Non-HDL cholesterol)		Device used for measuring total cholesterol*	Device used for measuring HDL cholesterol*	Note
						Male	Female	Male	Female	Male	Female	Male	Female			
830	Singapore	2014-2015	Singapore Health 2	National	both	20-79	20+	754	941	754	941	754	941			
831	Slovakia	1993	Countrywide Integrated Noncommunicable Diseases Intervention Programme	National	both	18-64	18-64	752	1,208	715	1,165	713	1,163			
832	Slovakia	1998	Countrywide Integrated Noncommunicable Diseases Intervention Programme	National	both	18-64	18-64	856	1,044	836	1,041	835	1,040			
833	Slovakia	2003	Countrywide Integrated Noncommunicable Diseases Intervention Programme	National	both	18-64	18-64	622	867	619	864	619	864			
834	Slovakia	2008	Countrywide Integrated Noncommunicable Diseases Intervention Programme	National	both	18-64	18-64	391	561	391	561	391	561			
835	Slovakia	2011-2012	European Health Examination Survey	National	both	18-64	18-64	879	1,076	878	1,074	878	1,074			
836	Solomon Islands	2004	A genetic-ecological study of the risk factors for lifestyle-related diseases in Oceanian populations	Community	rural	18-74	18-74	106	109	106	109	106	109			
837	Solomon Islands	2004	A genetic-ecological study of the risk factors for lifestyle-related diseases in Oceanian populations	Community	urban	18-79	20-79	91	94	91	94	91	94			
838	Solomon Islands	2006	STEPS	Subnational	both	20-64	20-64	164	266					Accutrend		
839	Solomon Islands	2015	STEPS	National	both	18-69	18-69	683	884	126	161	101	137	CardioChek	CardioChek	
840	Somalia	2016	The prevalence of selected risk factors for non-communicable diseases in Hargeisa, Somaliland: a cross-sectional study	Community	urban	20-69	20-69	59	533	58	526	58	525			
841	South Africa	1990	Mollentze et al., S Afr Med J 1995; 85: 90-6	Community	rural	25-84	25+	270	571							
842	South Africa	1990	Mollentze et al., S Afr Med J 1995; 85: 90-6	Community	urban	25-84	25-84	288	465							
843	South Africa	1995	Walker et al., QJM 1997; 90: 153-4	Community	both	60+	60+	115	146							
844	South Africa	2003	SASPI; Thorogood et al., BMC Public Health 2007; 7: 326	Community	rural	35-74	35-84	49	201							
845	South Africa	2008-2009	Cape Town Bellville South Cohort Study - Baseline evaluation I	Community	urban	18+	18+	222	717	223	717	222	717			
846	South Africa	2012	South African National Health and Nutrition Examination Survey	National	both	15+	15+	1,957	3,449	1,947	3,437	1,947	3,437			1
847	South Africa	2014-2015	Health and Aging in Africa: A Longitudinal Study of an INDEPTH Community in South Africa (HAALSI)	Community	rural	40+	40+	1,618	2,047	1,682	2,083	1,433	1,863	CardioChek	CardioChek	
848	South Korea	1998	Korea National Health and Nutrition Examination Survey	National	both	18+	18+	3,200	3,948	3,200	3,948	3,200	3,948			
849	South Korea	2001	Kim et al., Br J Psychiatry 2006; 189: 26-30	Community	both	65+	65+	300	432							
850	South Korea	2001	Korea National Health and Nutrition Examination Survey	National	both	18+	18+	2,279	2,888	2,282	2,890	2,269	2,868			
851	South Korea	2002-2003	Korean National Health Insurance	National	both	40+	40+	2,989,657	2,479,396							
852	South Korea	2005	Kweon et al., J Korean Med Sci 2005; 20: 373-8	Community	both	45-84	45-84	4,385	6,691							
853	South Korea	2005	Korea National Health and Nutrition Examination Survey	National	both	18+	18+	2,314	3,085	2,312	3,084	2,311	3,084			
854	South Korea	2004-2005	Korean National Health Insurance	National	both	40+	40+	3,601,691	3,258,695							
855	South Korea	2007	Korea National Health and Nutrition Examination Survey	National	both	18+	18+	1,152	1,615	1,152	1,615	1,152	1,615			
856	South Korea	2006-2007	Korean National Health Insurance	National	both	40+	40+	4,564,835	4,607,536							
857	South Korea	2008	Korea National Health and Nutrition Examination Survey	National	both	18+	18+	2,700	3,708	2,699	3,708	2,699	3,708			

	Country	Data years	Survey/Study name/Citation	Level of representative-ness	Rural, urban or both	Age range as used for global analysis		Sample size as used for global analysis (Total cholesterol)		Sample size as used for global analysis (HDL cholesterol)		Sample size as used for global analysis (Non-HDL cholesterol)		Device used for measuring total cholesterol*	Device used for measuring HDL cholesterol*	Note
						Male	Female	Male	Female	Male	Female	Male	Female			
858	South Korea	2009	Korea National Health and Nutrition Examination Survey	National	both	18+	18+	3,098	3,952	3,097	3,952	3,097	3,952			
859	South Korea	2008-2009	Korean National Health Insurance	National	both	40+	40+	5,764,448	6,089,851							
860	South Korea	2010	Korea National Health and Nutrition Examination Survey	National	both	18+	18+	2,542	3,297	2,542	3,297	2,542	3,297			
861	South Korea	2011	Korea National Health and Nutrition Examination Survey	National	both	18+	18+	2,516	3,330	2,516	3,330	2,516	3,330			
862	South Korea	2010-2011	Korean National Health Insurance	National	both	40+	40+	6,671,456	7,128,388	6,660,735	7,119,030	6,660,314	7,118,740			
863	South Korea	2012	Korea National Health and Nutrition Examination Survey	National	both	18+	18+	2,340	3,162	2,339	3,161	2,339	3,161			
864	South Korea	2013	Korea National Health and Nutrition Examination Survey	National	both	18+	18+	2,288	2,899	2,288	2,899	2,288	2,899			
865	South Korea	2012-2013	Korean National Health Insurance	National	both	40+	40+	7,257,240	7,784,255	7,252,867	7,781,010	7,252,410	7,780,763			
866	South Korea	2014	Korea National Health and Nutrition Examination Survey	National	both	18+	18+	2,117	2,816	2,117	2,816	2,117	2,816			
867	South Korea	2015	Korea National Health and Nutrition Examination Survey	National	both	18+	18+	2,290	2,848	2,290	2,848	2,290	2,848			
868	South Korea	2014-2015	Korean National Health Insurance	National	both	40+	40+	7,870,202	8,357,663	7,867,141	8,355,947	7,866,632	8,355,708			
869	South Korea	2016	Korea National Health and Nutrition Examination Survey	National	both	18+	18+	2,534	3,227	2,532	3,227	2,532	3,227			
870	South Korea	2017	Korea National Health and Nutrition Examination Survey	National	both	18+	18+	2,638	3,200	2,635	3,196	2,635	3,196			
871	South Korea	2016-2017	Korean National Health Insurance	National	both	40+	40+	8,535,949	9,075,677	8,532,962	9,073,811	8,532,394	9,073,560			
872	Spain	1986-1988	MONICA, Catalonia	Subnational	both	25-64	25-64	1,238	1,261	1,238	1,261	1,238	1,260			
873	Spain	1989	Cardiovascular Risk Factors Study in Catalonia	Subnational	both	15+	15+	156	158	156	158	156	158			1
874	Spain	1990	Hernandez Lanchas et al., An Med Interna 1992; 9: 64-71; Site 1	Community	urban	20-79	20-49	175	91							
875	Spain	1990	Hernandez Lanchas et al., An Med Interna 1992; 9: 64-71; Site 2	Community	urban	20-79	20-69	149	238							
876	Spain	1990-1992	MONICA, Catalonia	Subnational	both	25-64	25-64	1,661	1,152	1,660	1,152	1,660	1,152			
877	Spain	1991-1993	Encuesta de Factores de Riesgo Cardiovascular en la Región de Murcia (Cardiovascular Risk Factors Survey)	Subnational	both	18-69	18-69	1,151	1,258	1,094	1,208	1,094	1,208			
878	Spain	1995	Schroder et al., Eur J Nutr 2004; 43: 77-85	Community	both	25-74	25-74	802	868							
879	Spain	1994-1996	MONICA, Catalonia	Subnational	both	25-64	25-64	1,751	1,569	1,751	1,569	1,751	1,569			
880	Spain	1998	Tinahones et al, Metabolism 2002; 51: 429-31	Community	urban	25-64	25-64	317	538							
881	Spain	1999-2000	Factores de riesgo en las islas Baleares: Estudio CORSAIB	Subnational	both	35-74	35-74	812	867	810	863	810	863			
882	Spain	2000-2001	EUREYE Study	Subnational	both	65+	65+	254	297							
883	Spain	2001-2002	Catalan Health Interview Survey	Subnational	both	18-74	18-74	563	697	573	715	563	697			
884	Spain	2001-2003	DIabetes, Nutrición y Obesidad en la población adulta de la Región de Murcia (DINO)	Subnational	both	20+	20+	718	837	717	837	717	837			
885	Spain	2000-2005	CDC of the Canary Islands	Subnational	both	18-75	18-75	2,881	3,716	2,880	3,716	2,880	3,716			
886	Spain	2003	The European Male Ageing Study	Community	both	40-79		406		402		402				
887	Spain	2004	Vioque J et al., Obesity 2008; 16: 664-70	Community	urban	24+	24+	68	101	68	101	68	101			
888	Spain	2004	Cardiovascular Risk Study in Castilla y León (RECCyL)	Subnational	both	18+	18+	1,827	2,019	1,807	1,983	1,807	1,982			

	Country	Data years	Survey/Study name/Citation	Level of representative-ness	Rural, urban or both	Age range as used for global analysis		Sample size as used for global analysis (Total cholesterol)		Sample size as used for global analysis (HDL cholesterol)		Sample size as used for global analysis (Non-HDL cholesterol)		Device used for measuring total cholesterol*	Device used for measuring HDL cholesterol*	Note
						Male	Female	Male	Female	Male	Female	Male	Female			
889	Spain	2003-2005	Registre Gironi del Cor (REGICOR)	Subnational	both	35-79	35-79	2,951	3,280	2,952	3,280	2,951	3,280			
890	Spain	2004-2006	PREVICTUS	National	both	60+	60+	3,350	3,834	3,012	3,437	3,009	3,434			
891	Spain	2008	The European Male Ageing Study	Community	both	40+		264		261		261				
892	Spain	2007-2009	Harmonizing Equation of Risk in Mediterraneon countries EXtremadura	Subnational	both	25-79	25-79	1,297	1,498	1,296	1,496	1,296	1,496			
893	Spain	2008-2010	Study on Nutrition and Cardiovascular Risk in Spain	National	both	18+	18+	6,193	6,858	6,193	6,858	6,193	6,858			
894	Spain	2009	Cardiovascular Risk Study in Castilla y León (RECCyL)	Subnational	both	20+	20+	1,291	1,572	1,270	1,558	1,270	1,558			
895	Spain	2014	Cardiovascular Risk Study in Castilla y León (RECCyL)	Subnational	both	20+	20+	1,220	1,509	1,187	1,466	1,186	1,465			
896	Spain	2015	Study on Nutrition and Cardiovascular Risk in Spain (ENRICA)	National	both	65+	65+	704	770	703	770	703	770			
897	Sri Lanka	2014	STEPS	National	both	18-69	18-69	1,352	2,289					CardioChek		
898	Sudan (former)	2006	STEPS	National	both	25-64	25-64	145	317							
899	Sudan (former)	2016	STEPS	National	both	18-69	18-69	1,821	3,762	2,212	4,034	1,712	3,624	CardioChek	CardioChek	
900	Suriname	2013-2015	The Healthy Life in Suriname Study (HELISUR)	Subnational	urban	18-70	18-70	424	722	424	722	424	722			
901	Swaziland	2014	STEPS	National	both	18-69	18-69	731	1,507	922	1,706	709	1,462	CardioChek	CardioChek	
902	Sweden	1980	Welin et al., Diabetologia 1992; 35: 766-70; Site 1	Community	urban	67-67		529								
903	Sweden	1980	Welin et al., Diabetologia 1992; 35: 766-70; Site 2	Community	urban	67-67		66								
904	Sweden	1980-1981	Population Study of Women in Gothenburg	Community	urban		50-72		1,132							
905	Sweden	1983	Ogren et al., Lancet 1993; 342: 1138-41; Site 1	Community	urban	69-69		206								
906	Sweden	1983	Ogren et al., Lancet 1993; 342: 1138-41; Site 2	Community	urban	69-69		23								
907	Sweden	1983	Ogren et al., Lancet 1993; 342: 1138-41; Site 3	Community	urban	69-69		90								
908	Sweden	1983	Ogren et al., Lancet 1993; 342: 1138-41; Site 4	Community	urban	69-69		53								
909	Sweden	1983	Ogren et al., Lancet 1993; 342: 1138-41; Site 5	Community	urban	69-69		17								
910	Sweden	1983	Ogren et al., Lancet 1993; 342: 1138-41; Site 6	Community	urban	69-69		6								
911	Sweden	1983	Ogren et al., Lancet 1993; 342: 1138-41; Site 7	Community	urban	69-69		30								
912	Sweden	1983	Ogren et al., Lancet 1993; 342: 1138-41; Site 8	Community	urban	69-69		14								
913	Sweden	1980-1984	Uppsala Longitudinal Study of Adult Men	Community	both	60-60		593		230		219				
914	Sweden	1985	MONICA Gothenburg	Community	urban	25-64	25-64	636	689	631	682	628	682			
915	Sweden	1986	MONICA Northern Sweden	Subnational	both	25-64	25-64	823	802	822	801	822	801			
916	Sweden	1990	MONICA Northern Sweden	Subnational	both	25-64	25-64	773	799	770	803	770	798			
917	Sweden	1990	MONICA Gothenburg	Community	urban	25-64	25-64	773	774	771	772	771	772			
918	Sweden	1992	Frisk et al., Acta Odontol Scand 2003; 61: 257-62	Community	urban		35-54		148							
919	Sweden	1992-1993	Population Study of Women in Gothenburg	Community	urban		62-84		810							
920	Sweden	1993	Rosengren et al., J Intern Med 2000; 247: 111-8	Community	urban	50-50		798								
921	Sweden	1992-1994	Malmö Diet and Cancer	Community	urban	46-68	46-68	2,285	3,226	2,260	3,193	2,255	3,184			
922	Sweden	1991-1995	Uppsala Longitudinal Study of Adult Men	Community	both	70-70		1,220		1,218		1,218				
923	Sweden	1994	Helicobacter Pylori	Community	urban	56-65	56-65	170	217	170	217	170	217			
924	Sweden	1994	MONICA Northern Sweden	Subnational	both	25-74	25-74	939	975	939	974	939	974			
925	Sweden	1995	MONICA Gothenburg	Community	urban	25-64	25-64	742	863	740	861	739	861			
926	Sweden	1997	Johansson et al., J Intern Med 2002; 252: 551-60	Community	urban	35-55	35-55	137	135							
927	Sweden	1999	MONICA Northern Sweden	Subnational	both	25-74	25-74	886	927							
928	Sweden	2000	Frisk et al., Acta Odontol Scand 2003; 61: 257-62	Community	urban		65+		561							
929	Sweden	2003	The European Male Ageing Study	Community	both	40+		404		407		404				
930	Sweden	2001-2004	Swedish INTERGENE Cohort Study	Subnational	both	24-76	24-76	1,516	1,701	1,507	1,698	1,507	1,698			

	Country	Data years	Survey/Study name/Citation	Level of representative-ness	Rural, urban or both	Age range as used for global analysis		Sample size as used for global analysis (Total cholesterol)		Sample size as used for global analysis (HDL cholesterol)		Sample size as used for global analysis (Non-HDL cholesterol)		Device used for measuring total cholesterol*	Device used for measuring HDL cholesterol*	Note
						Male	Female	Male	Female	Male	Female	Male	Female			
931	Sweden	2001-2004	Prospective Investigation of the Vasculature in Uppsala Seniors (PIVUS)	Community	both	70-70	70-70	506	507	506	507	506	506			
932	Sweden	2004	LSH; Hollman et al., Eur J Cardiovasc Nurs 2008; 7: 21-6	Community	urban	45-69	45-69	497	502							
933	Sweden	2004	MONICA Northern Sweden	Subnational	both	26-75	26-75	927	975							
934	Sweden	2005	Frisk et al., Acta Odontol Scand 2003; 61: 257-62	Community	urban		75+		538							
935	Sweden	2004-2005	European Youth Heart Study (EYHS) II	Subnational	urban	20-21	20-21	67	109	68	109	67	109			
936	Sweden	2004-2005	Population Study of Women in Gothenburg	Community	urban		38-50		494		493		493			
937	Sweden	2008	The European Male Ageing Study	Community	both	40+		358		361		355				
938	Sweden	2006-2009	Prospective Investigation of the Vasculature in Uppsala Seniors (PIVUS)	Community	both	75-75	75-75	407	419	407	418	407	418			
939	Sweden	2009	MONICA Northern Sweden	Subnational	both	25-74	25-74	848	869							
940	Sweden	2007-2012	Malmö Diet and Cancer Cardiovascular Cohort Reexamination (MDC-ÅUS)	Community	urban	62-85	62-85	1,509	2,182	1,507	2,182	1,507	2,182			
941	Sweden	2011-2012	EpiHealth	National	both	45-75	45-75	4,441	5,661	4,441	5,661	4,441	5,661			
942	Sweden	2014	MONICA Northern Sweden	Subnational	both	25-74	25-74	749	800							
943	Sweden	2014-2016	Swedish INTERGENE Cohort Study	Subnational	urban	37-88	37-88	587	638	587	639	587	638			
944	Switzerland	1984-1986	The Swiss MONICA Study Wave I	Subnational	both	25-74	25-74	1,702	1,611	1,700	1,610	1,700	1,610			
945	Switzerland	1988-1989	The Swiss MONICA Study Wave II	Subnational	both	25-74	25-74	1,724	1,617	1,724	1,617	1,724	1,617			
946	Switzerland	1992-1993	The Swiss MONICA Study Wave III	Subnational	both	25-74	25-74	1,520	1,623	1,520	1,623	1,520	1,623			
947	Switzerland	2001	Bernstein et al., Arterioscler Thromb Vasc Biol 2002; 22: 133-40	Community	urban	35-74	35-74	3,552	3,531							
948	Switzerland	2003-2006	Cohorte Lausannoise (CoLaus)	Community	urban	35-75	35-75	3,177	3,532	3,177	3,531	3,177	3,531			
949	Switzerland	2007-2012	Bus Santé Study	Subnational	urban	20+	20+	1,884	1,916	1,884	1,916	1,884	1,916			
950	Switzerland	2009-2012	Cohorte Lausannoise (CoLaus)	Community	urban	40-81	40-81	2,192	2,505	2,192	2,505	2,192	2,505			
951	Switzerland	2013-2016	Bus Santé	Subnational	urban	20-74	20-74	2,011	2,145	2,012	2,145	2,011	2,144			
952	Switzerland	2014-2017	Cohorte Lausannoise (CoLaus)	Community	urban	45-87	45-87	2,022	2,488	2,022	2,487	2,022	2,487			
953	Syrian Arab Republic	2002	National Survey on non-communicable diseases and factors affecting their development	National	both	15-64	15-64	1,784	2,958							1
954	Taiwan	1993-1996	Nutrition and Health Survey in Taiwan 1993-1996	National	both	18+	18+	1,444	1,609	1,427	1,602	1,425	1,596			
955	Taiwan	1996	Lu et al., Diabet Med 1998; 15: 564-72	Subnational	urban	25-84	25-84	695	758							
956	Taiwan	1999-2000	Nutrition and Health Survey in Taiwan 1999-2000	National	both	65+	65+	1,257	1,202	1,256	1,203	1,255	1,201			
957	Taiwan	2002	Taiwanese Survey on Hypertension, Hyperglycemia and Hyperlipidemia	National	both	15+	15+	3,167	3,435	3,167	3,435	3,167	3,435			1
958	Taiwan	2005	TCHS	Community	urban	40+	40+	1,147	1,211							
959	Taiwan	2005-2008	Nutrition and Health Survey in Taiwan 2005-2008	National	both	19+	19+	1,322	1,371	1,321	1,369	1,320	1,369			
960	Taiwan	2007	Taiwanese Survey on Hypertension, Hyperglycemia and Hyperlipidemia	National	both	20+	20+	2,046	2,365	2,046	2,365	2,046	2,365			
961	Taiwan	2013-2016	Nutrition and Health Survey in Taiwan	National	both	18+	18+	1,549	1,631	1,546	1,631	1,546	1,631			
962	Tajikistan	2016	STEPS	National	both	18-69	18-69	887	1,377	1,062	1,513	883	1,372	CardioChek	CardioChek	
963	Tanzania	1990	Swai et al., Int J Epidemiol 1993; 22: 651-9; Site 1	Subnational	rural	25+	25+	1,105	1,641							
964	Tanzania	1990	Swai et al., Int J Epidemiol 1993; 22: 651-9; Site 2	Subnational	rural	25+	25+	911	889							
965	Tanzania	1990	Swai et al., Int J Epidemiol 1993; 22: 651-9; Site 3	Subnational	rural	25+	25+	241	356							
966	Tanzania	2011	STEPS	Subnational	both	25-64	25-64	748	1,200					Accutrend		
967	Tanzania	2012	STEPS	National	both	25-64	25-64	510	684					Accutrend		
968	Tanzania	2014	Dar es Salaam Urban Cohort Hypertension Study	Community	urban	40+	40+	150	239					CardioChek		
969	Thailand	1991	Thailand National Health Examination Survey I	National	both	18+	18+	5,128	6,930							

	Country	Data years	Survey/Study name/Citation	Level of representative-ness	Rural, urban or both	Age range as used for global analysis		Sample size as used for global analysis (Total cholesterol)		Sample size as used for global analysis (HDL cholesterol)		Sample size as used for global analysis (Non-HDL cholesterol)		Device used for measuring total cholesterol*	Device used for measuring HDL cholesterol*	Note
						Male	Female	Male	Female	Male	Female	Male	Female			
970	Thailand	1997	Thailand National Health Examination Survey II	National	both	18-59	18-59	933	1,572							
971	Thailand	2000	InterASIA	National	both	35+	35+	2,019	3,081	2,016	3,077	2,016	3,077			
972	Thailand	2004	Thailand National Health Examination Survey III	National	both	18+	18+	18,452	19,888							
973	Thailand	2003-2004	The Fifth National Nutrition Survey of Thailand	National	both	15-74	15-74	1,043	1,298	1,043	1,298	1,043	1,298			1
974	Thailand	2009	Thailand National Health Examination Survey IV	National	both	15+	15+	9,607	10,506	9,605	10,501	9,601	10,500			1
975	Timor-Leste	2014	STEPS	National	both	18-69	18-69	818	1,195					CardioChek		
976	Togo	2010	STEPS	National	both	18-64	18-64	458	610					Accutrend		
977	Tokelau	2005	STEPS	National	both	18-64	18-64	150	176					Accutrend		
978	Tokelau	2014	STEPS	National	both	18-64	18-64	254	277	250	267	247	267	CardioChek	CardioChek	
979	Tonga	2004	STEPS	National	both	18-64	18-64	389	543	368	531	366	526	Cholestech	Cholestech	
980	Tonga	2011	STEPS	National	both	20-64	20-64	754	1,145					Accutrend		
981	Tunisia	1989	Gharbi et al., Rev Epidemiol Sante Publique 2002; 50: 349-55; Site 1	Community	both	35-50	35-50	168	201							
982	Tunisia	1989	Gharbi et al., Rev Epidemiol Sante Publique 2002; 50: 349-55; Site 2	Community	both	35-50	35-50	146	155							
983	Tunisia	1996-1997	Ariana Healthy Project 1997	Community	both	35-65	35-65	644	758	621	742	618	739			
984	Tunisia	1996-1997	Tunisian National Nutrition Survey 1996-1997	National	both	18+	18+	1,234	2,388							
985	Tunisia	2005	Tunisian National Survey	National	both	35-71	35-71	2,644	3,782					Accutrend		
986	Tunisia	2009-2010	ObeMaghreb	Subnational	urban	18-49	18-49	980	696	978	696	977	696			
987	Tunisia	2016	Tunisian Health Examination Survey	National	both	15+	15+	3,807	4,475	4,097	4,513	3,765	4,374	CardioChek	CardioChek	1
988	Turkey	1990	Turkish Adult Risk Factor Study	National	both	20+	20+	1,342	1,371							
989	Turkey	1995	Turkish Adult Risk Factor Study	National	both	25+	25+	832	864							
990	Turkey	1998	Turkish Adult Risk Factor Study	National	both	28+	28+	858	883	851	880	851	880			
991	Turkey	1999	Tezcan et al., Trop Med Int Health 2003; 8: 660-7	Community	urban	25-64	25-64	483	727							
992	Turkey	2000	Turkish Adult Risk Factor Study	National	both	30+	30+	891	932	885	928	885	928			
993	Turkey	2000-2002	The Healthy Nutrition for Healthy Heart Study; Sanisoglu et al., BMC Public Health 2006; 6: 92	National	both	30+	30+	4,778	10,657	4,778	10,657	4,778	10,657			
994	Turkey	2001-2002	Turkish Adult Risk Factor Study	National	both	32+	32+	1,122	1,226	1,121	1,224	1,121	1,223			
995	Turkey	2003	Gokcel et al., Diabetes Care 2003; 26: 3031-4	Community	both	20-79	20-79	607	1,030							
996	Turkey	2003-2004	Turkish Adult Risk Factor Study	National	both	34+	34+	1,092	1,129	1,091	1,128	1,091	1,128			
997	Turkey	2003-2005	Prevalence of prehypertension and associated risk factors among Turkish adults: Trabzon Hypertension Study	Subnational	both	20+	20+	2,205	2,593	2,205	2,593	2,205	2,593			
998	Turkey	2005-2006	Turkish Adult Risk Factor Study	National	both	35+	35+	934	1,004	933	1,004	932	1,004			
999	Turkey	2007-2008	Turkish Adult Risk Factor Study	National	both	37+	37+	1,039	1,071	1,035	1,064	1,032	1,064			
1000	Turkey	2009-2010	Turkish Adult Risk Factor Study	National	both	39+	39+	734	794	735	799	733	793			
1001	Turkey	2011	Chronic Diseases and Risk Factors Survey in Turkey	National	both	15+	15+	6,760	7,717	6,760	7,717	6,760	7,717			1
1002	Turkey	2009-2012	Prevalence of diabetes and associated risk factors among adult population in Trabzon city	Subnational	both	20+	20+	1,525	2,078	1,529	2,085	1,518	2,074			
1003	Turkey	2012-2013	Turkish Adult Risk Factor Study	National	both	37+	40+	1,008	1,084	1,006	1,082	1,006	1,082			
1004	Turkey	2014-2015	Turkish Adult Risk Factor Study	National	both	44+	44+	845	903	840	901	840	901			
1005	Turkey	2017	STEPS	National	both	18+	18+	1,130	1,815	1,244	1,899	1,095	1,771	CardioChek	CardioChek	
1006	Turkmenistan	2013	STEPS	National	both	18-64	18-64	1,092	1,875					CardioChek		
1007	Turkmenistan	2018	STEPS	National	both	18-69	18-69	1,466	2,006	1,636	2,151	1,442	1,964	CardioChek	CardioChek	
1008	Tuvalu	2015	STEPS	National	both	18-69	18-69	389	518	379	491	337	469	CardioChek	CardioChek	

	Country	Data years	Survey/Study name/Citation	Level of representative-ness	Rural, urban or both	Age range as used for global analysis		Sample size as used for global analysis (Total cholesterol)		Sample size as used for global analysis (HDL cholesterol)		Sample size as used for global analysis (Non-HDL cholesterol)		Device used for measuring total cholesterol*	Device used for measuring HDL cholesterol*	Note
						Male	Female	Male	Female	Male	Female	Male	Female			
1009	Uganda	2011-2012	The Prevalence and Distribution of Non-communicable Diseases and Their Risk Factors in Kasese District, Uganda	Subnational	both	25-79	25-79	255	219					Reflotron		
1010	Uganda	2014	STEPS	National	both	18-69	18-69	918	1,531	1,394	1,965	874	1,477	CardioChek	CardioChek	
1011	United Arab Emirates	2017-2018	STEPS	National	both	18+	18+	1,411	1,586	1,454	1,567	1,390	1,536	CardioChek	CardioChek	
1012	United Kingdom	1983-1984	MONICA, Belfast	Subnational	both	25-64	25-64	1,142	1,170	1,120	1,142	1,116	1,139			
1013	United Kingdom	1984-1986	Scottish Heart Health Survey	Subnational	both	40-59	40-59	4,068	3,926	3,870	3,777	3,867	3,774			
1014	United Kingdom	1986-1987	Dietary and Nutritional Survey of British Adults 1986-1987	National	both	18-64	18-64	935	937	931	934	931	934			
1015	United Kingdom	1986-1987	MONICA, Belfast	Subnational	both	25-64	25-64	1,145	1,148	1,142	1,151	1,137	1,146			
1016	United Kingdom	1987-1988	Edinburgh Artery Study	Community	urban	54-75	54-75	801	772	795	771	795	771			
1017	United Kingdom	1991-1992	Health Survey for England	National	both	18+	18+	2,425	2,568							
1018	United Kingdom	1991-1992	MONICA, Belfast	Subnational	both	25-64	25-64	977	925	974	923	974	923			
1019	United Kingdom	1992	MONICA, Glasgow	Community	urban	25-64	25-64	676	726	637	694	637	694			
1020	United Kingdom	1993	Whickham Survey; Vanderpump et al., Diabet Med 1996; 13: 741-7	Community	urban	35+	35+	762	940							
1021	United Kingdom	1993	Health Survey for England	National	both	18+	18+	5,599	5,984							
1022	United Kingdom	1994	Health Survey for England	National	both	18+	18+	5,222	5,704							
1023	United Kingdom	1995	MONICA, Glasgow	Community	urban	25-64	25-64	818	877	780	853	780	853			
1024	United Kingdom	1994-1995	National Diet and Nutrition Survey (NDNS)	National	both	65+	65+	594	540	593	541	593	540			
1025	United Kingdom	1995	Scottish Health Survey (SHeS)	Subnational	both	18-64	18-64	2,768	3,246							
1026	United Kingdom	1997	National Diet and Nutrition Survey (NDNS)	National	both	18-18	18-18	32	33	32	33	32	33			
1027	United Kingdom	1998	Health Survey for England	National	both	18+	18+	4,940	5,514	4,927	5,498	4,924	5,495			
1028	United Kingdom	1998	Scottish Health Survey (SHeS)	Subnational	both	18-74	18-74	2,704	3,256	2,679	3,242	2,679	3,241			
1029	United Kingdom	1998-2000	The British Regional Heart Study	National	urban	60-79		3,938		3,912		3,912				
1030	United Kingdom	1999	MRC National Survey of Health and Development	National	both	53-54	53-54	1,277	1,281	1,141	1,235	1,141	1,235			
1031	United Kingdom	1999-2001	British Women's Heart and Health Study	National	both		60-79		3,697		3,691		3,691			4
1032	United Kingdom	2000	Health Survey for England	National	both	65+	65+	224	489	213	466	213	466			
1033	United Kingdom	1999-2004	Hertfordshire Cohort Study	Subnational	both	59-73	60-73	1,459	1,329	1,459	1,329	1,459	1,329			
1034	United Kingdom	2000-2001	National Diet and Nutrition Survey 2000-2001	National	both	19-64	19-64	580	693	579	693	579	693			
1035	United Kingdom	2003	The European Male Ageing Study	Community	both	40+		396		396		396				
1036	United Kingdom	2003	Health Survey for England	National	both	18+	18+	3,735	4,386	3,737	4,387	3,735	4,386			
1037	United Kingdom	2003	Scottish Health Survey (SHeS)	Subnational	both	18+	18+	1,846	2,175	1,847	2,175	1,846	2,175			
1038	United Kingdom	2004-2005	English Longitudinal Study of Ageing Wave 2 2004-2005	National	both	52+	52+	2,701	3,203	2,696	3,203	2,696	3,203			
1039	United Kingdom	2005	Health Survey for England	National	both	65+	65+	1,008	1,190	1,008	1,190	1,008	1,190			
1040	United Kingdom	2006	Health Survey for England	National	both	18+	18+	3,341	3,992	3,342	3,992	3,341	3,992			
1041	United Kingdom	2008	The European Male Ageing Study	Community	both	40+		311		307		307				
1042	United Kingdom	2008	Health Survey for England	National	both	18+	18+	3,274	3,857	3,274	3,856	3,274	3,856			
1043	United Kingdom	2008	Scottish Health Survey (SHeS)	Subnational	both	18+	18+	396	459	396	459	396	459			
1044	United Kingdom	2008-2009	English Longitudinal Study of Ageing Wave 4 2008-2009	National	both	50+	50+	2,871	3,500	2,869	3,497	2,869	3,497			
1045	United Kingdom	2009	Health Survey for England	National	both	18+	18+	1,058	1,214	1,058	1,214	1,058	1,214			
1046	United Kingdom	2006-2010	MRC National Survey of Health and Development	National	both	60-65	60-65	1,004	1,060	1,004	1,060	1,004	1,060			
1047	United Kingdom	2009	Scottish Health Survey (SHeS)	Subnational	both	18+	18+	359	467	359	467	359	467			
1048	United Kingdom	2010	Health Survey for England	National	both	18+	18+	1,680	2,129	1,680	2,129	1,680	2,129			
1049	United Kingdom	2008-2012	National Diet and Nutrition Survey (NDNS)	National	both	18+	18+	460	593	460	593	460	593			

	Country	Data years	Survey/Study name/Citation	Level of representative-ness	Rural, urban or both	Age range as used for global analysis		Sample size as used for global analysis (Total cholesterol)		Sample size as used for global analysis (HDL cholesterol)		Sample size as used for global analysis (Non-HDL cholesterol)		Device used for measuring total cholesterol*	Device used for measuring HDL cholesterol*	Note
						Male	Female	Male	Female	Male	Female	Male	Female			
1050	United Kingdom	2010	Scottish Health Survey (SHeS)	Subnational	both	18+	18+	336	432	336	432	336	432			
1051	United Kingdom	2011	Health Survey for England	National	both	18+	18+	1,712	2,172	1,710	2,172	1,709	2,172			
1052	United Kingdom	2011	Scottish Health Survey (SHeS)	Subnational	both	18+	18+	319	366	319	366	319	366			
1053	United Kingdom	2012	Health Survey for England	National	both	18+	18+	1,718	2,169	1,715	2,169	1,715	2,169			
1054	United Kingdom	2012-2013	English Longitudinal Study of Ageing Wave 6 2012-2013	National	both	50+	50+	2,731	3,308	2,729	3,307	2,729	3,307			
1055	United Kingdom	2013	Health Survey for England	National	both	18+	18+	2,039	2,391	2,037	2,390	2,037	2,390			
1056	United Kingdom	2014	Health Survey for England	National	both	18+	18+	1,785	2,067	1,786	2,067	1,785	2,067			
1057	United Kingdom	2013-2014	National Diet and Nutrition Survey (NDNS)	National	both	18+	18+	293	443	293	443	293	443			
1058	United Kingdom	2015	Health Survey for England	National	both	18+	18+	1,749	2,109	1,749	2,110	1,749	2,109			
1059	United Kingdom	2015	MRC National Survey of Health and Development	National	both	69-70	69-70	952	1,010	949	1,009	948	1,008			
1060	United Kingdom	2016	Health Survey for England	National	both	18+	18+	1,659	2,064	1,659	2,066	1,659	2,064			
1061	United Kingdom	2015-2016	National Diet and Nutrition Survey (NDNS)	National	both	18+	18+	300	396	300	396	300	396			
1062	United Kingdom	2017	Health Survey for England	National	both	18+	18+	1,694	2,141	1,694	2,141	1,694	2,141			
1063	United Kingdom	2016-2017	National Diet and Nutrition Survey (NDNS)	National	both	18+	18+	130	171	129	171	129	171			
1064	United States of America	1976-1980	US NHANES II	National	both	20-74	20-74	5,601	6,245	4,558	5,225	4,558	5,225			2
1065	United States of America	1979-1980	MONICA, Stanford	Subnational	urban	25-64	25-64	692	791	689	789	689	789			7
1066	United States of America	1981-1982	The Bogalusa Heart Study	Community	rural	18-22	18-22	38	24	37	24	37	24			
1067	United States of America	1983-1985	The Bogalusa Heart Study	Community	rural	18-22	18-22	50	24	49	24	49	24			
1068	United States of America	1985-1986	Coronary Artery Risk Development in Young Adults (CARDIA)	Subnational	urban	18-30	18-30	2,307	2,757	2,306	2,757	2,306	2,757			
1069	United States of America	1985-1986	MONICA, Stanford	Subnational	urban	25-64	25-64	703	827	703	825	703	825			7
1070	United States of America	1987-1989	Atherosclerosis Risk in Communities Study	Subnational	both	44-66	44-66	4,974	6,070	4,969	6,071	4,968	6,067			
1071	United States of America	1987-1988	The Bogalusa Heart Study	Community	rural	18-22	18-22	40	27	40	27	40	27			
1072	United States of America	1989-1990	Cardiovascular Health Study	Subnational	both	65+	65+	2,451	3,287	2,447	3,282	2,446	3,282			
1073	United States of America	1989-1990	MONICA, Stanford	Subnational	urban	25-64	25-64	694	803	694	802	694	801			7
1074	United States of America	1990-1992	Atherosclerosis Risk in Communities Study	Subnational	both	46-70	46-70	4,528	5,579	4,509	5,549	4,508	5,549			
1075	United States of America	1990-1991	Coronary Artery Risk Development in Young Adults (CARDIA)	Subnational	urban	23-35	23-35	1,929	2,314	1,929	2,314	1,929	2,314			
1076	United States of America	1988-1994	US NHANES III	National	both	18+	18+	7,846	8,592	7,773	8,532	7,771	8,528			
1077	United States of America	1992-1994	The Bogalusa Heart Study	Community	rural	18-21	18-21	53	33	53	33	53	33			
1078	United States of America	1992-1993	Coronary Artery Risk Development in Young Adults (CARDIA)	Subnational	urban	25-37	25-37	1,814	2,192	1,813	2,192	1,812	2,192			
1079	United States of America	1992-1993	Cardiovascular Health Study	Subnational	both	65+	65+	1,970	2,714	1,964	2,710	1,964	2,710			
1080	United States of America	1993-1995	Atherosclerosis Risk in Communities Study	Subnational	both	50-73	48-73	3,991	4,991	3,987	4,988	3,987	4,988			
1081	United States of America	1993-1994	Cardiovascular Health Study	Subnational	both	65+	65+	1,764	2,464							
1082	United States of America	1994-1995	Cardiovascular Health Study	Subnational	both	65+	65+	1,617	2,352							
1083	United States of America	1995-1996	The Bogalusa Heart Study	Community	rural	20-39	20-39	548	829	548	829	548	829			
1084	United States of America	1995-1996	Coronary Artery Risk Development in Young Adults (CARDIA)	Subnational	urban	28-40	28-40	1,729	2,140	1,730	2,140	1,729	2,140			
1085	United States of America	1996-1998	Atherosclerosis Risk in Communities Study	Subnational	both	50-75	50-75	3,528	4,447	3,527	4,446	3,527	4,446			
1086	United States of America	1996-1997	Cardiovascular Health Study	Subnational	both	65+	65+	1,330	1,996							
1087	United States of America	1996-1997	Study of Women's Health Across the Nation	Subnational	both		40-55		3,218		3,218		3,218			8
1088	United States of America	1997-1998	Cardiovascular Health Study	Subnational	both	65+	65+	1,185	1,804							
1089	United States of America	1997-1999	Study of Women's Health Across the Nation	Subnational	both		40-55		2,738		2,735		2,735			8
1090	United States of America	1999-2000	US NHANES 1999-2000	National	both	18+	18+	2,172	2,187	2,170	2,186	2,170	2,185			
1091	United States of America	1999-2001	Study of Women's Health Across the Nation	Subnational	both		40-56		2,331		2,328		2,328			8

	Country	Data years	Survey/Study name/Citation	Level of representative-ness	Rural, urban or both	Age range as used for global analysis		Sample size as used for global analysis (Total cholesterol)		Sample size as used for global analysis (HDL cholesterol)		Sample size as used for global analysis (Non-HDL cholesterol)		Device used for measuring total cholesterol*	Device used for measuring HDL cholesterol*	Note
						Male	Female	Male	Female	Male	Female	Male	Female			
1092	United States of America	2000-2001	Coronary Artery Risk Development in Young Adults (CARDIA)	Subnational	urban	33-45	33-45	1,560	1,941	1,559	1,941	1,558	1,941			
1093	United States of America	2000-2002	Study of Women's Health Across the Nation	Subnational	both		40-57		2,280		2,280		2,280			8
1094	United States of America	2001-2002	US NHANES 2001-2002	National	both	18+	18+	2,511	2,389	2,511	2,389	2,511	2,389			
1095	United States of America	2003-2004	US NHANES 2003-2004	National	both	18+	18+	2,419	2,341	2,419	2,340	2,419	2,340			
1096	United States of America	2005-2006	Coronary Artery Risk Development in Young Adults (CARDIA)	Subnational	urban	38-50	38-50	1,522	1,982	1,521	1,982	1,520	1,982			
1097	United States of America	2005-2006	Cardiovascular Health Study	Subnational	both	65+	65+	375	660	375	660	375	660			
1098	United States of America	2005-2006	US NHANES 2005-2006	National	both	18+	18+	2,413	2,247	2,413	2,246	2,413	2,246			
1099	United States of America	2007-2008	US NHANES 2007-2008	National	both	18+	18+	2,760	2,777	2,597	2,601	2,597	2,601			
1100	United States of America	2009-2010	US NHANES 2009-2010	National	both	18+	18+	2,909	2,999	2,908	2,999	2,908	2,999			
1101	United States of America	2010-2011	Coronary Artery Risk Development in Young Adults (CARDIA)	Subnational	urban	43-55	43-55	1,509	1,968	1,509	1,967	1,508	1,967			
1102	United States of America	2011-2013	Atherosclerosis Risk in Communities Study	Subnational	both	67-90	67-90	1,823	2,462	1,823	2,462	1,823	2,462			
1103	United States of America	2011-2012	US NHANES 2011-2012	National	both	18+	18+	2,575	2,561	2,574	2,562	2,574	2,561			
1104	United States of America	2013-2014	US NHANES 2013-2014	National	both	18+	18+	2,684	2,894	2,682	2,895	2,682	2,894			
1105	United States of America	2015-2016	US NHANES 2015-2016	National	both	18+	18+	2,606	2,728	2,604	2,727	2,604	2,727			
1106	Uruguay	2006	STEPS	National	both	25-64	25-64	185	471	186	478	184	471			
1107	Uruguay	2011-2012	CESCAS Study	Community	urban	30-79	30-79	634	909	635	909	634	909			
1108	Uruguay	2012-2016	Genotype, Phenotype and Environment of Hypertension in Uruguay (GEFA-HT-UY)	Community	urban	19+	20+	129	192	129	192	129	192			
1109	Uzbekistan	2002	Demographic and Health Survey Uzbekistan 2002	Subnational	urban	18-59	18-49	550	626	547	622	547	622			
1110	Uzbekistan	2014	STEPS	National	both	18-64	18-64	1,249	1,961					Multicare		
1111	Vanuatu	2011	STEPS	National	both	25-64	25-64	2,062	1,952					Accutrend		
1112	Venezuela	2000	Zulia Coronary Heart Disease Risk Factor Study; Florez et al., Diabetes Res Clin Pract 2005; 69: 63-77	Subnational	both	25+	25+	832	1,939							
1113	Venezuela	2004-2005	CArdiovascular Risk factors Multiple Evaluation in Latin America (CARMELA)	Community	urban	25-64	25-64	713	1,135	713	1,135	713	1,135			
1114	Venezuela	2005-2006	Brajkovich et al., Rev Ven Endoc Metab 2006; 4: 31-32	Community	urban	20-65	20-65	204	438	204	439	204	438			
1115	Venezuela	2007-2008	Venezuelan Study of Metabolic Syndrome, Obesity and Lifestyle (VEMSOLS)	Community	urban	20-79	20+	107	230	107	230	107	230			
1116	Venezuela	2008-2009	Venezuelan Study of Metabolic Syndrome, Obesity and Lifestyle (VEMSOLS)	Community	rural	20+	20-79	51	89	48	89	48	89			
1117	Venezuela	2010-2011	Venezuelan Study of Metabolic Syndrome, Obesity and Lifestyle (VEMSOLS)	Community	urban	20+	20+	51	154	49	149	49	149			
1118	Venezuela	2015-2017	Cardio-Metabolic Health Venezuelan Study (EVESCAM)	National	both	20+	20+	1,053	2,338	1,045	2,328	1,043	2,325			
1119	Viet Nam	2005	Non-communicable disease risk factors in Ho Chi Minh City	Community	urban	25-64	25-64	504	717					Accu-Chek		
1120	Viet Nam	2008-2009	The survey on diabetes and its risk factors in 2 northern provinces of Vietnam (DM-S)	Subnational	both	25+	25+	785	1,345	785	1,345	785	1,345			
1121	Viet Nam	2009	STEPS	National	both	25-64	25-64	5,369	6,581					Accutrend		
1122	Viet Nam	2015	STEPS	National	both	18-69	18-69	1,217	1,650	1,295	1,703	1,200	1,636	CardioChek	CardioChek	
1123	Yemen	2007-2009	Hypertension and Diabetes in Yemen (HYDY)	National	rural	18-70	18-70	494	704					Multicare		
1124	Yemen	2007-2009	Hypertension and Diabetes in Yemen (HYDY)	National	urban	18-70	18-70	875	1,039					Multicare		
1125	Zambia	2008	STEPS	Subnational	urban	25+	25+	347	731					Accutrend		

	Country	Data years	Survey/Study name/Citation	Level of representative-ness	Rural, urban or both	Age range as used for global analysis		Sample size as used for global analysis (Total cholesterol)		Sample size as used for global analysis (HDL cholesterol)		Sample size as used for global analysis (Non-HDL cholesterol)		Device used for measuring total cholesterol*	Device used for measuring HDL cholesterol*	Note
						Male	Female	Male	Female	Male	Female	Male	Female			
1126	Zambia	2017	STEPS	National	both	18-69	18-69	768	1,545					CardioChek		
1127	Zimbabwe	2005	STEPS	National	both	25+	25+	504	1,628	504	1,628	504	1,628			

* In surveys that used a portable device for measuring lipids.

1. The first age group started from <18 years old, but had a mean age ≥18 years.

2. National study conducted between 1977 and 1979, included in the analysis as a 1980 study.

3. This research uses data from China Health and Nutrition Survey (CHNS). We thank the National Institute of Nutrition and Food Safety, China Center for Disease Control and Prevention, Carolina Population Center (5 R24 HD050924), the University of North Carolina at Chapel Hill, the NIH (R01-HD30880, DK056350, R24-HD050924, and R01-HD38700) and the Fogarty International Center, NIH for financial support for the CHNS data collection and analysis files from 1989 to 2011 and future surveys, and the China-Japan Friendship Hospital, Ministry of Health for support for CHNS 2009.

4. The British Women’s Heart and Health Study is supported by the British Heart Foundation (PG/13/66/30442). British Women’s Heart and Health Study data are available to bona fide researchers for research purposes. Please refer to the BWHHS data sharing policy at http://www.ucl.ac.uk/british-womens-heart-health-study.

5. The Older Persons in Jamaica Study was funded by the National Health Fund, Jamaica.

6. The Longitudinal Aging Study Amsterdam is supported by a grant from the Netherlands Ministry of Health Welfare and Sports, Directorate of Long-Term Care.

7. We thank Prof Stephen Fortmann for data from the Stanford Five-City Project.

8. The bibliographic citation for this data source is: Sutton-Tyrrell, Kim, Faith Selzer, MaryFran Sowers, Robert Neer, Lynda Powell, Ellen Gold, Gail Greendale, Gerson Weiss, Karen Matthews, and Sonja McKinlay. Study of Women's Health Across the Nation (SWAN), 1996-1997: Baseline Dataset. ICPSR28762-v2. Ann Arbor, MI: Inter-university Consortium for Political and Social Research[distributor], 2014-02-04. http://doi.org/10.3886/ICPSR28762.v2

9. Electrophoresis was used for measuring lipid fractions. As this method may be inaccurate in quantifying lipid fractions, mean HDL and non-HDL cholesterol were not included in this analysis.

Supplementary Table 2. List of analysis regions and “super-regions”, and countries in each region. The hierarchical structure of the statistical model consisted of country, region, super-region, and world.

Super-region	Region
**Sub-Saharan Africa (48)**	**Central Africa (6)**: Angola, Central African Republic, Congo, DR Congo, Equatorial Guinea, Gabon
	**East Africa (17)**: Burundi, Comoros, Djibouti, Eritrea, Ethiopia, Kenya, Madagascar, Malawi, Mauritius†, Mozambique, Rwanda, Seychelles†, Somalia, Sudan (former), Tanzania, Uganda, Zambia
	**Southern Africa (6)**: Botswana, Lesotho, Namibia, South Africa, Swaziland, Zimbabwe
	**West Africa (19)**: Benin, Burkina Faso, Cabo Verde, Cameroon, Chad, Cote d'Ivoire, Gambia, Ghana, Guinea, Guinea Bissau, Liberia, Mali, Mauritania, Niger, Nigeria, Sao Tome and Principe, Senegal, Sierra Leone, Togo
**Central Asia, Middle East and North Africa (28)**	**Central Asia (9)**: Armenia, Azerbaijan, Georgia, Kazakhstan, Kyrgyzstan, Mongolia, Tajikistan, Turkmenistan, Uzbekistan
	**Middle East and North Africa (19)**: Algeria, Bahrain, Egypt, Iran, Iraq, Jordan, Kuwait, Lebanon, Libya, Morocco, Occupied Palestinian Territory, Oman, Qatar, Saudi Arabia, Syrian Arab Republic, Tunisia, Turkey, United Arab Emirates, Yemen
**South Asia (6)**	**South Asia (6)**: Afghanistan, Bangladesh, Bhutan, India, Nepal, Pakistan
**East and Southeast Asia (16)**	**East Asia (4)**: China, China (Hong Kong SAR), North Korea, Taiwan
	**Southeast Asia (12)**: Brunei Darussalam, Cambodia, Indonesia, Lao PDR, Malaysia, Maldives, Myanmar, Philippines, Sri Lanka, Thailand, Timor-Leste, Viet Nam
**Oceania (17)**	**Polynesia and Micronesia (13)**: American Samoa, Cook Islands, French Polynesia, Kiribati, Marshall Islands, Micronesia (Federated States of), Nauru, Niue, Palau, Samoa, Tokelau, Tonga, Tuvalu
	**Melanesia (4):** Fiji, Papua New Guinea, Solomon Islands, Vanuatu
**High-income Asia Pacific (3)**	**High-income Asia Pacific (3)**: Japan, Singapore, South Korea
**Latin America and Caribbean (35)**	**Andean Latin America (3)**: Bolivia, Ecuador, Peru
	**Caribbean (18)**: Antigua and Barbuda, Bahamas, Barbados, Belize, Bermuda, Cuba, Dominica, Dominican Republic, Grenada, Guyana, Haiti, Jamaica, Puerto Rico, Saint Kitts and Nevis, Saint Lucia, Saint Vincent and the Grenadines, Suriname, Trinidad and Tobago
	**Central Latin America (9)**: Colombia, Costa Rica, El Salvador, Guatemala, Honduras, Mexico, Nicaragua, Panama, Venezuela
	**Southern Latin America (5)**: Argentina, Brazil, Chile, Paraguay, Uruguay
**High-income Western countries (27)**	**High-income English-speaking countries* (6)**: Australia, Canada, Ireland, New Zealand, United Kingdom, United States of America
	**North Western Europe (12)**: Austria, Belgium, Denmark, Finland, Germany, Greenland, Iceland, Luxembourg, Netherlands, Norway, Sweden, Switzerland
	**South Western Europe (9)**: Andorra, Cyprus, France, Greece, Israel, Italy, Malta, Portugal, Spain
**Central and Eastern Europe (20)**	**Central Europe (13)**: Albania, Bosnia and Herzegovina, Bulgaria, Croatia, Czech Republic, Hungary, Macedonia (TFYR), Montenegro, Poland, Romania, Serbia, Slovakia, Slovenia
	**Eastern Europe (7)**: Belarus, Estonia, Latvia, Lithuania, Moldova, Russian Federation, Ukraine

† Mauritius and Seychelles were grouped with Polynesia and Micronesia in the hierarchical analysis, because of their epidemiological similarity.

* Although high-income English-speaking countries are geographically separated, they exhibit remarkably similar trends in cardiometabolic risk factors and outcomes.[2-5] They were therefore grouped together so that the statistical model shares information amongst them more than it does with other countries that are geographically closer but epidemiologically more distinct.

We did not have data on population by age group for American Samoa, Bermuda, French Polynesia, Greenland, and Tokelau. Country-specific estimates were made but were not used in calculation of regional and global means because the latter requires weighting by age-specific population.

Supplementary Table 3. Analytical range of portable devices used for measuring lipids. Numbers in brackets show the restricted range of cholesterol levels used for calculating cholesterol means for each portable device.

Portable device	Analytical range (mmol/L)	
	Total cholesterol	HDL cholesterol
Accutrend	3.88-7.76 (4.0-7.5)	
Accu-Chek	3.88-7.76 (4.0-7.5)	
CardioChek	2.59-10.36 (2.8-10.0)	0.39-2.59 (0.4-2.4)
Cholestech	2.58-12.92 (2.8-12.6)	0.39-2.59 (0.4-2.4)
LipidoCare	2.59-11.65 (2.8-11.3)	0.65-2.47 (0.8-2.3)
LipidPro	2.59-10.36 (2.8-10.0)	
Multicare	3.30-10.20 (3.5-10.0)	0.39-2.59 (0.4-2.4)
Reflotron	2.59-12.90 (2.8-12.6)	0.26-2.59 (0.4-2.4)

HDL: high-density lipoprotein.

Supplementary Table 4. Model specifications and regression coefficients to adjust for the differences in mean total, non-HDL and HDL cholesterol between portable device and laboratory measurement.

The dependent variable in all regressions was means, fitted using a linear model.

* denotes statistical interaction. CI: confidence interval; TC: total cholesterol; HDL: high-density lipoprotein.

Conversion regression for Accutrend and Accu-Chek (TC)	
**Variables**	**Coefficients (95% CI)**
Intercept	-3.5 (-3.7, -3.31)
Unadjusted mean TC (calculated over restricted cholesterol range: 4.0-7.5 mmol/L)	1.65 (1.61, 1.68)
Mean age of age group	0.00781 (0.00446, 0.0112)
Male sex	-0.199 (-0.315, -0.0823)
Unadjusted mean TC * mean age of age group	-0.00162 (-0.00227, -0.000978)
Unadjusted mean TC * male sex	0.0286 (0.00677, 0.0505)
Number of data points used to fit the model = 4,363	
R^2 = 0.952	

Conversion regression for CardioChek and LipidPro (TC)	
**Variables**	**Coefficients (95% CI)**
Intercept	-0.251 (-0.283, -0.218)
Unadjusted mean TC (calculated over restricted cholesterol range: 2.8-10.0 mmol/L)	1.05 (1.04, 1.06)
Mean age of age group	0.00112 (0.000573, 0.00167)
Male sex	-0.0322 (-0.0527, -0.0116)
Unadjusted mean TC * mean age of age group	-0.00027 (-0.00038, -0.000159)
Unadjusted mean TC * male sex	0.00481 (0.000847, 0.00876)
Number of data points used to fit the model = 4,552	
R^2 = 0.996	

Conversion regression for Cholestech and Reflotron (TC)	
**Variables**	**Coefficients (95% CI)**
Intercept	-0.229 (-0.257, -0.201)
Unadjusted mean TC (calculated over restricted cholesterol range: 2.8-12.6 mmol/L)	1.04 (1.04, 1.05)
Mean age of age group	0.00191 (0.00143, 0.00239)
Male sex	-0.0604 (-0.0782, -0.0425)
Unadjusted mean TC * mean age of age group	-0.000412 (-0.000508, -0.000316)
Unadjusted mean TC * male sex	0.0103 (0.00686, 0.0137)
Number of data points used to fit the model = 4,553	
R^2 = 0.997	

Conversion regression for LipidoCare (TC)	
**Variables**	**Coefficients (95% CI)**
Intercept	-0.241 (-0.27, -0.211)
Unadjusted mean TC (calculated over restricted cholesterol range: 2.8-11.3 mmol/L)	1.05 (1.04, 1.05)
Mean age of age group	0.00179 (0.00128, 0.00229)
Male sex	-0.0528 (-0.0716, -0.034)
Unadjusted mean TC * mean age of age group	-0.000392 (-0.000493, -0.00029)
Unadjusted mean TC * male sex	0.00883 (0.00521, 0.0125)
Number of data points used to fit the model = 4,552	
R^2 = 0.997	

Conversion regression for Multicare (TC)	
**Variables**	**Coefficients (95% CI)**
Intercept	-1.29 (-1.37, -1.2)
Unadjusted mean TC (calculated over restricted cholesterol range: 3.5-10.0 mmol/L)	1.24 (1.22, 1.25)
Mean age of age group	0.011 (0.00953, 0.0124)
Male sex	-0.228 (-0.279, -0.177)
Unadjusted mean TC * mean age of age group	-0.00219 (-0.00247, -0.0019)
Unadjusted mean TC * male sex	0.0381 (0.0284, 0.0478)
Number of data points used to fit the model = 4,503	
R^2 = 0.981	

Conversion regression for CardioChek (non-HDL cholesterol)*	
**Variables**	**Coefficients (95% CI)**
Intercept	-0.129 (0.159, -0.0982)
Unadjusted mean non-HDL cholesterol (calculated as TC minus HDL cholesterol, using only TC and HDL cholesterol values within restricted cholesterol range: 2.8-10.0 mmol/L for TC and 0.4-2.4 mmol/L for HDL cholesterol)	1.07 (1.03, 1.11)
Mean age of age group	0.000217 (-0.000285, 0.000718)
Male sex	-0.0262 (-0.0449, -0.00758)
Unadjusted mean TC (calculated over restricted cholesterol range: 2.8-10.0 mmol/L)	-0.0385 (-0.0769, -0.00013)
Unadjusted mean HDL cholesterol (calculated over restricted cholesterol range: 0.4-2.4 mmol/L)	0.0514 (0.0105, 0.0924)
Unadjusted mean non-HDL cholesterol * mean age of age group	-0.000119 (-0.000256, 0.0000181)
Unadjusted mean non-HDL cholesterol * male sex	0.0051 (0.000312, 0.00988)
Number of data points used to fit the model = 3,956	
R^2 = 0.995	

* Both total and HDL cholesterol were measured using the portable device CardioChek.

**Conversion regression for Cholestech and Reflotron (non-HDL cholesterol)***	
**Variables**	**Coefficients (95% CI)**
Intercept	-0.122 (-0.149, -0.0958)
Unadjusted mean non-HDL cholesterol (calculated as TC minus HDL cholesterol, using only TC and HDL cholesterol values within restricted cholesterol range: 2.8-12.6 mmol/L for TC and 0.4-2.4 mmol/L for HDL cholesterol)	1.07 (1.04, 1.1)
Mean age of age group	0.0009 (0.000464, 0.00134)
Male sex	-0.0477 (-0.064, -0.0315)
Unadjusted mean TC (calculated over restricted cholesterol range: 2.8-12.6 mmol/L)	-0.0468 (-0.0793, -0.0142)
Unadjusted mean HDL cholesterol (calculated over restricted cholesterol range: 0.4-2.4 mmol/L)	0.0629 (0.0281, 0.0976)
Unadjusted mean non-HDL cholesterol * mean age of age group	-0.000285 (-0.000404, -0.000166)
Unadjusted mean non-HDL cholesterol * male sex	0.0112 (0.00708, 0.0154)
Number of data points used to fit the model = 3,957	
R^2 = 0.996	

* Both total and HDL cholesterol were measured using the portable device Cholestech or Reflotron.

**Conversion regression for LipidoCare (non-HDL cholesterol)***	
**Variables**	**Coefficients (95% CI)**
Intercept	0.027 (-0.0096, 0.0636)
Unadjusted mean non-HDL cholesterol (calculated as TC minus HDL cholesterol, using only TC and HDL cholesterol values within restricted cholesterol range: 2.8-11.3 mmol/L for TC and 0.8-2.3 mmol/L for HDL cholesterol)	0.522 (0.493, 0.55)
Mean age of age group	0.00102 (0.000442, 0.0016)
Male sex	-0.0213 (-0.0428, 0.0002)
Unadjusted mean TC (calculated over restricted cholesterol range: 2.8-11.3 mmol/L)	0.515 (0.487, 0.542)
Unadjusted mean HDL cholesterol (calculated over restricted cholesterol range: 0.8-2.3 mmol/L)	-0.631 (-0.665, -0.596)
Unadjusted mean non-HDL cholesterol * mean age of age group	-0.000379 (-0.000537, -0.000222)
Unadjusted mean non-HDL cholesterol * male sex	0.00346 (-0.00204, 0.00896)
Number of data points used to fit the model = 3,921	
R^2 = 0.993	

* Both total and HDL cholesterol were measured using the portable device LipidoCare.

**Conversion regression for Multicare (non-HDL cholesterol)***	
**Variables**	**Coefficients (95% CI)**
Intercept	-0.867 (-0.931, -0.802)
Unadjusted mean non-HDL cholesterol (calculated as TC minus HDL cholesterol, using only TC and HDL cholesterol values within restricted cholesterol range: 3.5-10.0 mmol/L for TC and 0.4-2.4 mmol/L for HDL cholesterol)	1.49 (1.42, 1.56)
Mean age of age group	0.00449 (0.00342, 0.00555)
Male sex	-0.195 (-0.234, -0.157)
Unadjusted mean TC (calculated over restricted cholesterol range: 3.5-10.0 mmol/L)	-0.345 (-0.414, -0.275)
Unadjusted mean HDL cholesterol (calculated over restricted cholesterol range: 0.4-2.4 mmol/L)	0.539 (0.466, 0.612)
Unadjusted mean non-HDL cholesterol * mean age of age group	-0.00122 (-0.0015, -0.000933)
Unadjusted mean non-HDL cholesterol * male sex	0.0464 (0.0366, 0.0561)
Number of data points used to fit the model = 3,912	
R^2 = 0.982	

* Both total and HDL cholesterol were measured using the portable device Multicare.

**Conversion regression for Accutrend (non-HDL cholesterol)***	
**Variables**	**Coefficients (95% CI)**
Intercept	-2.52 (-2.66, -2.38)
Unadjusted mean non-HDL cholesterol (calculated as TC minus HDL cholesterol, using only TC values within restricted cholesterol range: 4.0-7.5 mmol/L)	1.79 (1.67, 1.92)
Mean age of age group	-0.00214 (-0.00446, 0.000178)
Male sex	-0.236 (-0.32, -0.152)
Unadjusted mean TC (calculated over restricted cholesterol range: 4.0-7.5 mmol/L)	-0.378 (-0.507, -0.248)
Mean HDL cholesterol	0.956 (0.828, 1.08)
Unadjusted mean non-HDL cholesterol * mean age of age group	0.000485 (-0.000117, 0.00109)
Unadjusted mean non-HDL cholesterol * male sex	0.0517 (0.0307, 0.0727)
Number of data points used to fit the model = 3,814	
R^2 = 0.954	

* Total cholesterol was measured using the portable device Accutrend and HDL cholesterol was measured in a laboratory.

Conversion regression for Multicare (non-HDL cholesterol)*	
**Variables**	**Coefficients (95% CI)**
Intercept	-0.863 (-0.924, -0.803)
Unadjusted mean non-HDL cholesterol (calculated as TC minus HDL cholesterol, using only TC values within restricted cholesterol range: 3.5-10.0 mmol/L)	1.59 (1.52, 1.67)
Mean age of age group	0.00406 (0.00306, 0.00506)
Male sex	-0.185 (-0.221, -0.149)
Unadjusted mean TC (calculated over restricted cholesterol range: 3.5-10.0 mmol/L)	-0.453 (-0.526, -0.379)
Mean HDL cholesterol	0.648 (0.574, 0.723)
Unadjusted mean non-HDL cholesterol * mean age of age group	-0.00111 (-0.00137, -0.000839)
Unadjusted mean non-HDL cholesterol * male sex	0.0442 (0.035, 0.0533)
Number of data points used to fit the model = 3,927	
R^2 = 0.984	

* Total cholesterol was measured using the portable device Multicare and HDL cholesterol was measured in a laboratory.

Conversion regression for CardioChek, Cholestech, Multicare and Reflotron (HDL cholesterol)	
**Variables**	**Coefficients (95% CI)**
Intercept	-0.0441 (-0.061, -0.0273)
Unadjusted mean HDL cholesterol (calculated over restricted cholesterol range: 0.4-2.4 mmol/L)	1.04 (1.03, 1.05)
Mean age of age group	-0.00101 (-0.0013, -0.000723)
Male sex	0.05 (0.038, 0.0619)
Unadjusted mean HDL cholesterol * mean age of age group	0.000923 (0.000707, 0.00114)
Unadjusted mean HDL cholesterol * male sex	-0.0379 (-0.0471, -0.0288)
Number of data points used to fit the model = 3,997	
R^2 = 0.986	

Conversion regression for LipidoCare (HDL cholesterol)	
**Variables**	**Coefficients (95% CI)**
Intercept	-0.241 (-0.267, -0.214)
Unadjusted mean HDL cholesterol (calculated over restricted cholesterol range: 0.8-2.3 mmol/L)	1.17 (1.15, 1.19)
Mean age of age group	-0.00152 (-0.00198, -0.00106)
Male sex	-0.0225 (-0.0423, -0.00273)
Unadjusted mean HDL cholesterol * mean age of age group	0.00125 (0.000909, 0.00159)
Unadjusted mean HDL cholesterol * male sex	0.0223 (0.00732, 0.0373)
Number of data points used to fit the model = 3,962	
R^2 = 0.974	

Supplementary Table 5. Results of model validation.

Mean TC, Test 1, Women											
**Data**		**No. of held out observations**	**Percent covered**	**Error (mmol/L)†**				**Absolute error (mmol/L)**			
				**Median**	**Q1**	**Q3**	**(p*)**	**Median**	**Q1**	**Q3**	**(p*)**
All		2,870	91	-0.04	-0.26	0.17	0.0000	0.21	0.10	0.39	0.0000
Super-region	Central and eastern Europe	288	90	-0.01	-0.21	0.23	0.8661	0.23	0.11	0.40	0.8661
	Central Asia, Middle East and north Africa	573	86	-0.10	-0.36	0.10	0.0000	0.24	0.10	0.45	0.0000
	East and southeast Asia	85	91	0.26	-0.04	0.45	0.0000	0.30	0.17	0.45	0.0000
	High-income Asia Pacific	159	99	0.12	0.04	0.26	0.0000	0.15	0.07	0.26	0.0000
	High-income western countries	1,071	95	-0.07	-0.24	0.13	0.0000	0.19	0.10	0.34	0.0000
	Latin America and the Caribbean	241	94	0.02	-0.20	0.19	0.6557	0.19	0.09	0.33	0.6557
	Oceania	159	75	-0.03	-0.34	0.25	0.4194	0.31	0.11	0.58	0.4194
	South Asia	135	88	-0.08	-0.43	0.14	0.0014	0.25	0.13	0.52	0.0014
	Sub-Saharan Africa	159	82	-0.13	-0.39	0.13	0.0002	0.28	0.13	0.53	0.0002
Urban or rural studies	Rural	300	82	-0.15	-0.42	0.15	0.0000	0.29	0.15	0.57	0.0000
	Urban	914	90	-0.02	-0.24	0.19	0.0096	0.21	0.10	0.40	0.0096
	Both rural and urban	1,656	92	-0.03	-0.24	0.16	0.0000	0.20	0.09	0.37	0.0000
Study representativeness	Community	847	86	-0.07	-0.34	0.18	0.0000	0.26	0.11	0.47	0.0000
	Subnational	708	90	-0.10	-0.30	0.11	0.0000	0.22	0.10	0.40	0.0000
	National	1,315	94	0.01	-0.18	0.19	0.7346	0.19	0.09	0.34	0.7346
Age band	18-40	940	92	-0.01	-0.18	0.15	0.0501	0.17	0.09	0.32	0.0501
	40-60	925	89	-0.05	-0.27	0.15	0.0000	0.21	0.09	0.38	0.0000
	60 and above	1,005	91	-0.05	-0.31	0.21	0.0000	0.26	0.12	0.47	0.0000
Years	1980-1989	275	85	-0.09	-0.39	0.24	0.0056	0.33	0.16	0.48	0.0056
	1990-1999	484	89	-0.05	-0.32	0.17	0.0003	0.24	0.10	0.43	0.0003
	2000-2009	1,112	89	-0.06	-0.27	0.16	0.0000	0.22	0.10	0.42	0.0000
	2010-2018	999	95	-0.01	-0.18	0.17	0.1785	0.18	0.08	0.31	0.1785
Data density	Data poor	165	90	0.10	-0.12	0.34	0.0009	0.26	0.11	0.43	0.0009
	Average data density	471	86	-0.01	-0.25	0.23	0.6390	0.24	0.11	0.44	0.6390
	Data rich	2,234	92	-0.05	-0.26	0.15	0.0000	0.20	0.10	0.38	0.0000

Mean TC, Test 1, Men												
**Data**		**No. of held out observations**	**Percent covered**	**Error (mmol/L)†**				**Absolute error (mmol/L)**			
				**Median**	**Q1**	**Q3**	**(p*)**	**Median**	**Q1**	**Q3**	**(p*)**
All		2,853	92	0.03	-0.19	0.27	0.0000	0.23	0.11	0.41	0.0000
Super-region	Central and eastern Europe	205	91	0.05	-0.25	0.31	0.3030	0.29	0.16	0.46	0.3030
	Central Asia, Middle East and north Africa	293	95	0.02	-0.21	0.25	0.3192	0.23	0.11	0.38	0.3192
	East and southeast Asia	211	80	-0.01	-0.26	0.16	0.1600	0.21	0.10	0.54	0.1600
	High-income Asia Pacific	*No data from this region were among the held-out data*									
	High-income western countries	1,531	95	0.07	-0.14	0.30	0.0000	0.21	0.10	0.37	0.0000
	Latin America and the Caribbean	348	95	0.03	-0.17	0.24	0.2182	0.22	0.09	0.44	0.2182
	Oceania	146	74	-0.14	-0.53	0.28	0.0332	0.40	0.20	0.69	0.0332
	South Asia	26	100	0.08	-0.22	0.29	0.3032	0.26	0.13	0.38	0.3032
	Sub-Saharan Africa	93	84	-0.16	-0.56	0.09	0.0001	0.25	0.15	0.56	0.0001
Urban or rural studies	Rural	315	90	0.04	-0.23	0.36	0.0335	0.30	0.15	0.50	0.0335
	Urban	878	92	0.08	-0.13	0.28	0.0000	0.21	0.10	0.38	0.0000
	Both rural and urban	1,660	92	0.01	-0.21	0.25	0.0479	0.23	0.10	0.39	0.0479
Study representativeness	Community	937	92	0.08	-0.17	0.31	0.0000	0.25	0.12	0.42	0.0000
	Subnational	734	90	0.01	-0.19	0.26	0.0058	0.22	0.10	0.44	0.0058
	National	1,182	93	0.02	-0.20	0.24	0.1098	0.23	0.10	0.38	0.1098
Age band	18-40	898	92	0.03	-0.16	0.26	0.0003	0.21	0.10	0.38	0.0003
	40-60	895	89	0.00	-0.22	0.21	0.8472	0.21	0.10	0.39	0.8472
	60 and above	1,060	94	0.07	-0.18	0.33	0.0000	0.26	0.12	0.44	0.0000
Years	1980-1989	359	91	0.07	-0.18	0.30	0.0034	0.26	0.13	0.43	0.0034
	1990-1999	601	90	0.01	-0.22	0.28	0.0575	0.25	0.11	0.42	0.0575
	2000-2009	1,031	91	0.05	-0.16	0.28	0.0000	0.22	0.10	0.40	0.0000
	2010-2018	862	95	0.00	-0.20	0.24	0.1769	0.22	0.10	0.39	0.1769
Data density	Data poor	165	91	0.10	-0.17	0.34	0.0335	0.26	0.15	0.48	0.0335
	Average data density	420	88	-0.05	-0.36	0.22	0.0061	0.29	0.16	0.50	0.0061
	Data rich	2,268	93	0.04	-0.16	0.27	0.0000	0.22	0.10	0.38	0.0000

Mean TC, Test 2, Women											
**Data**		**No. of held out observations**	**Percent covered**	**Error (mmol/L)†**				**Absolute error (mmol/L)**			
				**Median**	**Q1**	**Q3**	**(p*)**	**Median**	**Q1**	**Q3**	**(p*)**
All		2,971	83	-0.04	-0.23	0.16	0.0000	0.20	0.09	0.35	0.0000
Super-region	Central and eastern Europe	195	89	-0.18	-0.30	-0.01	0.0000	0.23	0.14	0.35	0.0000
	Central Asia, Middle East and north Africa	407	78	-0.02	-0.22	0.21	0.7609	0.22	0.10	0.41	0.7609
	East and southeast Asia	264	74	-0.08	-0.30	0.13	0.0002	0.21	0.10	0.47	0.0002
	High-income Asia Pacific	353	92	-0.08	-0.23	0.09	0.0000	0.17	0.09	0.30	0.0000
	High-income western countries	1,132	85	-0.02	-0.17	0.15	0.1350	0.16	0.07	0.31	0.1350
	Latin America and the Caribbean	289	87	-0.03	-0.26	0.21	0.3297	0.24	0.12	0.40	0.3297
	Oceania	117	76	-0.06	-0.34	0.32	0.7074	0.34	0.15	0.55	0.7074
	South Asia	119	78	0.09	-0.10	0.31	0.0029	0.27	0.09	0.42	0.0029
	Sub-Saharan Africa	95	75	-0.02	-0.34	0.23	0.4590	0.30	0.14	0.74	0.4590
Urban or rural studies	Rural	357	80	-0.02	-0.25	0.23	0.5027	0.24	0.10	0.45	0.5027
	Urban	804	81	0.00	-0.19	0.20	0.7213	0.19	0.08	0.36	0.7213
	Both rural and urban	1,810	85	-0.06	-0.24	0.14	0.0000	0.20	0.10	0.34	0.0000
Study representativeness	Community	911	81	-0.02	-0.23	0.19	0.0567	0.22	0.09	0.38	0.0567
	Subnational	713	84	-0.03	-0.17	0.17	0.3497	0.17	0.08	0.33	0.3497
	National	1,347	85	-0.06	-0.26	0.14	0.0000	0.20	0.10	0.34	0.0000
Age band	18-40	922	82	-0.08	-0.25	0.10	0.0000	0.19	0.09	0.33	0.0000
	40-60	916	82	-0.05	-0.23	0.14	0.0000	0.19	0.09	0.34	0.0000
	60 and above	1,133	86	0.01	-0.19	0.23	0.0973	0.21	0.10	0.40	0.0973
Years	1980-1989	208	69	-0.01	-0.17	0.28	0.5523	0.24	0.09	0.45	0.5523
	1990-1999	232	75	0.01	-0.18	0.20	0.4562	0.19	0.10	0.37	0.4562
	2000-2009	1,507	82	-0.04	-0.22	0.17	0.0007	0.20	0.09	0.35	0.0007
	2010-2018	1,024	90	-0.06	-0.24	0.13	0.0000	0.20	0.09	0.34	0.0000
Data density	Average data density	446	83	-0.04	-0.24	0.24	0.9675	0.24	0.11	0.42	0.9675
	Data rich	2,525	83	-0.04	-0.23	0.15	0.0000	0.19	0.09	0.34	0.0000
Hold out pattern	Post-2000 data removed	1,519	87	-0.06	-0.25	0.13	0.0000	0.19	0.09	0.34	0.0000
	Random set of data removed	1,452	80	-0.02	-0.20	0.21	0.7688	0.21	0.10	0.37	0.7688

Mean TC, Test 2, Men											
Data		No. of held out observations	Percent covered	Error (mmol/L)†				Absolute error (mmol/L)			
				Median	Q1	Q3	(p*)	Median	Q1	Q3	(p*)
All		2,921	82	-0.03	-0.23	0.17	0.0000	0.20	0.09	0.36	0.0000
Super-region	Central and eastern Europe	223	83	-0.16	-0.35	0.09	0.0000	0.24	0.12	0.40	0.0000
	Central Asia, Middle East and north Africa	381	83	-0.08	-0.27	0.11	0.0000	0.19	0.09	0.35	0.0000
	East and southeast Asia	243	72	0.03	-0.19	0.22	0.2802	0.20	0.11	0.46	0.2802
	High-income Asia Pacific	290	93	0.00	-0.09	0.13	0.1158	0.11	0.06	0.20	0.1158
	High-income western countries	1,137	84	-0.01	-0.19	0.19	0.7688	0.19	0.09	0.32	0.7688
	Latin America and the Caribbean	314	86	-0.09	-0.27	0.17	0.0072	0.24	0.12	0.41	0.0072
	Oceania	103	69	-0.06	-0.38	0.27	0.2241	0.32	0.16	0.58	0.2241
	South Asia	143	66	0.02	-0.38	0.26	0.3390	0.29	0.18	0.54	0.3390
	Sub-Saharan Africa	87	68	-0.08	-0.35	0.32	0.4349	0.34	0.16	0.61	0.4349
Urban or rural studies	Rural	340	78	-0.03	-0.26	0.20	0.1302	0.23	0.10	0.45	0.1302
	Urban	765	79	0.01	-0.23	0.20	0.5382	0.21	0.10	0.37	0.5382
	Both rural and urban	1,816	84	-0.05	-0.22	0.16	0.0000	0.19	0.09	0.34	0.0000
Study representativeness	Community	971	80	-0.03	-0.25	0.17	0.0004	0.21	0.10	0.37	0.0004
	Subnational	641	78	-0.04	-0.23	0.16	0.0134	0.21	0.09	0.39	0.0134
	National	1,309	85	-0.03	-0.20	0.18	0.0832	0.19	0.09	0.34	0.0832
Age band	18-40	902	80	-0.07	-0.25	0.14	0.0000	0.20	0.10	0.34	0.0000
	40-60	909	82	-0.05	-0.23	0.13	0.0000	0.19	0.09	0.34	0.0000
	60 and above	1,110	84	0.01	-0.19	0.23	0.1308	0.21	0.10	0.40	0.1308
Years	1980-1989	268	77	0.08	-0.14	0.23	0.0063	0.20	0.09	0.37	0.0063
	1990-1999	360	81	-0.03	-0.20	0.16	0.1780	0.18	0.09	0.35	0.1780
	2000-2009	1,356	81	-0.02	-0.21	0.18	0.0531	0.20	0.09	0.35	0.0531
	2010-2018	937	86	-0.08	-0.26	0.14	0.0000	0.21	0.10	0.37	0.0000
Data density	Average data density	467	78	-0.09	-0.35	0.17	0.0000	0.29	0.13	0.49	0.0000
	Data rich	2,454	83	-0.02	-0.20	0.17	0.0071	0.19	0.09	0.33	0.0071
Hold out pattern	Post-2000 data removed	875	84	-0.03	-0.24	0.22	0.0773	0.23	0.12	0.39	0.0773
	Random set of data removed	2,046	81	-0.03	-0.21	0.15	0.0000	0.18	0.09	0.35	0.0000

Mean non-HDL cholesterol, Test 1, Women											
Data		No. of held out observations	Percent covered	Error (mmol/L)†				Absolute error (mmol/L)			
				Median	Q1	Q3	(p*)	Median	Q1	Q3	(p*)
All		2,333	83	0.03	-0.14	0.24	0.0000	0.19	0.08	0.36	0.0000
Super-region	Central and eastern Europe	195	93	0.04	-0.10	0.17	0.0193	0.15	0.07	0.25	0.0193
	Central Asia, Middle East and north Africa	265	91	0.00	-0.16	0.20	0.4336	0.17	0.08	0.30	0.4336
	East and southeast Asia	101	86	0.08	-0.23	0.25	0.1754	0.24	0.14	0.38	0.1754
	High-income Asia Pacific	469	51	0.47	0.03	0.69	0.0000	0.47	0.18	0.69	0.0000
	High-income western countries	1,010	91	-0.02	-0.16	0.12	0.0055	0.15	0.07	0.27	0.0055
	Latin America and the Caribbean	152	93	0.01	-0.16	0.16	0.7819	0.16	0.06	0.30	0.7819
	Oceania	52	75	0.00	-0.26	0.31	0.9528	0.27	0.11	0.46	0.9528
	South Asia	39	90	-0.08	-0.22	0.01	0.0268	0.16	0.08	0.36	0.0268
	Sub-Saharan Africa	50	86	-0.11	-0.28	0.07	0.0053	0.22	0.09	0.36	0.0053
Urban or rural studies	Rural	261	57	0.19	-0.17	0.84	0.0000	0.42	0.17	0.84	0.0000
	Urban	685	91	0.01	-0.14	0.19	0.0309	0.16	0.07	0.28	0.0309
	Both rural and urban	1,387	84	0.02	-0.13	0.23	0.0000	0.18	0.08	0.36	0.0000
Study representativeness	Community	664	75	0.06	-0.15	0.38	0.0000	0.23	0.11	0.50	0.0000
	Subnational	565	89	-0.02	-0.16	0.13	0.1110	0.14	0.07	0.28	0.1110
	National	1,104	84	0.04	-0.11	0.27	0.0000	0.19	0.07	0.35	0.0000
Age band	18-40	718	89	0.05	-0.09	0.23	0.0000	0.16	0.07	0.31	0.0000
	40-60	736	75	0.02	-0.12	0.23	0.0000	0.17	0.07	0.35	0.0000
	60 and above	879	85	0.01	-0.18	0.29	0.0003	0.21	0.09	0.44	0.0003
Years	1980-1989	262	79	0.07	-0.11	0.28	0.0000	0.20	0.08	0.41	0.0000
	1990-1999	459	63	0.15	-0.09	0.57	0.0000	0.31	0.12	0.62	0.0000
	2000-2009	863	85	0.03	-0.13	0.24	0.0000	0.18	0.08	0.35	0.0000
	2010-2018	749	94	-0.03	-0.17	0.13	0.0094	0.15	0.07	0.27	0.0094
Data density	Data poor	153	93	0.03	-0.15	0.20	0.3682	0.17	0.08	0.28	0.3682
	Average data density	316	84	-0.04	-0.23	0.21	0.4483	0.22	0.10	0.36	0.4483
	Data rich	1,864	82	0.03	-0.12	0.27	0.0000	0.18	0.08	0.37	0.0000

Mean non-HDL cholesterol, Test 1, Men											
**Data**		**No. of held out observations**	**Percent covered**	**Error (mmol/L)†**				**Absolute error (mmol/L)**			
				**Median**	**Q1**	**Q3**	**(p*)**	**Median**	**Q1**	**Q3**	**(p*)**
All		2,367	89	0.05	-0.13	0.21	0.0000	0.18	0.08	0.32	0.0000
Super-region	Central and eastern Europe	265	89	0.11	-0.02	0.26	0.0000	0.16	0.07	0.32	0.0000
	Central Asia, Middle East and north Africa	519	89	-0.05	-0.23	0.13	0.0000	0.19	0.09	0.32	0.0000
	East and southeast Asia	66	77	-0.03	-0.26	0.31	0.8731	0.29	0.14	0.43	0.8731
	High-income Asia Pacific	132	100	0.02	-0.08	0.12	0.3582	0.11	0.06	0.15	0.3582
	High-income western countries	1,048	90	0.10	-0.07	0.27	0.0000	0.18	0.08	0.32	0.0000
	Latin America and the Caribbean	211	93	-0.01	-0.19	0.14	0.1870	0.16	0.09	0.26	0.1870
	Oceania	54	69	-0.04	-0.29	0.37	0.9931	0.35	0.16	0.57	0.9931
	South Asia	*No data from this region were among the held-out data*									
	Sub-Saharan Africa	72	76	0.01	-0.32	0.21	0.1485	0.25	0.15	0.42	0.1485
Urban or rural studies	Rural	359	91	0.00	-0.19	0.20	0.6685	0.19	0.09	0.33	0.6685
	Urban	625	89	0.04	-0.15	0.21	0.0031	0.18	0.08	0.31	0.0031
	Both rural and urban	1,383	89	0.05	-0.11	0.21	0.0000	0.17	0.08	0.31	0.0000
Study representativeness	Community	877	89	0.03	-0.16	0.19	0.0262	0.18	0.08	0.32	0.0262
	Subnational	423	86	0.05	-0.14	0.22	0.0060	0.19	0.09	0.33	0.0060
	National	1,067	91	0.06	-0.10	0.21	0.0000	0.16	0.08	0.31	0.0000
Age band	18-40	742	92	0.03	-0.14	0.18	0.0113	0.16	0.08	0.29	0.0113
	40-60	746	85	0.04	-0.13	0.18	0.0016	0.16	0.08	0.27	0.0016
	60 and above	879	91	0.08	-0.12	0.27	0.0000	0.21	0.10	0.37	0.0000
Years	1980-1989	257	85	0.08	-0.11	0.23	0.0001	0.18	0.09	0.32	0.0001
	1990-1999	298	90	0.07	-0.08	0.22	0.0000	0.17	0.08	0.28	0.0000
	2000-2009	983	89	0.04	-0.14	0.22	0.0000	0.19	0.08	0.33	0.0000
	2010-2018	829	91	0.03	-0.15	0.18	0.0067	0.16	0.09	0.31	0.0067
Data density	Data poor	162	90	-0.06	-0.24	0.19	0.0575	0.21	0.11	0.35	0.0575
	Average data density	330	82	0.01	-0.21	0.23	0.5580	0.22	0.11	0.40	0.5580
	Data rich	1,875	91	0.05	-0.11	0.21	0.0000	0.17	0.08	0.30	0.0000

Mean non-HDL cholesterol, Test 2, Women											
Data		No. of held out observations	Percent covered	Error (mmol/L)†				Absolute error (mmol/L)			
				Median	Q1	Q3	(p*)	Median	Q1	Q3	(p*)
All		2,261	81	0.01	-0.14	0.17	0.0342	0.16	0.07	0.29	0.0342
Super-region	Central and eastern Europe	197	88	0.05	-0.07	0.16	0.0008	0.13	0.06	0.25	0.0008
	Central Asia, Middle East and north Africa	326	73	0.01	-0.15	0.22	0.1930	0.18	0.08	0.33	0.1930
	East and southeast Asia	183	64	0.11	-0.19	0.34	0.0051	0.29	0.13	0.49	0.0051
	High-income Asia Pacific	241	94	0.00	-0.07	0.08	0.8826	0.08	0.04	0.15	0.8826
	High-income western countries	919	83	0.00	-0.14	0.15	0.3720	0.15	0.07	0.25	0.3720
	Latin America and the Caribbean	207	87	-0.04	-0.21	0.16	0.0294	0.19	0.08	0.32	0.0294
	Oceania	40	63	0.30	0.17	0.48	0.0000	0.33	0.21	0.49	0.0000
	South Asia	119	77	-0.05	-0.29	0.13	0.1458	0.21	0.09	0.40	0.1458
	Sub-Saharan Africa	29	62	-0.22	-0.80	0.01	0.0006	0.29	0.13	0.80	0.0006
Urban or rural studies	Rural	238	78	0.01	-0.21	0.21	0.9587	0.21	0.09	0.36	0.9587
	Urban	697	84	0.03	-0.11	0.18	0.0004	0.15	0.07	0.26	0.0004
	Both rural and urban	1,326	80	0.00	-0.15	0.15	0.7615	0.15	0.06	0.29	0.7615
Study representativeness	Community	755	80	0.03	-0.15	0.19	0.0237	0.18	0.08	0.31	0.0237
	Subnational	491	83	0.01	-0.13	0.17	0.1183	0.15	0.06	0.26	0.1183
	National	1,015	81	0.00	-0.14	0.14	0.9941	0.14	0.06	0.29	0.9941
Age band	18-40	705	81	-0.01	-0.16	0.13	0.1500	0.15	0.07	0.27	0.1500
	40-60	712	81	0.00	-0.13	0.15	0.6016	0.14	0.07	0.26	0.6016
	60 and above	844	81	0.04	-0.13	0.22	0.0001	0.18	0.08	0.35	0.0001
Years	1980-1989	254	89	0.03	-0.10	0.17	0.0029	0.14	0.06	0.22	0.0029
	1990-1999	202	75	0.07	-0.12	0.20	0.0116	0.17	0.09	0.29	0.0116
	2000-2009	984	78	0.01	-0.15	0.18	0.1611	0.17	0.07	0.34	0.1611
	2010-2018	821	84	-0.01	-0.16	0.14	0.3072	0.15	0.07	0.28	0.3072
Data density	Average data density	365	73	-0.04	-0.25	0.21	0.2208	0.23	0.10	0.41	0.2208
	Data rich	1,896	83	0.02	-0.12	0.16	0.0016	0.14	0.07	0.27	0.0016
Hold out pattern	Post-2000 data removed	683	82	0.04	-0.10	0.22	0.0000	0.16	0.07	0.34	0.0000
	Random set of data removed	1,578	81	0.00	-0.16	0.15	0.4815	0.15	0.07	0.27	0.4815

Mean non-HDL cholesterol, Test 2, Men												
Data		No. of held out observations	Percent covered	Error (mmol/L)†				Absolute error (mmol/L)			
				Median	Q1	Q3	(p*)	Median	Q1	Q3	(p*)
All		2,421	77	0.00	-0.17	0.18	0.7216	0.17	0.08	0.31	0.7216
Super-region	Central and eastern Europe	228	85	0.02	-0.13	0.15	0.3976	0.14	0.07	0.27	0.3976
	Central Asia, Middle East and north Africa	314	74	-0.02	-0.21	0.19	0.5000	0.20	0.09	0.34	0.5000
	East and southeast Asia	222	64	0.05	-0.23	0.26	0.3250	0.25	0.13	0.38	0.3250
	High-income Asia Pacific	249	91	0.00	-0.09	0.10	0.9965	0.10	0.05	0.19	0.9965
	High-income western countries	1,018	78	0.02	-0.14	0.18	0.0245	0.16	0.08	0.28	0.0245
	Latin America and the Caribbean	264	83	-0.04	-0.20	0.15	0.1148	0.18	0.10	0.35	0.1148
	Oceania	40	58	0.24	-0.25	0.49	0.0408	0.43	0.26	0.59	0.0408
	South Asia	66	47	-0.33	-0.67	-0.01	0.0000	0.45	0.18	0.73	0.0000
	Sub-Saharan Africa	20	60	-0.15	-0.66	0.23	0.0826	0.35	0.19	0.66	0.0826
Urban or rural studies	Rural	326	74	0.01	-0.24	0.22	0.5434	0.23	0.11	0.39	0.5434
	Urban	712	77	0.01	-0.15	0.20	0.2143	0.17	0.09	0.32	0.2143
	Both rural and urban	1,383	78	-0.01	-0.17	0.16	0.8222	0.16	0.07	0.29	0.8222
Study representativeness	Community	896	77	0.00	-0.18	0.20	0.9364	0.19	0.09	0.34	0.9364
	Subnational	513	80	0.02	-0.13	0.19	0.0093	0.16	0.07	0.28	0.0093
	National	1,012	76	-0.01	-0.18	0.15	0.1779	0.17	0.08	0.31	0.1779
Age band	18-40	742	74	-0.04	-0.20	0.15	0.0014	0.18	0.09	0.32	0.0014
	40-60	770	77	-0.01	-0.17	0.15	0.3484	0.16	0.07	0.29	0.3484
	60 and above	909	80	0.05	-0.13	0.21	0.0000	0.18	0.08	0.34	0.0000
Years	1980-1989	257	75	0.00	-0.17	0.19	0.9799	0.18	0.09	0.30	0.9799
	1990-1999	270	79	0.00	-0.15	0.19	0.2665	0.16	0.09	0.27	0.2665
	2000-2009	1,076	73	0.00	-0.17	0.19	0.5753	0.18	0.08	0.35	0.5753
	2010-2018	818	83	0.00	-0.17	0.15	0.4592	0.16	0.07	0.28	0.4592
Data density	Average data density	340	70	0.01	-0.26	0.25	0.9954	0.25	0.12	0.44	0.9954
	Data rich	2,081	79	0.00	-0.16	0.16	0.6968	0.16	0.08	0.29	0.6968
Hold out pattern	Post-2000 data removed	760	76	-0.01	-0.21	0.20	0.4137	0.20	0.10	0.35	0.4137
	Random set of data removed	1,661	78	0.01	-0.15	0.17	0.3000	0.16	0.07	0.30	0.3000

Mean HDL cholesterol, Test 1, Women											
**Data**		**No. of held out observations**	**Percent covered**	**Error (mmol/L)†**				**Absolute error (mmol/L)**			
				**Median**	**Q1**	**Q3**	**(p*)**	**Median**	**Q1**	**Q3**	**(p*)**
All		2,349	90	0.01	-0.08	0.11	0.0000	0.09	0.05	0.16	0.0000
Super-region	Central and eastern Europe	77	99	-0.05	-0.12	0.04	0.0226	0.09	0.05	0.14	0.0226
	Central Asia, Middle East and north Africa	387	83	0.07	-0.05	0.15	0.0000	0.12	0.06	0.19	0.0000
	East and southeast Asia	124	87	-0.02	-0.10	0.11	0.6817	0.11	0.05	0.17	0.6817
	High-income Asia Pacific	132	85	0.13	0.05	0.24	0.0000	0.13	0.06	0.24	0.0000
	High-income western countries	1,268	92	-0.03	-0.09	0.06	0.0000	0.08	0.04	0.14	0.0000
	Latin America and the Caribbean	141	98	0.03	-0.05	0.09	0.0131	0.07	0.04	0.13	0.0131
	Oceania	42	86	0.03	-0.06	0.17	0.4646	0.11	0.05	0.23	0.4646
	South Asia	120	76	0.16	0.11	0.24	0.0000	0.17	0.12	0.24	0.0000
	Sub-Saharan Africa	58	100	0.03	-0.01	0.14	0.0009	0.06	0.03	0.14	0.0009
Urban or rural studies	Rural	215	83	0.06	-0.02	0.15	0.0000	0.10	0.05	0.18	0.0000
	Urban	729	89	0.02	-0.07	0.12	0.0002	0.10	0.05	0.17	0.0002
	Both rural and urban	1,405	91	0.00	-0.08	0.10	0.3767	0.09	0.04	0.15	0.3767
Study representativeness	Community	627	88	0.05	-0.03	0.14	0.0000	0.10	0.05	0.17	0.0000
	Subnational	714	88	0.00	-0.09	0.09	0.5538	0.09	0.04	0.17	0.5538
	National	1,008	93	-0.01	-0.08	0.10	0.5867	0.09	0.04	0.16	0.5867
Age band	18-40	766	91	0.03	-0.06	0.12	0.0000	0.09	0.04	0.16	0.0000
	40-60	722	89	0.01	-0.08	0.10	0.1002	0.09	0.05	0.16	0.1002
	60 and above	861	89	0.00	-0.09	0.11	0.4448	0.10	0.05	0.18	0.4448
Years	1980-1989	223	82	-0.01	-0.11	0.10	0.5766	0.10	0.05	0.17	0.5766
	1990-1999	290	91	-0.01	-0.09	0.08	0.7555	0.09	0.04	0.15	0.7555
	2000-2009	939	88	0.02	-0.06	0.13	0.0000	0.10	0.05	0.17	0.0000
	2010-2018	897	93	0.01	-0.08	0.10	0.0762	0.09	0.04	0.16	0.0762
Data density	Data poor	158	94	0.04	-0.08	0.14	0.0158	0.11	0.06	0.18	0.0158
	Average data density	305	88	0.00	-0.10	0.08	0.7629	0.09	0.05	0.16	0.7629
	Data rich	1,886	90	0.01	-0.07	0.11	0.0000	0.09	0.04	0.16	0.0000

Mean HDL cholesterol, Test 1, Men												
Data		No. of held out observations	Percent covered	Error (mmol/L)†				Absolute error (mmol/L)			
				Median	Q1	Q3	(p*)	Median	Q1	Q3	(p*)
All		2,382	92	0.00	-0.08	0.08	0.9468	0.08	0.04	0.13	0.9468
Super-region	Central and eastern Europe	145	91	-0.05	-0.10	0.05	0.0026	0.09	0.05	0.14	0.0026
	Central Asia, Middle East and north Africa	332	86	0.02	-0.05	0.11	0.0009	0.08	0.03	0.16	0.0009
	East and southeast Asia	146	99	-0.04	-0.11	0.03	0.0000	0.08	0.04	0.12	0.0000
	High-income Asia Pacific	132	99	0.03	-0.04	0.07	0.0048	0.06	0.03	0.10	0.0048
	High-income western countries	1,246	94	-0.01	-0.07	0.07	0.1985	0.07	0.04	0.12	0.1985
	Latin America and the Caribbean	241	91	0.04	-0.04	0.11	0.0000	0.07	0.04	0.14	0.0000
	Oceania	91	59	-0.09	-0.26	0.12	0.0068	0.19	0.12	0.31	0.0068
	South Asia	6	83	-0.16	-0.16	-0.15	0.0313	0.16	0.15	0.16	0.0313
	Sub-Saharan Africa	43	95	0.00	-0.09	0.15	0.2670	0.11	0.07	0.16	0.2670
Urban or rural studies	Rural	309	89	0.03	-0.06	0.12	0.0010	0.09	0.04	0.16	0.0010
	Urban	661	93	0.01	-0.06	0.10	0.0000	0.08	0.04	0.13	0.0000
	Both rural and urban	1,412	92	-0.02	-0.09	0.06	0.0000	0.07	0.04	0.13	0.0000
Study representativeness	Community	889	90	0.02	-0.06	0.11	0.0000	0.08	0.04	0.15	0.0000
	Subnational	432	94	-0.01	-0.07	0.07	0.2245	0.07	0.03	0.12	0.2245
	National	1,061	92	-0.02	-0.09	0.06	0.0000	0.07	0.04	0.12	0.0000
Age band	18-40	742	92	-0.01	-0.08	0.07	0.0360	0.08	0.04	0.13	0.0360
	40-60	735	90	0.00	-0.08	0.07	0.6052	0.08	0.04	0.13	0.6052
	60 and above	905	93	0.01	-0.07	0.09	0.0151	0.08	0.04	0.14	0.0151
Years	1980-1989	245	85	-0.01	-0.08	0.08	0.6952	0.08	0.04	0.16	0.6952
	1990-1999	326	87	0.00	-0.07	0.10	0.1208	0.08	0.04	0.14	0.1208
	2000-2009	1,015	94	-0.01	-0.08	0.08	0.6163	0.08	0.04	0.13	0.6163
	2010-2018	796	92	0.01	-0.08	0.07	0.9824	0.07	0.04	0.13	0.9824
Data density	Data poor	161	88	0.05	-0.07	0.17	0.0000	0.12	0.06	0.19	0.0000
	Average data density	302	85	-0.05	-0.14	0.05	0.0000	0.10	0.05	0.17	0.0000
	Data rich	1,919	93	0.00	-0.07	0.08	0.2054	0.07	0.03	0.12	0.2054

Mean HDL cholesterol, Test 2, Women												
**Data**		**No. of held out observations**	**Percent covered**	**Error (mmol/L)†**				**Absolute error (mmol/L)**			
				**Median**	**Q1**	**Q3**	**(p*)**	**Median**	**Q1**	**Q3**	**(p*)**
All		2,437	75	0.00	-0.08	0.08	0.4522	0.08	0.04	0.15	0.4522
Super-region	Central and eastern Europe	189	75	0.02	-0.06	0.10	0.0726	0.08	0.04	0.14	0.0726
	Central Asia, Middle East and north Africa	286	66	0.00	-0.08	0.07	0.6409	0.08	0.04	0.17	0.6409
	East and southeast Asia	216	73	0.00	-0.08	0.09	0.8147	0.08	0.03	0.14	0.8147
	High-income Asia Pacific	245	84	-0.02	-0.09	0.05	0.0004	0.06	0.03	0.10	0.0004
	High-income western countries	1,092	72	0.01	-0.08	0.09	0.1257	0.09	0.04	0.16	0.1257
	Latin America and the Caribbean	221	88	-0.03	-0.10	0.05	0.0003	0.07	0.04	0.14	0.0003
	Oceania	40	65	-0.14	-0.24	-0.04	0.0000	0.15	0.06	0.24	0.0000
	South Asia	120	73	0.11	0.03	0.19	0.0000	0.13	0.06	0.19	0.0000
	Sub-Saharan Africa	28	93	0.01	-0.07	0.05	0.8842	0.06	0.04	0.10	0.8842
Urban or rural studies	Rural	299	69	0.02	-0.08	0.13	0.0233	0.10	0.05	0.19	0.0233
	Urban	687	67	0.03	-0.08	0.11	0.0002	0.10	0.05	0.16	0.0002
	Both rural and urban	1,451	79	-0.01	-0.08	0.06	0.0014	0.07	0.04	0.13	0.0014
Study representativeness	Community	822	72	0.02	-0.07	0.10	0.0003	0.08	0.04	0.16	0.0003
	Subnational	529	70	0.02	-0.09	0.10	0.1156	0.09	0.05	0.16	0.1156
	National	1,086	79	-0.02	-0.09	0.06	0.0009	0.08	0.04	0.13	0.0009
Age band	18-40	771	74	0.02	-0.06	0.10	0.0000	0.08	0.04	0.15	0.0000
	40-60	748	73	0.00	-0.09	0.07	0.5525	0.08	0.04	0.14	0.5525
	60 and above	918	77	-0.01	-0.09	0.07	0.0183	0.08	0.04	0.15	0.0183
Years	1980-1989	184	60	0.02	-0.08	0.11	0.2408	0.09	0.05	0.18	0.2408
	1990-1999	221	71	0.02	-0.07	0.08	0.1157	0.07	0.04	0.14	0.1157
	2000-2009	1,188	73	0.00	-0.08	0.08	0.9607	0.08	0.04	0.14	0.9607
	2010-2018	844	81	0.00	-0.08	0.08	0.8982	0.08	0.04	0.14	0.8982
Data density	Average data density	365	80	-0.02	-0.09	0.08	0.1008	0.09	0.05	0.16	0.1008
	Data rich	2,072	74	0.01	-0.08	0.08	0.1157	0.08	0.04	0.14	0.1157
Hold out pattern	Post-2000 data removed	1,012	79	0.00	-0.09	0.08	0.6130	0.08	0.04	0.14	0.6130
	Random set of data removed	1,425	71	0.01	-0.08	0.09	0.1596	0.08	0.04	0.15	0.1596

Mean HDL cholesterol, Test 2, Men											
Data		No. of held out observations	Percent covered	Error (mmol/L)†				Absolute error (mmol/L)			
				Median	Q1	Q3	(p*)	Median	Q1	Q3	(p*)
All		2,382	79	0.01	-0.06	0.07	0.0287	0.07	0.03	0.13	0.0287
Super-region	Central and eastern Europe	159	86	0.01	-0.03	0.06	0.0422	0.06	0.02	0.10	0.0422
	Central Asia, Middle East and north Africa	327	66	0.02	-0.08	0.10	0.1354	0.09	0.04	0.16	0.1354
	East and southeast Asia	212	75	0.02	-0.06	0.09	0.0344	0.08	0.04	0.15	0.0344
	High-income Asia Pacific	242	88	-0.03	-0.08	0.00	0.0000	0.04	0.02	0.08	0.0000
	High-income western countries	1,007	80	0.01	-0.05	0.07	0.0028	0.06	0.03	0.11	0.0028
	Latin America and the Caribbean	255	85	0.00	-0.08	0.07	0.5987	0.08	0.03	0.14	0.5987
	Oceania	40	60	-0.02	-0.19	0.10	0.2424	0.14	0.04	0.23	0.2424
	South Asia	121	75	0.04	-0.02	0.12	0.0014	0.09	0.04	0.17	0.0014
	Sub-Saharan Africa	19	84	0.01	-0.08	0.11	0.6226	0.10	0.07	0.15	0.6226
Urban or rural studies	Rural	246	79	0.01	-0.07	0.09	0.2752	0.08	0.03	0.14	0.2752
	Urban	693	73	0.02	-0.06	0.09	0.0470	0.08	0.03	0.13	0.0470
	Both rural and urban	1,443	81	0.00	-0.06	0.06	0.4108	0.06	0.03	0.12	0.4108
Study representativeness	Community	861	74	0.01	-0.07	0.09	0.0921	0.08	0.04	0.14	0.0921
	Subnational	483	80	0.01	-0.06	0.08	0.0082	0.07	0.03	0.13	0.0082
	National	1,038	82	0.00	-0.06	0.05	0.8147	0.06	0.02	0.11	0.8147
Age band	18-40	720	78	0.02	-0.05	0.08	0.0019	0.07	0.03	0.13	0.0019
	40-60	740	76	0.00	-0.07	0.07	0.5225	0.07	0.03	0.13	0.5225
	60 and above	922	82	0.00	-0.06	0.06	0.9069	0.06	0.03	0.12	0.9069
Years	1980-1989	186	61	0.05	-0.04	0.14	0.0001	0.10	0.04	0.18	0.0001
	1990-1999	221	73	0.02	-0.06	0.08	0.0650	0.08	0.04	0.14	0.0650
	2000-2009	1,144	80	0.00	-0.07	0.06	0.3022	0.06	0.03	0.12	0.3022
	2010-2018	831	83	0.00	-0.06	0.07	0.1440	0.06	0.03	0.12	0.1440
Data density	Average data density	353	82	-0.02	-0.12	0.07	0.0022	0.10	0.04	0.16	0.0022
	Data rich	2,029	78	0.01	-0.05	0.07	0.0001	0.06	0.03	0.12	0.0001
Hold out pattern	Post-2000 data removed	901	91	0.00	-0.05	0.06	0.2062	0.05	0.03	0.10	0.2062
	Random set of data removed	1,481	71	0.01	-0.07	0.08	0.0778	0.08	0.03	0.14	0.0778

TC, total cholesterol; HDL, high-density lipoprotein; Q1, first quartile; Q3, third quartile; p, p value.

† Estimated values minus held out values.

* p values for model error comparisons were calculated using the non-parametric Wilcoxon signed-rank test (two-sided) for paired data. The p values are calculated assuming independence of the held-out observations. They should therefore be interpreted as an approximation because there is some dependence among the held-out observations, within each of the five repetitions for example.

Supplementary Figure 1. Flowchart of literature search for additional data sources.

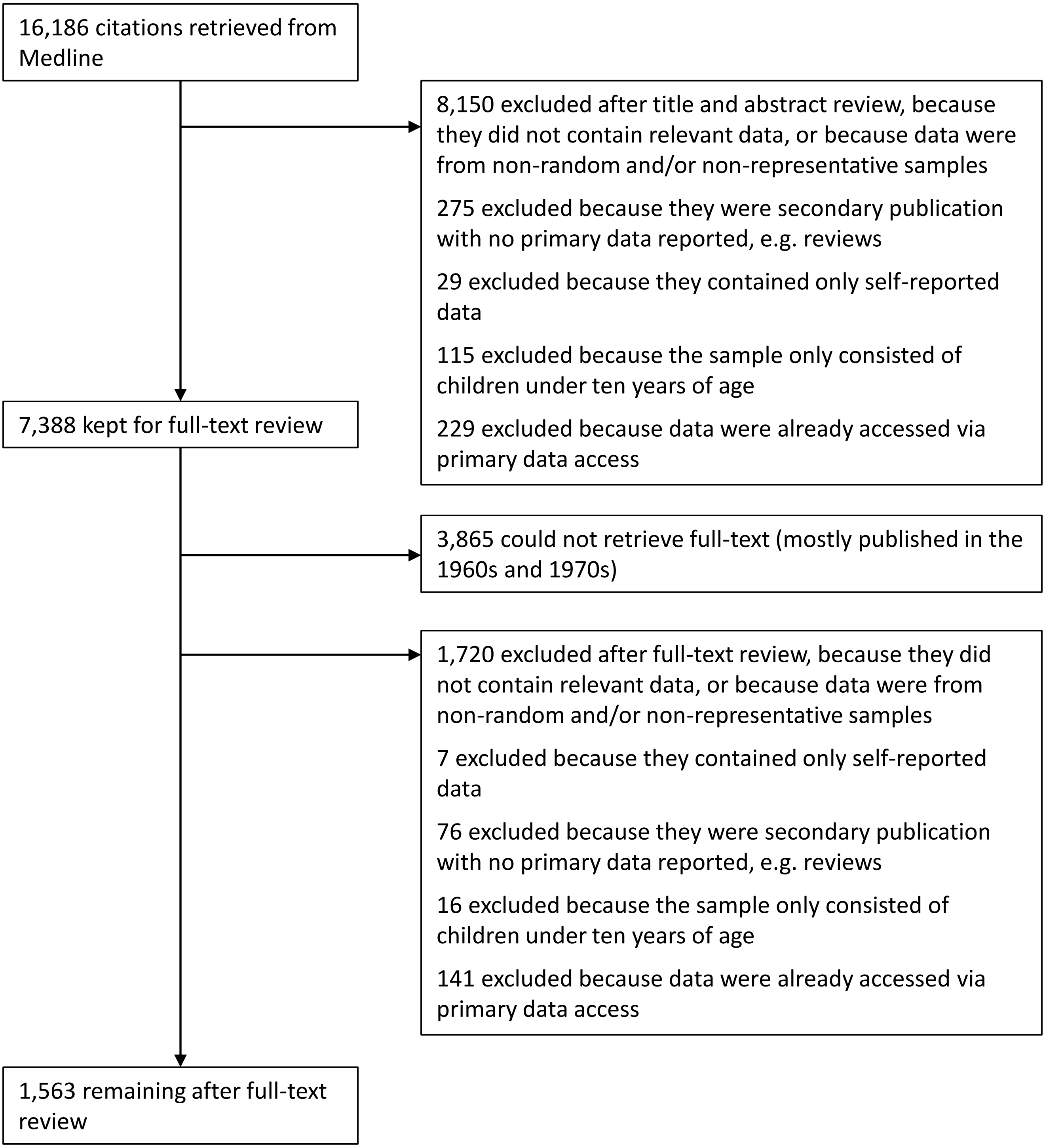
16,186 citations retrieved from Medline
8,150 excluded after title and abstract review, because they did not contain relevant data, or because data were from non-random and/or non-representative samples
275 excluded because they were secondary publication with no primary data reported, e.g. reviews
29 excluded because they contained only self-reported data
115 excluded because the sample only consisted of children under ten years of age
229 excluded because data were already accessed via primary data access
7,388 kept for full-text review
3,865 could not retrieve full-text (mostly published in the 1960s and 1970s)
1,720 excluded after full-text review, because they did not contain relevant data, or because data were from non-random and/or non-representative samples
7 excluded because they contained only self-reported data
76 excluded because they were secondary publication with no primary data reported, e.g. reviews
16 excluded because the sample only consisted of children under ten years of age
141 excluded because data were already accessed via primary data access
1,563 remaining after full-text review

References

1. NCD Risk Factor Collaboration (NCD-RisC). Trends in adult body-mass index in 200 countries from 1975 to 2014: a pooled analysis of 1698 population-based measurement studies with 19.2 million participants. *Lancet* **387**, 1377-1396 (2016).
2. NCD Risk Factor Collaboration (NCD-RisC). A century of trends in adult human height. *eLife* **5**, e13410 (2016).
3. NCD Risk Factor Collaboration (NCD-RisC). Worldwide trends in diabetes since 1980: a pooled analysis of 751 population-based studies with 4.4 million participants. *Lancet* **387**, 1513-1530 (2016).
4. NCD Risk Factor Collaboration (NCD-RisC). Worldwide trends in blood pressure from 1975 to 2015: a pooled analysis of 1479 population-based measurement studies with 19.1 million participants. *Lancet* **389**, 37-55 (2017).
5. NCD Risk Factor Collaboration (NCD-RisC). Worldwide trends in body-mass index, underweight, overweight, and obesity from 1975 to 2016: a pooled analysis of 2416 population-based measurement studies in 128.9 million children, adolescents, and adults. *Lancet* **390**, 2627-2642 (2017).
