# Supplementary material for: Repositioning of the global epicentre of non-optimal cholesterol
Source: Nature. 2020 Jun 3;582(7810):73–7. doi: 10.1038/s41586-020-2338-1 (PMC7332422; doi:10.1038/s41586-020-2338-1)
Supplement: Supplementary file 2 — Reporting Summary [file 41586_2020_2338_MOESM2_ESM.pdf]

nature research

Corresponding author(s): Majid Ezzati

Last updated by author(s): Mar 19, 2020

Reporting Summary

Nature Research wishes to improve the reproducibility of the work that we publish. This form provides structure for consistency and transparency in reporting. For further information on Nature Research policies, see Authors & Referees and the Editorial Policy Checklist.

Statistics

For all statistical analyses, confirm that the following items are present in the figure legend, table legend, main text, or Methods section.

n/a	Confirmed	
☒	☐	The exact sample size (*n*) for each experimental group/condition, given as a discrete number and unit of measurement
☒	☐	A statement on whether measurements were taken from distinct samples or whether the same sample was measured repeatedly
☐	☒	The statistical test(s) used AND whether they are one- or two-sided<br>*Only common tests should be described solely by name; describe more complex techniques in the Methods section.*
☒	☐	A description of all covariates tested
☒	☐	A description of any assumptions or corrections, such as tests of normality and adjustment for multiple comparisons
☐	☒	A full description of the statistical parameters including central tendency (e.g. means) or other basic estimates (e.g. regression coefficient) AND variation (e.g. standard deviation) or associated estimates of uncertainty (e.g. confidence intervals)
☒	☐	For null hypothesis testing, the test statistic (e.g. *F*, *t*, *r*) with confidence intervals, effect sizes, degrees of freedom and *P* value noted<br>*Give P values as exact values whenever suitable.*
☐	☒	For Bayesian analysis, information on the choice of priors and Markov chain Monte Carlo settings
☒	☐	For hierarchical and complex designs, identification of the appropriate level for tests and full reporting of outcomes
☐	☒	Estimates of effect sizes (e.g. Cohen's *d*, Pearson's *r*), indicating how they were calculated

Our web collection on statistics for biologists contains articles on many of the points above.

Software and code

Policy information about availability of computer code

Data collection	Processing of secondary data was conducted using the statistical software R (version 3.6.0).
Data analysis	All analyses were conducting using the statistical software R (version 3.6.0). The code for estimation of mean risk factor trends is available at www.ncdrisc.org.

For manuscripts utilizing custom algorithms or software that are central to the research but not yet described in published literature, software must be made available to editors/reviewers. We strongly encourage code deposition in a community repository (e.g. GitHub). See the Nature Research guidelines for submitting code & software for further information.

Data

Policy information about availability of data

All manuscripts must include a data availability statement. This statement should provide the following information, where applicable:

- Accession codes, unique identifiers, or web links for publicly available datasets
- A list of figures that have associated raw data
- A description of any restrictions on data availability

This is a data-pooling study that brings together more than 1000 disparate data sources and uses a Bayesian hierarchical model to estimate population risk factor trends. Estimates of mean total, non-HDL and HDL cholesterol by country, year, and sex will be available from www.ncdrisc.org upon the publication of the paper. Some of the input data sources are publicly available, for which we will add links in the final version of the paper. Others are the property of specific research groups and agencies, for which we will provide contact information.

Field-specific reporting

Please select the one below that is the best fit for your research. If you are not sure, read the appropriate sections before making your selection.

☐ Life sciences ☒ Behavioural & social sciences ☐ Ecological, evolutionary & environmental sciences

For a reference copy of the document with all sections, see nature.com/documents/nr-reporting-summary-flat.pdf

Behavioural & social sciences study design

All studies must disclose on these points even when the disclosure is negative.

Study description	We pooled and re-analysed population-based data that had measured blood lipids in adults to estimate trends in mean total, non-HDL and HDL cholesterol from 1980 to 2018 for 200 countries and territories, using a Bayesian hierarchical model.
Research sample	We pooled data from 1,127 population-based studies of blood lipids conducted in 161 countries, with measurement of blood lipids in over 102 million adults aged 18 years and older. Studies were representative of a national, subnational or community population.
Sampling strategy	We included data collected using a probabilistic sampling method with a defined sampling frame. We therefore included studies with simple random and complex survey designs but excluded convenience samples.
Data collection	We used data on measured blood lipids to calculate mean total, non-HDL and HDL cholesterol. We excluded self-reported data.
Timing	We pooled data collected from 1980 to 2018. We also included national studies for the 3 years prior to 1980 (n=1), assigning them to 1980, so that they can inform the estimates in countries with slightly earlier national data.
Data exclusions	We excluded all data sources that included only hypercholesterolemia or dyslipidaemia diagnosis history or medication status without measurement of cholesterol levels. We also excluded data sources on population subgroups whose lipid profile may differ systematically from the general population, including:<br>• studies that had included or excluded people based on their health status or cardiovascular risk;<br>• studies whose participants were only ethnic minorities;<br>• specific educational, occupational, or socioeconomic subgroups, with the exception noted below;<br>• those recruited through health facilities, with the exception noted below.<br><br>We used school-based data in countries, and in age-sex groups, where secondary school enrollment was 70% or higher. We used data whose sampling frame was health insurance schemes in countries where at least 80% of the population were insured. Finally, we used data collected through general practice and primary care systems in high-income and central European countries with universal insurance, because contact with the primary care systems tends to be as good as or better than response rates for population-based surveys. Our exclusion criteria were established at the initiation of the study to ensure all data were representative.
Non-participation	Our inclusion/exclusion criteria were designed to ensure participants of the surveys included were representative of the general population from which each sample was drawn.
Randomization	Our study is descriptive, and we did not carry out experiments.

Reporting for specific materials, systems and methods

We require information from authors about some types of materials, experimental systems and methods used in many studies. Here, indicate whether each material, system or method listed is relevant to your study. If you are not sure if a list item applies to your research, read the appropriate section before selecting a response.

Materials & experimental systems

n/a	Involved in the study	
☒	☐	Antibodies
☒	☐	Eukaryotic cell lines
☒	☐	Palaeontology
☒	☐	Animals and other organisms
☒	☐	Human research participants
☒	☐	Clinical data

Methods

n/a	Involved in the study	
☒	☐	ChIP-seq
☒	☐	Flow cytometry
☒	☐	MRI-based neuroimaging
